# Cost-benefit analysis of a potential hepatitis C vaccine: a modelling study

**DOI:** 10.1186/s12879-026-13858-7

**Published:** 2026-07-02

**Authors:** Farah Houdroge, Phillip Luong, Jessica Zuk, Chris Seaman, Kelly Maynard, Heidi Drummer, Paul Zimmer-Harwood, Circe McDonald, Nick Scott

**Affiliations:** 1 Disease Elimination Program, Burnet Institute, 85 Commercial Road, Melbourne, VIC 3004 Australia; 2 Department of Microbiology and Immunology, Peter Doherty Institute for Infection and Immunity, University of Melbourne, Melbourne, VIC Australia; 3 Department of Microbiology, Monash University, Clayton, VIC Australia; 4 1Day Sooner Research Team, Claymont, DE USA; 5 School of Public Health and Preventive Medicine, Monash University, Melbourne, Australia

**Keywords:** Hepatitis C, Vaccine, Mathematical model, Cost-benefit analysis, Cost-utility analysis

## Abstract

**Background:**

The increased availability and cost-reduction of curative hepatitis C therapies and global commitments to elimination through treatment scale-up have made the value proposition for a hepatitis C vaccine unclear. This study aimed to estimate the health impact and benefit-cost ratio of a potential hepatitis C vaccine under different use scenarios.

**Methods:**

A compartmental model of hepatitis C transmission, disease progression and care cascade was calibrated to 116 countries, with populations stratified by age, sex, injection drug use status and incarceration status. Model inputs were taken from global datasets (e.g. United Nations, Institute of Health Metrics and Evaluation), published academic literature and WHO reports. Scenarios projected for 2026–2050 included: (1) test-treat-vaccinate for people who inject drugs at existing country-specific testing coverage; (2) Scenario 1 plus a one-off test-treat-vaccinate campaign for people who inject drugs; (3) Scenario 2 plus test-treat-vaccinate of prison populations; (4) Scenario 3 plus school-based vaccination; and (5) Scenario 4 plus a five-year adult catch-up campaign. Each scenario’s comparator was a counterfactual with equivalent testing and treatment but no vaccine. Only RNA-negative individuals were eligible for the vaccine (i.e. following negative test or post-treatment), and the vaccine was modelled to increase spontaneous clearance rates to 80% for a mean five-year period. Vaccination costs included commodity (assumed US$5 per person in low and lower-middle income settings, with cost ratio of 1:2:15 for upper-middle and high income settings), service delivery and programmatic costs. Benefits included reduced confirmatory testing, treatment, disease management and productivity losses.

**Results:**

Globally, scenarios 1–5 averted 1.13–8.76 million infections and 2,490–24,100 deaths. Scenarios 1, 2 and 3 had benefit-cost ratios of 2.86, 3.24, and 2.20 respectively. Scenario 4 only had a benefit-cost ratio > 1 for a vaccine with longer duration of protection. Scenario 5 had a benefit-cost ratio < 1 for all characteristics tested.

**Conclusions:**

In the context of heterogeneous treatment scale-up among countries, a hepatitis C vaccine is likely to have a positive return-on-investment when used to vaccinate populations at highest risk of infection.

**Trial registration:**

Not applicable.

**Supplementary Information:**

The online version contains supplementary material available at https://doi.org/10.1186/s12879-026-13858-7.

## Background

In 2015, there were an estimated 71 million people living with hepatitis C, 1.75 million new infections and 402,000 deaths globally [1]. The discovery of direct-acting antiviral (DAA) treatment led to much excitement, and in 2016 the World Health Organization (WHO) set 2030 targets of reducing new hepatitis C infections by 80% and hepatitis C-related deaths by 65%, compared to 2015 levels [2]. While significant progress has been made, it has not been equitable and challenges persist including limited scale-up of prevention, costs associated with testing and treatment, and barriers to service delivery for the populations most affected [3]. In 2022 there were still an estimated 50 million people living with hepatitis C, as well as 1.0 million new hepatitis C infections and 221,000 hepatitis C-related deaths globally [3]. Only 36% of people living with hepatitis C had been diagnosed between 2015-2022, and 20% received treatment [3].

Vaccines for hepatitis C have been in pre-clinical development since the discovery of the hepatitis C virus more than 30 years ago. Much progress has been made, but relatively few vaccines have entered clinical trials and none have progressed beyond phase 2 [4]. Commercial interest in hepatitis C vaccine development has waned following the discovery of DAAs, given the uncertainty that DAAs created around the potential use case, impact and return-on-investment of a vaccine. A decade on, there is more certainty: testing and treatment is failing to achieve the promised impacts [5], and there is a renewed interest to quantify the health and economic benefits that a vaccine could provide.

A prophylactic vaccine could have different use cases for different settings. In most high-income countries, hepatitis C transmission primarily occurs among people who inject drugs through unsafe injecting practices [3], and current testing and treatment programs (where available) could be adapted to include vaccination after a negative test or treatment completion. In many low- and middle-income countries, transmission also still occurs through official and unofficial health care systems, as well as other community exposures (e.g. tattooing, barbershops or cultural practices) [3]. In these settings, it is possible that in addition to vaccination programs for people who inject drugs, adolescent and adult vaccination programs would be needed, as a way of protecting the broader population.

Previous modelling studies have considered the potential impact of a hepatitis C vaccine, suggesting that even modest coverage of a low-efficacy vaccine among people who inject drugs could provide significant additional prevalence reduction beyond treatment alone [6], and would likely reduce the cost, and increase the achievability of global elimination targets [7]. However, these studies were undertaken before the global scale-up of DAAs.

In the context of heterogeneous DAA scale-up among countries and population groups, this study aims to calculate the potential health impact and benefit-cost ratio of hepatitis C vaccines in different use case scenarios.

## Methods

### Mathematical model

#### Settings

The modelling included 116 countries with sufficient population and epidemiological data, representing more than 90% of the global population [8] and hepatitis C cases in 2022 [3]. By WHO region, this included 20 countries in the African Region, 15 in the Region of the Americas, 6 in the South-East Asia Region, 43 in the European Region, 18 in the Eastern Mediterranean Region, and 14 in the Western Pacific Region. A full list of countries, details on data sources and imputation method, and other global model parameters are provided in Supplement Section A (Tables 1, 2 and 3).

**Table 1:** Description of interventions, scenarios and sensitivity analyses considered

Scenarios	Description	Coverage
**Modelled scenarios**		
**Scenario 1**	Status-quo + vaccination of people who inject drugs.	Vaccine coverage among people who inject drugs (RNA negative) to match existing hepatitis C testing coverage between 2026 and 2050.
**Scenario 2**	Scenario 1 + scale-up campaign for people who inject drugs.	A one-year test (regardless of status), treat (RNA positive) and vaccinate (RNA negative) campaign reaching 80% of people who inject drugs between 2026 and 2027.
**Scenario 3**	Scenario 2 + prison scale-up.	Implementation of an ongoing test (regardless of status), treat (RNA positive) and vaccinate (RNA negative) program to reach 80% of all people in prisons between 2026 and 2050.
**Scenario 4**	Scenario 3 + school-based vaccination.	Vaccination of adolescents (RNA negative) through existing school-based immunisation programs [9], with coverage to match that of the HPV vaccine [10].
**Scenario 5**	Scenario 4 + adult catch-up campaign.	A five-year vaccination campaign to reach 80% of 18–64-year-olds (RNA negative) in the general population between 2026 and 2031.
**Counterfactual comparators**		
**Counterfactual 1** (to scenario 1)	Status-quo.	No changes to existing testing and treatment between 2026 and 2050.
**Counterfactual 2** (to scenario 2)	Counterfactual 1 + test and treat scale-up campaign for people who inject drugs.	A one-year test and treat campaign reaching 80% of people who inject drugs between 2026 and 2027.
**Counterfactual 3** (to scenarios 3–5)	Counterfactual 2 + test and treat scale-up in prisons.	Implementation of an ongoing test and treat program to reach 80% of people in prisons between 2026 and 2050.
**Univariate sensitivity analyses**		
**Vaccine efficacy** (baseline: 80%, equally across all genotypes)	Spontaneous clearance rates of 50% and 100%.	
	Efficacy weighted by genotype distribution (100% against one genotype, 0% against others).*	
	Trivalent genotype-specific efficacy (100% against genotypes 1, 3 and 4 and 0% others).*	
	Efficacy of 40% or 60% in previously infected individuals.	
**Duration of protection** (baseline: 5 years)	1 year.	
	10 years.	
	30 years.	
	100 years (lifelong).	
**Vaccine coverage (scenarios 2 and 3)** † (baseline: 80%)	20%	
	40%	
	60%	
**Vaccine unit cost** ‡ (baseline: $5 in LLMIC)	$0, $10, $15, $20, $25 and $30.	
**DAA cost** (baseline: $60)	$5000 in HIC, $60 in LLMIC and UMIC.	
	$30 in all settings.	
**Engagement in care** (baseline: 25% all stages, except cirrhosis and above 75% in HIC)§	Baseline values halved or doubled (capped at 100%).	
**Employment rate for people who inject drugs** (baseline: same as general population)	0%	
	50% lower rate than the general population.	

Abbreviations: LLMIC: Low and Lower-Middle Income Countries; HIC: High Income Countries.

* Assuming random vaccination of individuals without prior genotype knowledge. Efficacy was constrained not to fall below the natural spontaneous clearance rate. Regional genotype distributions were extracted from [11]. Selection of genotypes for trivalent vaccine efficacy was based on the three most prevalent genotypes globally, covering 83.8% of the pandemic [11].

† Coverage was constrained not to fall below existing levels of testing and treatment in each setting.

‡ Vaccine costs maintained the unit cost ratio of 1:2:15 in low and lower-middle, upper-middle and high income settings.

§ See appendix, Section B

**Table 2:** Model outcomes on a global scale for the five scenarios. Outcomes are aggregated from 2026 to 2050. Costs are presented in 2024 US dollars, with 3% annual discounting

Outcomes 2026–2050	Scenario 1	Scenario 2	Scenario 3	Scenario 4	Scenario 5
**Global (n=116 countries)**					
Vaccines administered	17.5M (13.5M to 20.8M)	21.9M (18.3M to 25M)	44.6M (42.5M to 46.5M)	404M (402M to 406M)	8.26B (8.25B to 8.26B)
Vaccine-averted HCV infections	1.13M (825k to 1.42M)	1.58M (1.38M to 1.8M)	2.36M (2.25M to 2.49M)	2.91M (2.77M to 3.09M)	8.76M (8.05M to 9.75M)
Vaccine-averted HCC cases	1.41k (562 to 2.12k)	3.67k (1.5k to 5.06k)	4.5k (1.81k to 6.07k)	4.82k (2.04k to 6.46k)	15.9k (5.2k to 24.2k)
Vaccine-averted HCV deaths	2.49k (1k to 3.69k)	5.66k (2.26k to 7.89k)	7.4k (2.92k to 10k)	7.9k (3.28k to 10.7k)	24.1k (8.21k to 37.8k)
Vaccine-averted DALYs	13.7k (5.62k to 20.9k)	35.3k (14.2k to 51.3k)	46.2k (18.2k to 66.2k)	48.3k (19.5k to 68.8k)	126k (44.9k to 193k)
Vaccine-averted direct costs ($)	283M (187M to 552M)	471M (335M to 844M)	642M (469M to 1.1B)	645M (471M to 1.11B)	767M (547M to 1.36B)
Vaccine-attributable productivity gains ($)	2.53B (1.86B to 3.22B)	3.24B (2.63B to 3.83B)	3.54B (3.02B to 3.97B)	3.67B (3.16B to 4.1B)	6.59B (5.89B to 7.32B)
Vaccine costs ($)	985M (857M to 1.08B)	1.15B (1.03B to 1.23B)	1.9B (1.83B to 1.96B)	8.11B (8.04B to 8.17B)	203B (203B to 203B)
ICER ($)	51.5k (24.9k to 126k)	19.1k (6.93k to 48.6k)	27.3k (13.4k to 72.7k)	154k (104k to 384k)	1.61M (1.05M to 4.5M)
% countries where ICER ≤ 0.5, 1, 2 ×GDP per capita	34%, 43%, 54%	44%, 56%, 62%	37%, 44%, 52%	5%, 10%, 13%	3%, 3%, 3%
Benefit-cost ratio	2.86 (2.2 to 3.76)	3.24 (2.68 to 4.06)	2.2 (1.88 to 2.63)	0.53 (0.46 to 0.63)	0.04 (0.03 to 0.04)

Abbreviations: HCV: hepatitis C virus; HCC: hepatocellular carcinoma; DALY: disability-adjusted life year; ICER: incremental cost-effectiveness ratio (as incremental cost per DALY averted); k: thousand; M: million; B: billion

#### Model description

**Fig. 1:**
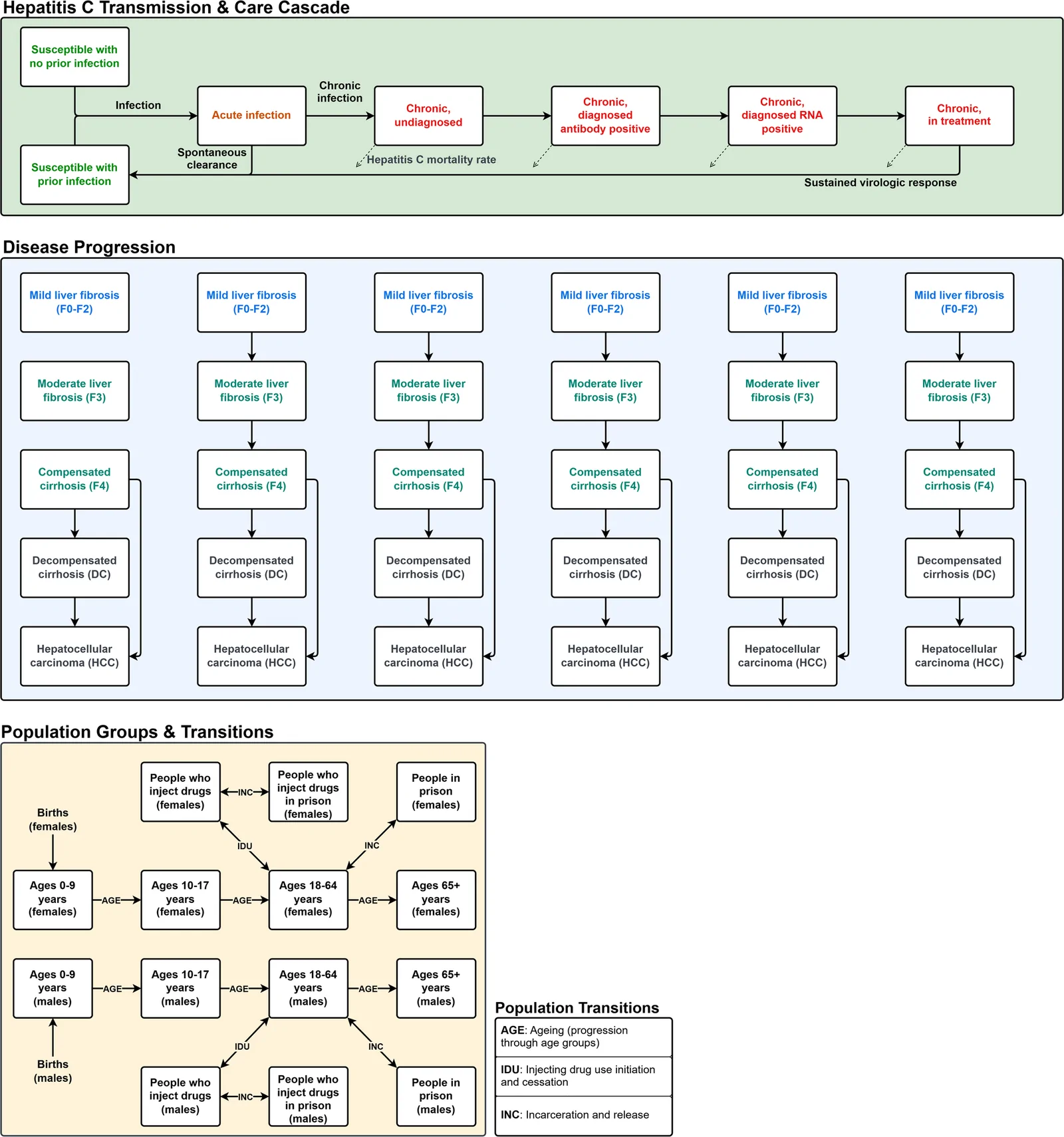
Model schematic

The mathematical model used for this analysis is a dynamic, compartmental model of hepatitis C virus transmission in adults, adapted from previous studies [12, 13]. Country populations were stratified by sex (female and male), age group (0–9, 10–17, 18–64 and 65 + years) and risk group (people who inject drugs, people in prison, and the remainder of the general population). Transitions between population groups occurred through ageing, initiation or cessation of injecting drug use, and entry into or release from prison (Fig. 1).

At any given time, individuals in the model were assigned to one of the following compartments: susceptible with no prior infection, susceptible with prior infection cleared either spontaneously or through treatment, acute infection, or chronic infection. Chronic infection progressed through stages of liver disease from no fibrosis (F0) to liver cirrhosis (F4), decompensated cirrhosis (DC) and hepatocellular carcinoma (HCC). Individuals with acute or chronic infection were further classified as undiagnosed, diagnosed antibody-positive, diagnosed RNA-positive, or on treatment. Transition rates between compartments were both country- and population-specific.

People entered the model as susceptible (no prior infection) based on the annual number of live births and exited according to age-specific all-cause, and hepatitis C-related, mortality rates. Net migration rates were incorporated to capture whole-population inflows and outflows.

Among people who inject drugs in the community or in prison, hepatitis C transmission was modelled dynamically based on the RNA prevalence within each specific population group. In settings where RNA prevalence exceeded 2% in the general population, dynamic transmission was also included for adults aged 18–64 and 65 years and older to account for ongoing transmission in the broader community [14]. The model was run from 2000 to 2050.

Model code, including country-level inputs, is available online [15].

#### Input data

Estimates of births, population size, mortality rates and net migration were obtained from the United Nations Population Division [8]. Country- and regional-level estimates of the number of people who inject drugs (and proportion male/female) were sourced from a global systematic review [16], and data on the number of people in prison from the World Prison Brief [17, 18].

The model incorporated global estimates of disease progression rates [19] and sex-specific spontaneous clearance rates [20–22]. Country-specific hepatitis C RNA prevalence estimates among people who inject drugs were obtained from published systematic reviews [16, 23]. The RNA prevalence among people in prison was estimated as a weighted average of the prevalence in people who inject drugs and in the general population, based on the proportion of each group within the prison population [16]. In the absence of sex-disaggregated data, we assumed equal hepatitis C prevalence between females and males.

The country-specific annual proportion of people who inject drugs tested for hepatitis C was derived from a systematic review on HIV and hepatitis C testing and treatment [24]. In prisons, we assumed that only countries that had implemented a harm reduction program in at least one facility in 2022 [25] offered hepatitis C testing and treatment. The number of treatments initiated [26] was distributed across population groups based on hepatitis C burden and disease stage.

One thousand simulations were performed, by sampling from the distribution of parameters with uncertainty (the number of treatments and the rates of testing, spontaneous clearance and disease progression). Additional details on the population groups (including transitions between groups), disease burden, care cascade, disease parameter inputs and simulation convergence checks can be found in Supplement Section A (Tables 1 and 2; Fig. 1).

**Table 3:** Benefit-cost ratios for the five vaccination scenarios from 2026 to 2050. Results are presented for the WHO defined regions as well as the ten countries with the highest hepatitis C burden

Benefit-cost ratios2026–2050	Scenario 1	Scenario 2	Scenario 3	Scenario 4	Scenario 5
African Region	4.49 (2.84 to 5.18)	4.75 (4.06 to 5.59)	2.06 (1.83 to 2.41)	0.10 (0.09 to 0.11)	0.05 (0.05 to 0.06)
Region of the Americas	2.34 (1.68 to 3.30)	2.09 (1.59 to 2.86)	1.26 (1.02 to 1.59)	0.44 (0.35 to 0.55)	0.04 (0.03 to 0.04)
Eastern Mediterranean Region	7.40 (6.21 to 11.45)	6.59 (5.56 to 8.24)	4.41 (3.82 to 5.20)	0.20 (0.17 to 0.24)	0.06 (0.04 to 0.09)
European Region	3.44 (2.97 to 3.98)	5.36 (4.70 to 6.34)	4.03 (3.54 to 4.80)	0.95 (0.84 to 1.12)	0.04 (0.03 to 0.04)
South East Asia Region	3.44 (2.63 to 5.34)	4.56 (3.53 to 7.01)	3.03 (2.72 to 3.89)	0.12 (0.12 to 0.15)	0.04 (0.03 to 0.04)
Western Pacific Region	7.20 (5.76 to 9.17)	7.36 (6.07 to 9.28)	3.61 (3.07 to 4.31)	0.78 (0.66 to 0.92)	0.02 (0.02 to 0.03)
High Burden Countries	2.33 (1.69 to 3.26)	2.50 (1.99 to 3.29)	1.59 (1.33 to 1.94)	0.53 (0.45 to 0.64)	0.04 (0.04 to 0.05)

#### Model calibration and validation

Model calibration was performed using a maximum likelihood optimisation routine [27].

Injecting drug use initiation rates were adjusted to match population size estimates of people who inject drugs, assuming an average injecting career length of 17 years across all settings [28]. Prison population size was calibrated by adjusting the release rate, as data on prison admissions were more readily available.

The testing rate in the general population was varied to align with the annual number of new diagnoses [29]. Population-specific force of infection constants were adjusted to match prevalence estimates among people who inject drugs and people in prison, as well as the total number of hepatitis C cases nationally [29]. Treatment allocations across population groups were varied to reflect the observed epidemic trends and total treatment numbers [26].

Finally, disease progression and hepatitis C-related mortality rates were adjusted to reproduce the annual number of hepatitis C-related deaths [30].

Model calibration outcomes were validated against incidence estimates from the 2024 Global Hepatitis Report by the WHO [3] and the Polaris Observatory by the CDA Foundation [29].

#### Vaccine intervention

As the vaccine does not yet exist, its characteristics are unknown. For the main analysis, the vaccine was modelled to increase the probability of spontaneous clearance within one year to 80% among vaccinated individuals who become infected, irrespective of sex, with immunity lasting for a mean of 5 years. The vaccine was modelled to be equally efficacious across all genotypes (tested in sensitivity analysis). Since spontaneous clearance in the baseline differed by sex, this is assuming unequal relative impact. This mechanism of action (i.e. increasing spontaneous clearance) is based on vaccine development efforts that focus on inducing immune-mediated clearance of acute infection to prevent progression to chronic infection, rather than to prevent infection altogether. The effectiveness assumptions were based on expert opinion in the absence of robust data from clinical trials, and tested in one-way sensitivity analyses (Table 1). Individuals were eligible for vaccination if they had no current infection (e.g. if they were in the susceptible compartment, either with or without prior cleared infection). The model compartments described previously were replicated for vaccinated individuals.

#### Scenarios projected

The scenarios considered for each country are summarised in Table 1. Delivery of the vaccine depends on the coverage of testing and treatment programs, but for this analysis we are only interested in the value added by the vaccine. To isolate the added impact of the vaccine, each scenario was compared to a counterfactual with equivalent testing and treatment scale-up but without vaccination. Scenarios should therefore be interpreted as the value added by a vaccine in different testing and treatment contexts, rather than as being incremental to one another.

#### Outcome measures

The primary epidemiological and health outcomes were the projected number of incident hepatitis C infections, HCC cases, hepatitis C-related deaths, and disability-adjusted life years (DALYs). For each country, the model was run 1000 times by sampling from the distribution of parameters with uncertainty. Results are presented as a central estimate from the calibrated run, with a 95% confidence interval (CI) derived from the sampled runs. Supplement Section A.

### Economic analysis

The economic evaluation was conducted from a societal perspective, capturing both direct healthcare costs and productivity impacts. Costs are in 2024 Consumer Price Index-adjusted United States dollars (US$), with all prospective costs discounted at 3% per annum in line with WHO guidelines [31]. To reflect economic uncertainty, each simulation drew a random sample from the uncertainty distributions of the cost parameters. Supplement Section B, Table 4 provides a comprehensive overview of costing methods, assumptions, and input values.

**Table 4:** One-way sensitivity analyses. Impact on the central benefit-cost ratios for the five scenarios

Benefit-cost ratios 2026–2050	Scenario 1	Scenario 2	Scenario 3	Scenario 4	Scenario 5
*Base case*	2.86	3.24	2.20	0.53	0.04
*Vaccine efficacy 50% vs. 80% against all genotypes*	1.30 (-54.5%)	1.45 (-55.2%)	1.01 (-54.1%)	0.24 (-54.7%)	0.02 (-50.0%)
*Vaccine efficacy 100% vs. 80% against all genotypes*	3.86 (+ 35.0%)	4.44 (+ 37.0%)	2.97 (+ 35.0%)	0.72 (+ 35.8%)	0.05 (+ 25.0%)
*100% efficacy against genotype 1 vs. 80% against all genotypes*	1.98 (-30.8%)	2.17 (-33.0%)	1.49 (-32.3%)	0.35 (-34.0%)	0.02 (-50.0%)
*100% efficacy against genotype 2 vs. 80% against all genotypes*	0.02 (-99.3%)	0.05 (-98.5%)	0.06 (-97.3%)	0.01 (-98.1%)	0.00 (-100.0%)
*100% efficacy against genotype 3 vs. 80% against all genotypes*	0.11 (-96.2%)	0.15 (-95.4%)	0.13 (-94.1%)	0.03 (-94.3%)	0.00 (-100.0%)
*100% efficacy against genotype 4 vs. 80% against all genotypes*	0.02 (-99.3%)	0.04 (-98.8%)	0.03 (-98.6%)	0.01 (-98.1%)	0.00 (-100.0%)
*100% efficacy against genotype 5 vs. 80% against all genotypes*	-0.01 (-100.3%)	-0.01 (-100.3%)	-0.00 (-100.0%)	-0.00 (-100.0%)	-0.00 (-100.0%)
*100% efficacy against genotype 6 vs. 80% against all genotypes*	-0.01 (-100.3%)	-0.01 (-100.3%)	-0.00 (-100.0%)	0.00 (-100.0%)	-0.00 (-100.0%)
*100% efficacy against genotypes 1*,*3*,*4 vs. 80% against all genotypes*	3.19 (+ 11.5%)	3.60 (+ 11.1%)	2.42 (+ 10.0%)	0.58 (+ 9.4%)	0.04 (0.0%)
*Vaccine efficacy prior exposure 40% vs. 80%*	2.47 (-13.6%)	2.65 (-18.2%)	1.71 (-22.3%)	0.42 (-20.8%)	0.03 (-25.0%)
*Vaccine efficacy prior exposure 60% vs. 80%*	2.66 (-7.0%)	2.93 (-9.6%)	1.95 (-11.4%)	0.47 (-11.3%)	0.03 (-25.0%)
*Duration of protection 1 year vs. 5 years*	0.59 (-79.4%)	0.64 (-80.2%)	0.42 (-80.9%)	0.11 (-79.2%)	0.01 (-75.0%)
*Duration of protection 10 years vs. 5 years*	4.25 (+ 48.6%)	4.92 (+ 51.9%)	3.45 (+ 56.8%)	0.79 (+ 49.1%)	0.06 (+ 50.0%)
*Duration of protection 30 years vs. 5 years*	6.08 (+ 112.6%)	7.19 (+ 121.9%)	5.26 (+ 139.1%)	1.11 (+ 109.4%)	0.08 (+ 100.0%)
*Duration of protection lifelong vs. 5 years*	7.13 (+ 149.3%)	8.49 (+ 162.0%)	6.37 (+ 189.5%)	1.27 (+ 139.6%)	0.09 (+ 125.0%)
*20% coverage (scenarios 2 and 3) vs. 80%*	2.86 (0.0%)	2.95 (-9.0%)	2.96 (+ 34.5%)	0.53 (0.0%)	0.04 (0.0%)
*40% coverage (scenarios 2 and 3) vs. 80%*	2.86 (0.0%)	3.10 (-4.3%)	2.67 (+ 21.4%)	0.55 (+ 3.8%)	0.04 (0.0%)
*60% coverage (scenarios 2 and 3) vs. 80%*	2.86 (0.0%)	3.19 (-1.5%)	2.41 (+ 9.5%)	0.55 (+ 3.8%)	0.04 (0.0%)
Vaccine unit cost 5*	1.49 (-47.9%)	1.69 (-47.8%)	1.14 (-48.2%)	0.28 (-47.2%)	0.02 (-50.0%)
Vaccine unit cost in LLMIC 5*	1.01 (-64.7%)	1.14 (-64.8%)	0.77 (-65.0%)	0.19 (-64.2%)	0.01 (-75.0%)
Vaccine unit cost 5*	0.76 (-73.4%)	0.86 (-73.5%)	0.58 (-73.6%)	0.14 (-73.6%)	0.01 (-75.0%)
Vaccine unit cost 5*	0.61 (-78.7%)	0.69 (-78.7%)	0.47 (-78.6%)	0.11 (-79.2%)	0.01 (-75.0%)
Vaccine unit cost 5*	0.51 (-82.2%)	0.58 (-82.1%)	0.39 (-82.3%)	0.09 (-83.0%)	0.01 (-75.0%)
Vaccine unit cost 5 in LLMIC*	34.76 (+ 1,115%)	41.19 (+ 1,171%)	28.57 (+ 1,199%)	7.98 (+ 1,406%)	0.58 (+ 1,350%)
*Treatment cost 60*	2.85 (-0.3%)	3.23 (-0.3%)	2.19 (-0.5%)	0.53 (0.0%)	0.04 (0.0%)
*Treatment cost 60*	3.48 (+ 21.7%)	3.87 (+ 19.4%)	3.11 (+ 41.4%)	0.75 (+ 41.5%)	0.05 (+ 25.0%)
*Engagement in care halved †*	2.72 (-4.9%)	3.04 (-6.2%)	2.04 (-7.3%)	0.49 (-7.5%)	0.03 (-25.0%)
*Engagement in care doubled †*	3.08 (+ 7.7%)	3.53 (+ 9.0%)	2.43 (+ 10.5%)	0.59 (+ 11.3%)	0.04 (0.0%)
*Employment rate for people who inject drugs 0%* ‡	1.19 (-58.4%)	1.46 (-54.9%)	1.04 (-52.7%)	0.26 (-50.9%)	0.02 (-50.0%)
*Employment rate for people who inject drugs 50% lower than the general population.* ‡	2.02 (-29.4%)	2.35 (-27.5%)	1.62 (-26.4%)	0.40 (-24.5%)	0.03 (-25.0%)

Abbreviations: LLMIC: Low and Lower-Middle Income Countries; HIC: High Income Countries.

* Vaccine costs maintained the unit cost ratio of 1:2:15 in low and lower-middle, upper-middle and high income settings.

† Engagement in care varies according to disease stage and income status. See methodology for more detail.

*‡ Employment rate for people who inject drugs is assumed equal to that of the general population in the baseline. The rate is different for each setting*

#### Intervention costs

Hepatitis C diagnostic tests, treatments, and vaccines were costed using an ingredients-based approach, comprised of a commodity and service component. Commodity costs were sourced from the Clinton Health Access Initiative 2023 Hepatitis C Market Intelligence Report [32], and delivery assumptions were adapted from previously published approaches [33].

Service delivery costs were estimated using salaries equivalent to employment-adjusted Gross Domestic Product (GDP) per capita, and included staff time per test, treatment or vaccination. We assumed hepatitis C antibody testing to primarily (90%) use rapid diagnostic tests over laboratory-based assays and conservatively assumed negotiated ceiling price for DAAs (US$60). Vaccine procurement costs were modelled at US$5 per dose in low and lower-middle income countries, US$10 per dose in upper-middle income countries, and US$75 in high income countries (tested in sensitivity analysis), consistent with price differences observed in vaccines used in broadly comparable target populations [34, 35].

All costs included provision for wastage, logistics, infrastructure, and program delivery.

#### Disease management costs

To estimate disease management costs (i.e. medical appointments and treatments for hepatitis C-associated liver disease sequelae), we assumed that during the asymptomatic stages of disease (F0–F4), only diagnosed individuals would require care, while symptomatic illness (DC and HCC) would require care regardless of disease awareness [36]. Imperfect accessibility and adherence to care varied by country income classification level and hepatitis C disease state.

#### Productivity costs

Productivity losses from hepatitis C were calculated using a human capital approach, where productivity was lost due to illness (presenteeism and absenteeism) and hepatitis C-attributable deaths. Employment was assumed to be zero among people in prison, equal among people who inject drugs and the general population aged 18–64, and to cease at age 65.

#### Benefit-cost ratio

The main outcome was the benefit-cost ratio, defined as the ratio of economic benefits to total cost for each scenario. The total cost represented the sum of commodity and human resource expenditures on vaccination, while economic benefits were calculated as the difference in expenditure on testing, disease management, treatment and productivity losses between a scenario and its counterfactual. Disability-adjusted life years (DALYs) are reported as a health impact measure and are not assigned a monetary value in the benefit-cost analysis; their economic equivalent is implicitly captured through the productivity cost component. A benefit-cost ratio greater than 1 indicates that investment in the vaccine generates a net economic gain.

#### Sub-analysis: cost-utility analysis

To allow cross-sectorial comparability of results, we estimated incremental cost-effectiveness ratios (ICERs) as the incremental cost to avert a DALY. ICERs only considered direct health costs (vaccination, diagnosis, treatment, and disease management), and were estimated for each scenario against its counterfactual. As only direct health costs are included, the cost-utility analysis adopts a healthcare system perspective rather than the broader societal perspective used for the benefit-cost analysis. DALYs were calculated as the sum of years of life lost from hepatitis C mortality and years lost to disability from decompensated cirrhosis and hepatocellular carcinoma, discounted at 3% per annum.

#### Economic outcomes

Outcomes were aggregated and reported at the global level, by WHO region, and for the ten countries with the highest estimated hepatitis C burden (Pakistan, India, China, Russia, the United States of America, Indonesia, Nigeria, Ukraine, Uzbekistan and Bangladesh [3]).

### Univariate sensitivity analysis

We sought to examine the impact that varying vaccine assumption parameters, coverage of interventions and cost estimates had on the benefit-cost ratios globally. Table 1 describes the sensitivity analyses conducted.

## Results

### Model calibration and validation

Model calibrations to national prevalence, incidence, treatment numbers and hepatitis C-related mortality (aggregated across all population groups) are provided for all countries in Supplement Section C, Table 5.

**Fig. 2:**
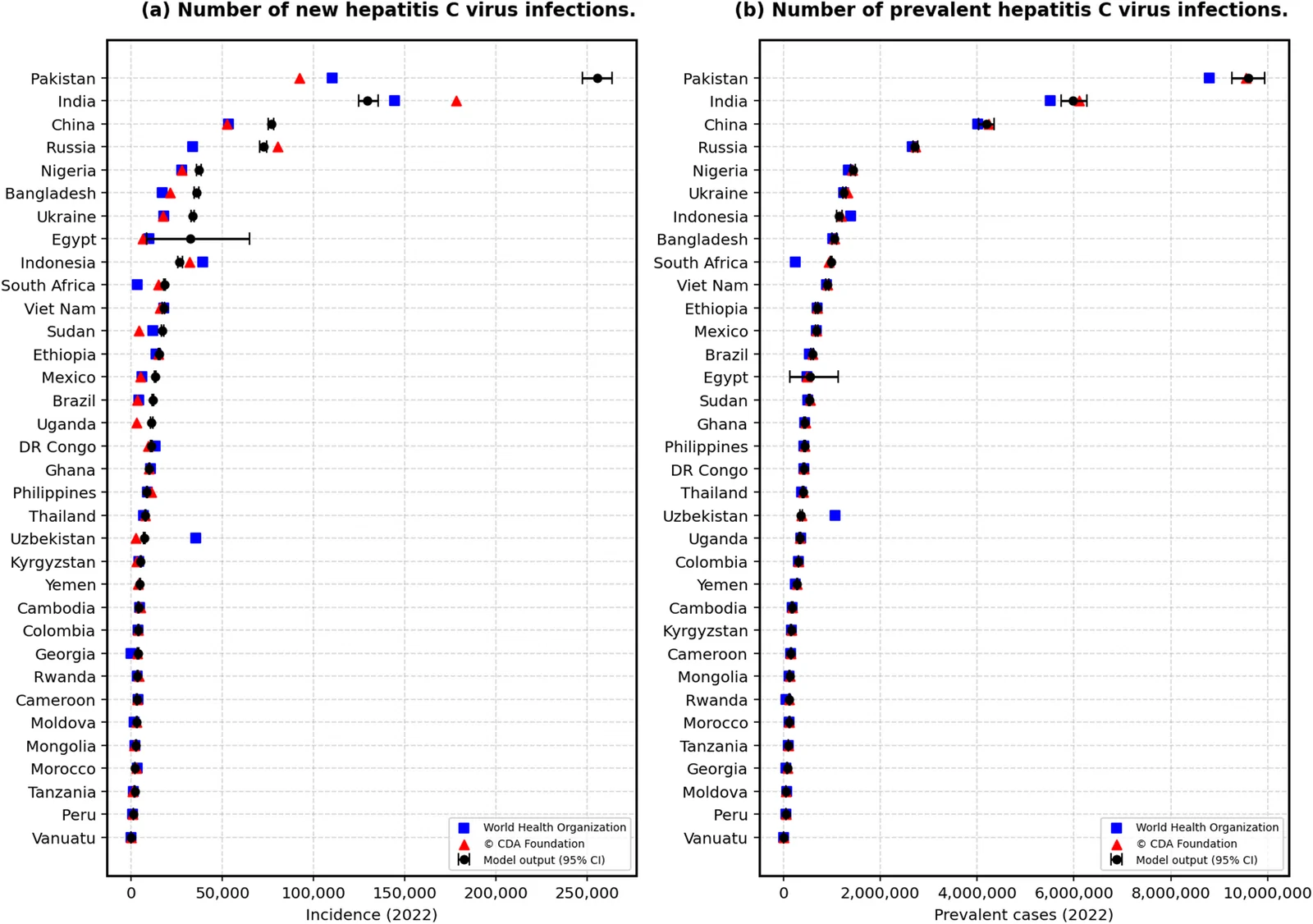
Comparison of prevalence and incidence estimates in 2022 by country. The black circles with error bars represent our model outputs and 95% confidence interval, the red triangles represent the 2024 Global Hepatitis Report [3] and the blue squares the Polaris Observatory estimates [28]. (**a**) Number of new hepatitis C virus infections by country; (**b**) Prevalent hepatitis C virus infections by country

For countries with existing hepatitis C burden estimates from the WHO’s Global Hepatitis Report 2024 [3], we validated model outputs by comparing prevalence and incidence with both this report and estimates from the Polaris Observatory [28]. Since the latter reports the number of new adult and paediatric chronic infections, we adjusted their adult incidence estimates by the spontaneous clearance rate to ensure comparability. These comparisons can be seen on Fig. 2. Supplement Section C provides these comparisons in a table. Overall, the models produced reasonably consistent outputs, with the exception of prevalent infections in some high-burden countries.

### Global outcomes

Globally, between 2026 and 2050, vaccinating people who inject drugs at the rate of existing testing (Scenario 1) was estimated to avert 1.13 million (95% CI: 825 thousand to 1.42 million) hepatitis C infections, 1.41 thousand (95% CI: 562 to 2.12 thousand) cases of hepatocellular carcinoma, 2.49 (95% CI: 1.00 to 3.69) thousand hepatitis C-related deaths and 13.7 (95% CI: 5.62 to 20.9) thousand DALYs compared with the status-quo. Vaccination-related costs were estimated to be $985 million (95% CI: $857 million to $1.08 billion), while benefits due to saved health care costs and productivity gains were estimated to be $2.81 (95% CI: $2.10 to $3.67) billion. This scenario yielded an ICER of $51.5 (95% CI: $24.9 to $126) thousand, and a benefit-cost ratio of 2.86 (95% CI: 2.20 to 3.76).

Scenario 2 was estimated to avert 1.58 (95% CI: 1.38 to 1.80) million infections, 3.67 (95% CI: 1.50 to 5.06) thousand cases of hepatocellular carcinoma, 5.66 (95% CI: 2.26 to 7.89) thousand deaths and 35.3 (95% CI: 14.2 to 51.3) thousand DALYs compared with its counterfactual. Vaccination-related costs were estimated to be $1.15 (95% CI: $1.03 to $1.23) billion, saving $3.71 (95% CI: $3.05 to $4.53) billion in healthcare expenditure and productivity gains. This scenario yielded an ICER of $19.1 (95% CI: $6.93 to $48.6) thousand and a benefit-cost ratio of 3.24 (95% CI: 2.68 to 4.06).

Scenario 3 was estimated to avert 2.36 (95% CI: 2.25 to 2.49) million infections, 4.5 (95% CI: 1.81 to 6.07) thousand cases of hepatocellular carcinoma, 7.4 (95% CI: 2.92 to 10.0) thousand hepatitis C-related deaths and 46.2 (95% CI: 18.2 to 66.2) thousand DALYs compared to its counterfactual. Vaccination-related costs were estimated to be $1.90 (95% CI: $1.83 to $1.96) billion, saving $4.19 (95% CI: $3.60 to $4.95) billion in healthcare expenditure and productivity gains. The ICER for scenario 3 was $27.3 (95% CI: $13.4 to $72.7) thousand, and the benefit-cost ratio was 2.20 (95% CI: 1.88 to 2.63).

Scenarios 4 and 5, in which vaccination was introduced in schools on a rolling basis from 2026 to 2050 and among all adults aged 18–64 years between 2026 and 2031, had ICERs of $154 (95% CI: $104 to $384) thousand and $1.61 (95% CI: $1.05 to $4.50) million respectively, compared to their counterfactuals, and benefit-cost ratios of 0.53 (95% CI: 0.46 to 0.63) and 0.04 (95% CI: 0.03 to 0.04).

**Fig. 3:**
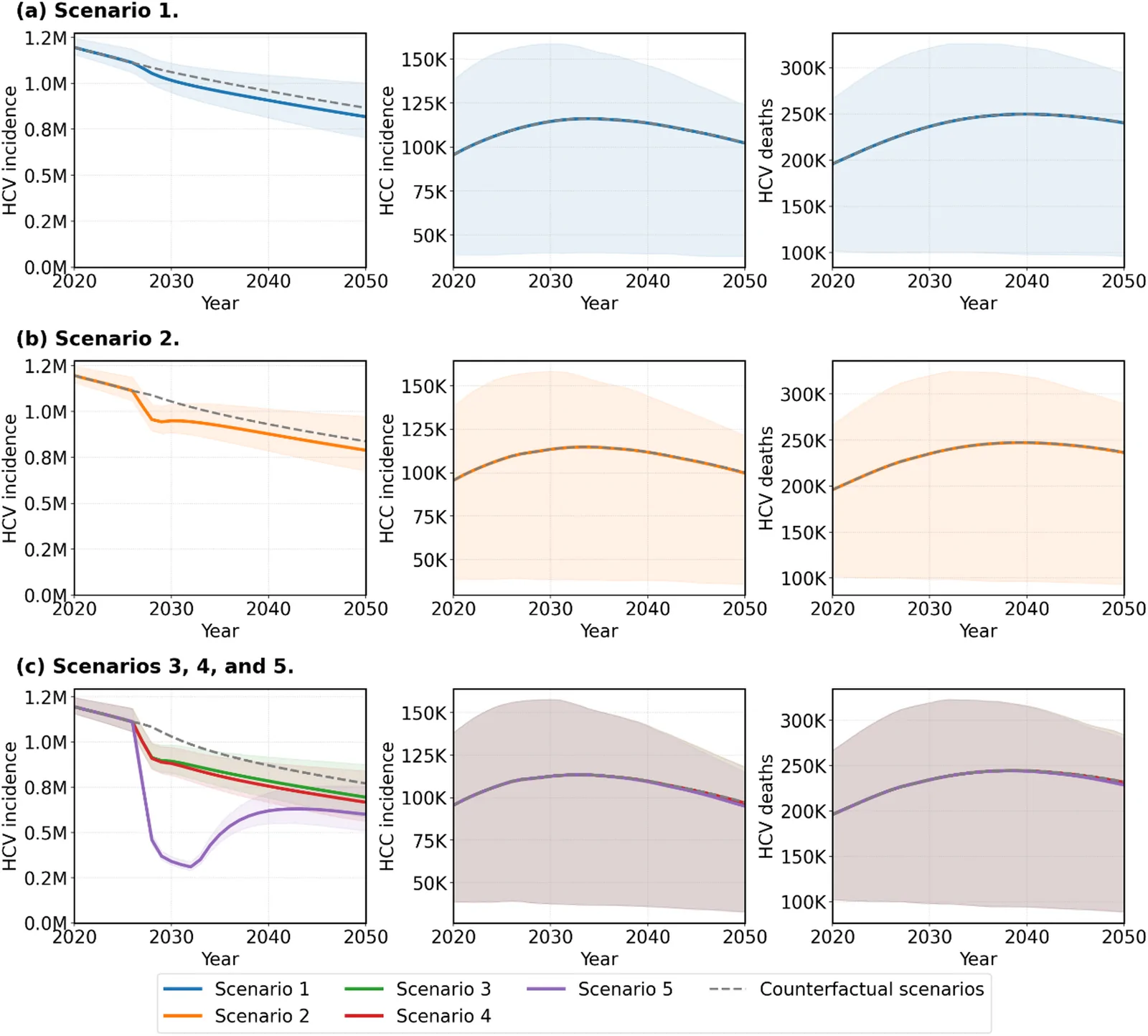
Time series of the number of new hepatitis C virus (HCV) infections, cases of hepatocellular carcinoma (HCC), and HCV-related deaths across 116 countries. (**a**) Scenario 1; (**b**) Scenario 2; (**c**) Scenarios 3, 4 and 5. Dashed lines represent the counterfactuals, coloured lines the scenario projections, and the shaded areas the 95% confidence intervals

Table 2 summarises the vaccine-attributable outcomes (e.g. differences between a scenario and its counterfactual) on a global scale, and Fig. 3 time-varying plots of the number of new infections, hepatocellular carcinoma cases, and hepatitis C-related deaths. Detailed vaccine-attributable outcomes by region can be found in Supplement Section D, Table 6.

### Regional benefit-cost ratios

Table 3 presents benefit-cost ratios for the five vaccination scenarios between 2026-2050. Vaccination of people who inject drugs delivered through existing testing services, via a rapid scale-up, and in prison settings (Scenarios 1, 2 and 3) was estimated to have benefit-cost ratios greater than 1 in all regions. Vaccination of adolescents and adults (Scenario 4 and 5) was estimated to have benefit-cost ratios below one in all regions. Country-level benefit-cost ratios are presented in Supplement Section E, Table 7.

### Sensitivity analyses

Sensitivity analyses, evaluated on the central results only, showed that benefit-cost ratios were most sensitive to vaccine efficacy, duration of protection, and vaccine unit cost (Table 4). Reducing the spontaneous clearance rate to 50% among vaccinated individuals (vs. 80%) decreased the benefit-cost ratios by 50.0–55.2% across scenarios (Scenario 1: 1.30 vs. 2.86; Scenario 2: 1.45 vs. 3.24; Scenario 3: 1.01 vs. 2.20), whereas a spontaneous clearance rate of 100% increased the benefit-cost ratios by 25.0–37.0% (Scenario 1: 3.86 vs. 2.86; Scenario 2: 4.44 vs. 3.24; Scenario 3: 2.97 vs. 2.20). Weighted efficacy across genotypes, assuming 100% efficacy for one genotype and 0% for others, lowered benefit-cost ratios by 30.8–100.0%, resulting in benefit-cost ratios greater than 1 only for genotype 1 (Scenario 1: 1.98 vs. 2.86; Scenario 2: 2.17 vs. 3.24; Scenario 3: 1.49 vs. 2.20). A trivalent genotype-specific vaccine, covering the three most prevalent genotypes globally, increased the benefit-cost ratios by 0.0% (Scenario 5)–11.5% (Scenario 1).

When duration of protection increased to 30 years or more, adding school-based vaccinations (Scenario 4) achieved a benefit-cost ratio greater than 1 globally. Lifelong protection increased the benefit-cost ratio by 125.0–189.5% across scenarios (Scenario 1: 7.13 vs. 2.86; Scenario 2: 8.49 vs. 3.24; Scenario 3: 6.37 vs. 2.20; Scenario 4: 1.27 vs. 0.53). On the other hand, a shorter duration of protection of 1 year resulted in benefit-cost ratios less than 1 globally across scenarios.

Lowering the cost of DAA treatment by 50% (to $30 in all setting), or increasing it to $5,000 in high-income countries, did not substantially impact global benefit-cost ratios: Scenarios 1 to 3 achieved benefit-cost ratios between 2 and 4, while the benefit-cost ratios of Scenarios 4 and 5 remained below 1.

When vaccine unit cost exceeded $15 in low- and lower-middle income countries (maintaining the unit cost ratio of 1:2:15 in low and lower-middle, upper-middle and high income settings), all scenarios resulted in benefit-cost ratios below 1. At an acquisition cost of $0, retaining delivery costs only, the benefit-cost ratio for Scenario 4 increased to 7.98, representing a 1,406% improvement over the base case, whereas Scenario 5 still did not achieve a benefit-cost ratio greater than 1 globally.

Assuming 0% employment among people who inject drugs reduced benefit-cost ratios by approximately 50.0–58.4% across scenarios, while assuming a 50% lower employment rate reduced benefit-cost ratios by approximately 24.5–29.4%. Under both assumptions, the relative ranking of scenarios was unchanged and Scenarios 1–3 retained benefit-cost ratios greater than 1, though Scenarios 4 and 5 remained below 1.

## Discussion

This study found that in the context of heterogeneous treatment scale-up among countries and population groups, a hepatitis C vaccine is likely to have a positive return-on-investment when used to vaccinate populations at highest risk of infection. For example, we estimate that globally, for every dollar spent on vaccinating people who inject drugs within existing testing and treatment services, $2.86 (95% CI: $2.20 to $3.76) could be saved in reduced healthcare costs and productivity losses. In contrast, unless a vaccine has a long duration of protection, vaccinating adolescents in schools is unlikely to yield a positive return, and while vaccinating all adults aged 18–64 years could produce substantial reductions in infections, hepatocellular carcinoma cases and hepatitis C-related deaths, it would have a very high cost and hence be unlikely to yield a positive return.

These findings have significant implications for the future development and trialling of hepatitis C vaccines. The results suggest that unless a future vaccine can achieve a long duration of protection, the predominant (and for many countries the only) target population is going to be people who inject drugs. Vaccine trials should therefore include people who inject drugs and consider acceptability given they are likely to be the primary vaccine recipients.

Challenges scaling up testing and treatment interventions among people who inject drugs would also apply to vaccine delivery. For example, additional interventions may be required to retain people in care if vaccine candidates required multiple doses or complex delivery, or if RNA-negative confirmation and vaccination required separate appointments. Achieving high coverage of a vaccine program among people who inject drugs may also entail substantial service delivery challenges. In many settings, effective engagement of this population requires community-based or mobile outreach, peer navigation, and integration with harm reduction services such as needle and syringe programs. Our service delivery costs are estimated using an ingredient-based approach and reflect a standard clinical delivery model where vaccination is added onto an existing test-treat program, which may be appropriate for Scenario 2 (assuming vaccination only achieves current testing coverage), but to achieve high coverage these do not account for the additional outreach intensity or programme infrastructure required to retain marginalised populations across multiple encounters. Real-world implementation costs may be higher than estimated, particularly in settings where harm reduction infrastructure is limited, and the benefit-cost ratios reported here should be interpreted accordingly.

This study also explores how the value proposition of a hepatitis C vaccine would change if countries significantly increased their efforts to scale-up testing and treatment. For example, Scenario 3 considers the incremental impact of adding vaccination to a situation where every country has implemented a one-off test-treat program reaching 80% of people who inject drugs and is testing and treating 80% of prisoners on an ongoing basis. In this example, the vaccine still had good value, with vaccine-attributable impact having a benefit-cost ratio of 2.20 (95% CI: 1.88 to 2.63). Sensitivity analyses with lower coverage of test-treat programs in Scenario 3 showed similar results, supporting the notion that regardless of how countries continue to implement their elimination programs, in the absence of major changes to prevention interventions a vaccine is likely to have a positive return-on-investment.

For comparison against other health metrics, we also estimated the incremental cost per DALY averted for the addition of vaccines to different test-treat programs. Globally, Scenario 2 achieved the lowest ICER ($19 thousand), followed by Scenario 3 ($27 thousand), Scenario 1 ($51 thousand), and Scenarios 4 and 5 ($154 thousand and $1.6 million, respectively). While vaccination reduced the incidence of new infections, most of the reductions in morbidity and mortality were attributed to the scale-up of treatment. This is because hepatitis C-related liver disease develops slowly over decades, and treatment is 95% curative, preventing progression among people already infected. To support interpretation, we compared the ICERs with GDP per capita across countries, as recommended by the WHO-CHOICE. Under Scenarios 1 and 2, the vaccine was cost-effective (≤ 1 × GDP per capita) in 43% and 56% of countries, respectively, and highly cost-effective (≤ 0.5 × GDP per capita) in 34% and 44%. Scenario 3 showed moderate cost-effectiveness (44% of countries ≤ 1 × GDP per capita) while Scenarios 4 and 5 were cost-effective in only 10% and 3% of countries, respectively.

It is possible that a future hepatitis C vaccine may only be effective against some genotypes, and sensitivity analyses indicated that this would heavily impact the benefit-cost ratios. A monovalent vaccine was not likely to have a global benefit-cost ratio > 1 unless it was either effective against genotype 1, which accounts for 49% of global hepatitis C infections [11], or strategically targeted to settings where the specific genotypes were high prevalence, limiting potential scale. However, a trivalent vaccine covering more than 80% of genotypes in circulation globally would achieve benefit-cost ratios greater than the baseline for Scenarios 1 to 4.

When the unit cost was greater than or equal to $20 in low- and lower-middle income countries (equivalent to $40 in upper-middle income countries and $300 in high-income countries), costs outweighed benefits globally. Notably, even at a vaccine acquisition cost of $0, mass adult vaccination (Scenario 5) did not achieve a benefit-cost ratio greater than 1 globally, suggesting that its limited value is driven by diminishing marginal returns under the DAA backdrop. In contrast, adolescent school-based vaccination (Scenario 4) achieved a benefit-cost ratio of 7.98 at $0 acquisition cost, indicating that low-cost procurement could substantially strengthen its economic case.

Additionally, although past infection is known to reduce vaccine efficacy in some diseases [37], our sensitivity analyses indicated that this factor has minimal influence on the benefit-cost ratios.

To explore heterogeneity in vaccine value across settings, we examined correlations between country-level benefit-cost ratios and selected epidemiological and economic indicators (Supplement Section E, Table 8). Across Scenarios 1 to 4, higher GDP per capita and higher hepatitis C RNA prevalence among people who inject drugs were associated with higher benefit-cost ratios. Existing testing coverage among people who inject drugs was also positively associated with benefit-cost ratios. Together, these findings suggest that hepatitis C vaccination yields greater economic returns in settings with more concentrated transmission and higher downstream healthcare and productivity losses, and that variation in benefit-cost ratios is primarily driven by structural differences between countries rather than by programmatic indicators that are more variably measured across settings. In contrast, these associations were not observed for Scenario 5, where broad adult vaccination produced low benefit-cost ratios across most settings irrespective of country characteristics.

Overall, our analysis suggests that for benefits to exceed costs at the global level, a future hepatitis C vaccine would need to meet several requirements. At a minimum, vaccine efficacy should be ≥ 80% against Genotype 1, the most common genotype globally, and preferably extend across Genotypes 1, 3 and 4 or be pan-genotypic. The vaccine should provide at least five years of protection, and be deliverable at a unit cost below approximately $20 in low and lower-middle income countries, $40 in upper-middle income countries, and $300 in high-income countries.

This study has several limitations. First, hepatitis C vaccines are still undergoing clinical trials, so vaccine effects in the model were hypothesised given current best knowledge with different vaccine effectiveness and durations of protection tested in the sensitivity analyses. Second, our models were calibrated using publicly available global hepatitis C estimates which were not validated by country teams, and many inputs were themselves outputs of other modelling efforts (e.g. IHME). Regional average inputs were used where country-specific estimates were not available. While we validated model outcomes against estimates from the WHO, several countries were not represented. Third, the model does not account for future changes in injecting patterns or incarceration rates, whereas in practice these may change (e.g. due to the emergence of fentanyl or other drugs). If future risks for hepatitis C infection were to increase among people who inject drugs, then this would make a potential vaccine more impactful among this group. Fourth, the model focused on people who inject drugs and people in prison as the highest incidence key populations globally, but a potential vaccine may have additional benefits for other key populations not included in the analysis, such as men who have sex with men (particularly those living with HIV or engaging in higher-risk sexual practices). Fifth, in the economic analysis, human resource and commodity costs are averaged and may not reflect local variability. We also assumed that vaccine and treatment prices were constant for the duration of the projections, whereas in reality, they may change due to market dynamics or generic equivalents. This analysis excludes research and development costs and the time needed for vaccine development. Sixth, a benefit not captured in our analysis is that successful hepatitis C elimination would yield permanent, system-wide cost savings, implying that if vaccination is required to achieve elimination, the benefit–cost ratio may be considerably more favourable than our estimates suggest. Seventh, a compartmental model was used which assumes homogeneous mixing and does not fully capture geographical clustering within each country, injecting network characteristics, or individual prisons within a country’s prison system. The analysis considers broad coverage of vaccine programs and estimates average population-level effects, but if a real-world vaccine program was targeted to higher risk individuals or settings, then the impact may be greater. Eighth, the model used large age categories to align with data available across all countries, and so doesn’t fully capture the age distribution of prevalence in each country. Ninth, our model did not capture individuals co-infected with HIV or other blood-borne viruses, which could affect disease progression, vaccine efficacy and healthcare costs.

## Conclusions

This study found that a potential hepatitis C vaccine is likely to have a positive return-on-investment when used to vaccinate populations at highest risk of infection, and for every dollar invested in vaccinating people who inject drugs and people in prison $2 to $3 could be saved globally due to reduced healthcare costs and productivity gains. These findings can be used to inform likely use cases and minimal characteristics required of future vaccines.

## Supplementary Information

Below is the link to the electronic supplementary material.

Supplementary Material 1

Supplementary Material 2

## Data Availability

The datasets supporting the conclusions of this article are available in the Burnet-Modelling/hcv-vaccine repository, https://github.com/Burnet-Modelling/hcv-vaccine. Country-level inputs can be found in the databooks folder.

## Abbreviations

DAA: Direct-acting antiviral
WHO: World health organization
F0–F4: METAVIR score for liver disease
DC: Decompensated cirrhosis
HCC: Hepatocellular carcinoma
LLMIC: Low and lower-middle income countries
HIC: High income countries
DALYs: Disability-adjusted life years
CI: Confidence interval
GDP: Gross domestic product
ICER: Incremental cost effectiveness ratio
k: Thousand
M: Million
B: Billion
