# Supplementary material for: Cost-benefit analysis of a potential hepatitis C vaccine: a modelling study
Source: BMC Infect Dis. 2026 Jul 2;26:1715. doi: 10.1186/s12879-026-13858-7 (PMC13602624; doi:10.1186/s12879-026-13858-7)
Supplement: Supplementary file 1 [file 12879_2026_13858_MOESM1_ESM.pdf]

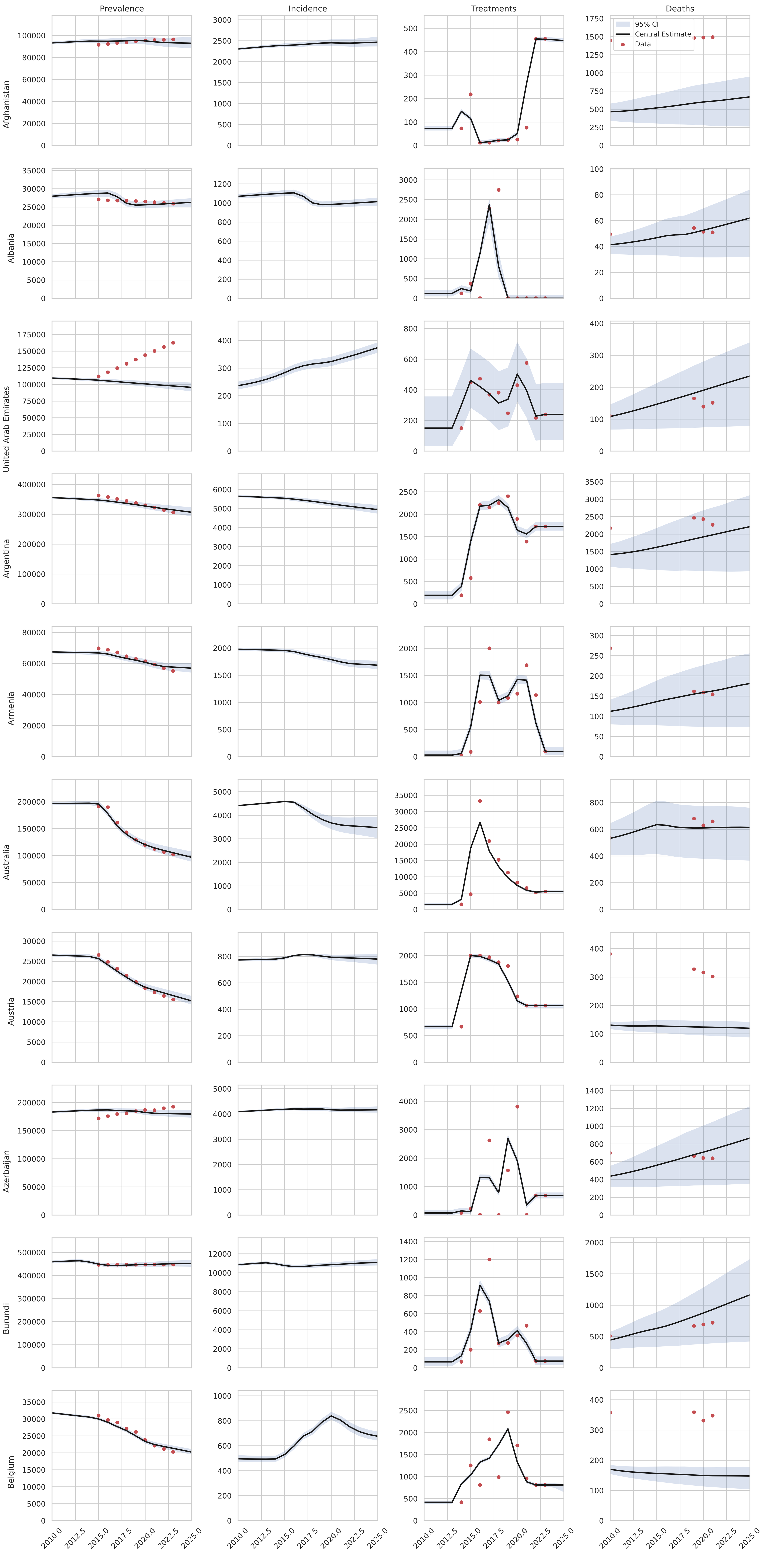

Prevalence
Incidence
Treatments
Deaths
95% CI
Central Estimate
Data
Afghanistan
Albania
United Arab Emirates
Argentina
Armenia
Australia
Austria
Azerbaijan
Burundi
Belgium

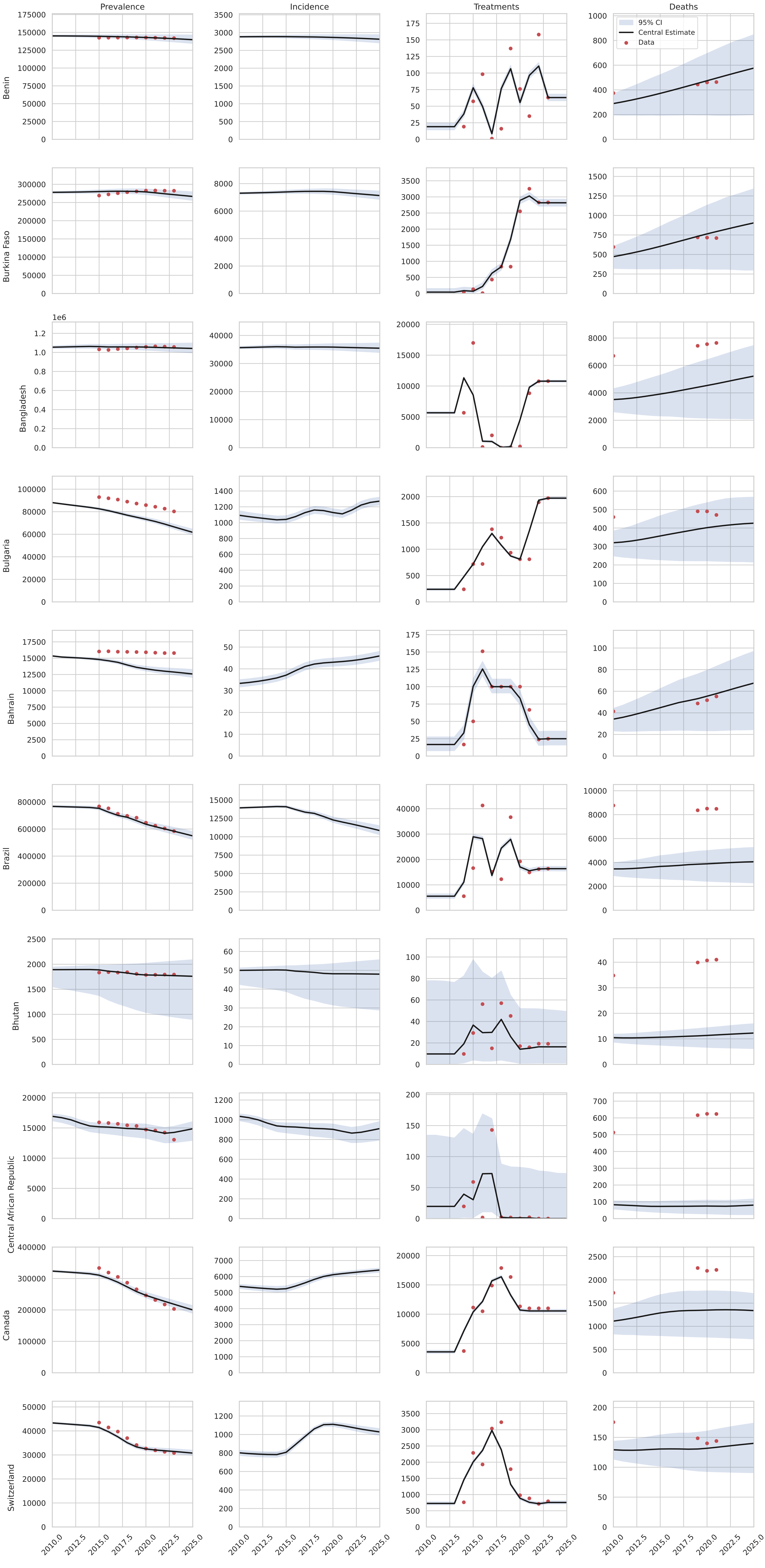

Prevalence
Incidence
Treatments
Deaths
95% CI
Central Estimate
Data
Benin
Burkina Faso
Bangladesh
Bulgaria
Bahrain
Brazil
Bhutan
Central African Republic
Canada
Switzerland
1e6

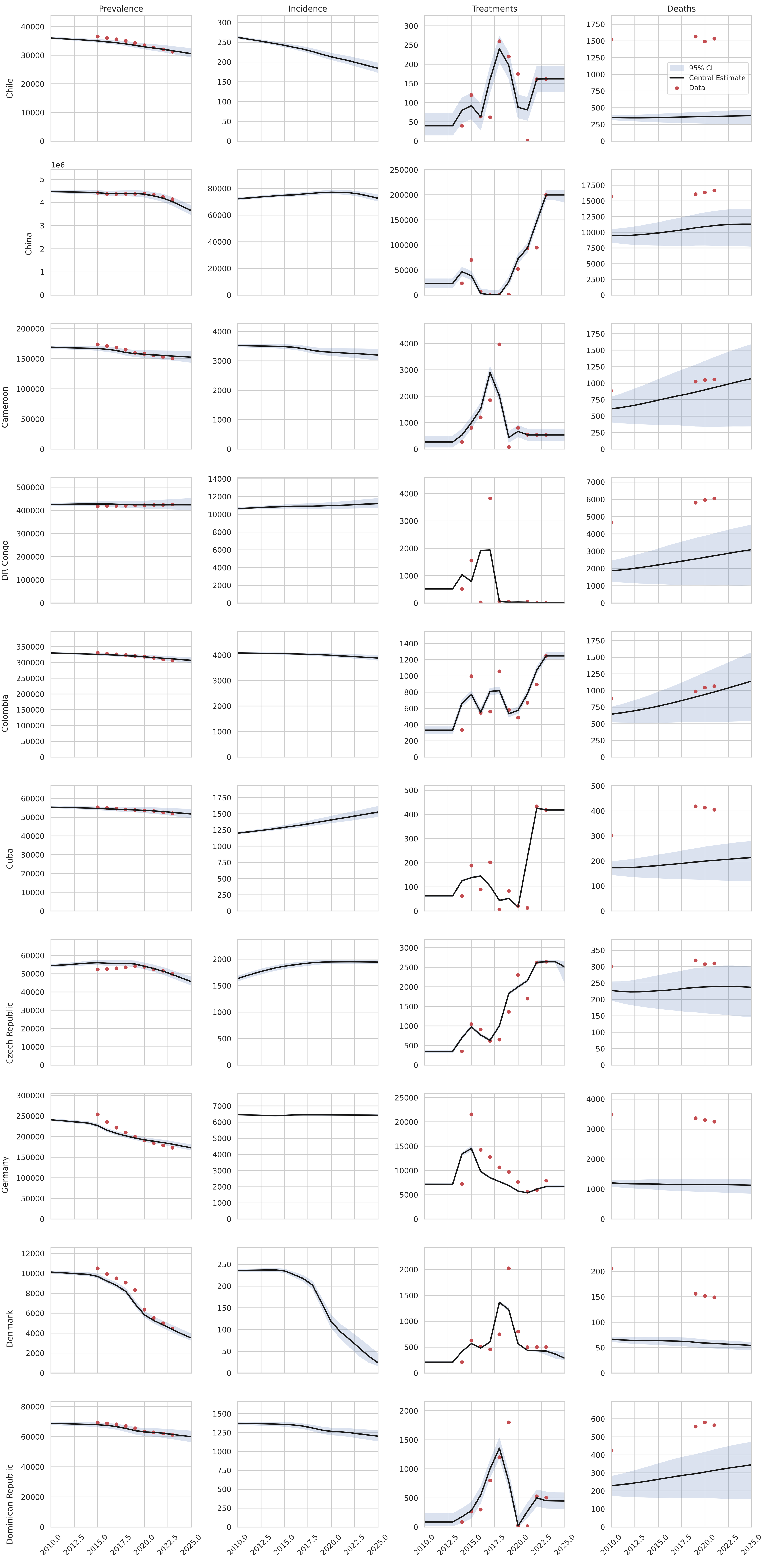

Prevalence
Incidence
Treatments
Deaths
95% CI
Central Estimate
Data
Chile
China
Cameroon
DR Congo
Colombia
Cuba
Czech Republic
Germany
Denmark
Dominican Republic
1e6

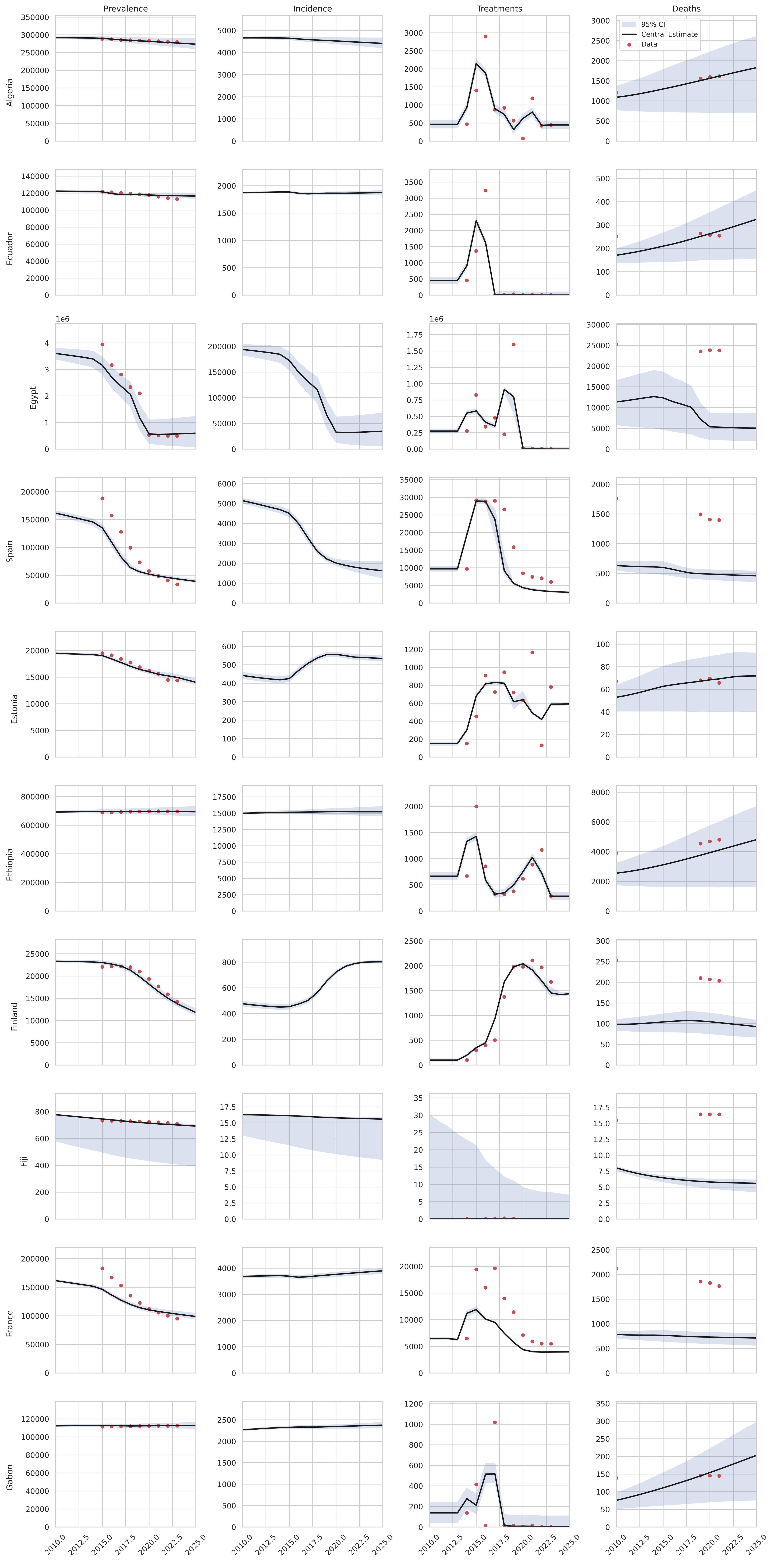

Prevalence
Incidence
Treatments
Deaths
95% CI
Central Estimate
Data
Algeria
Ecuador
Egypt
Spain
Estonia
Ethiopia
Finland
Fiji
France
Gabon
1e6

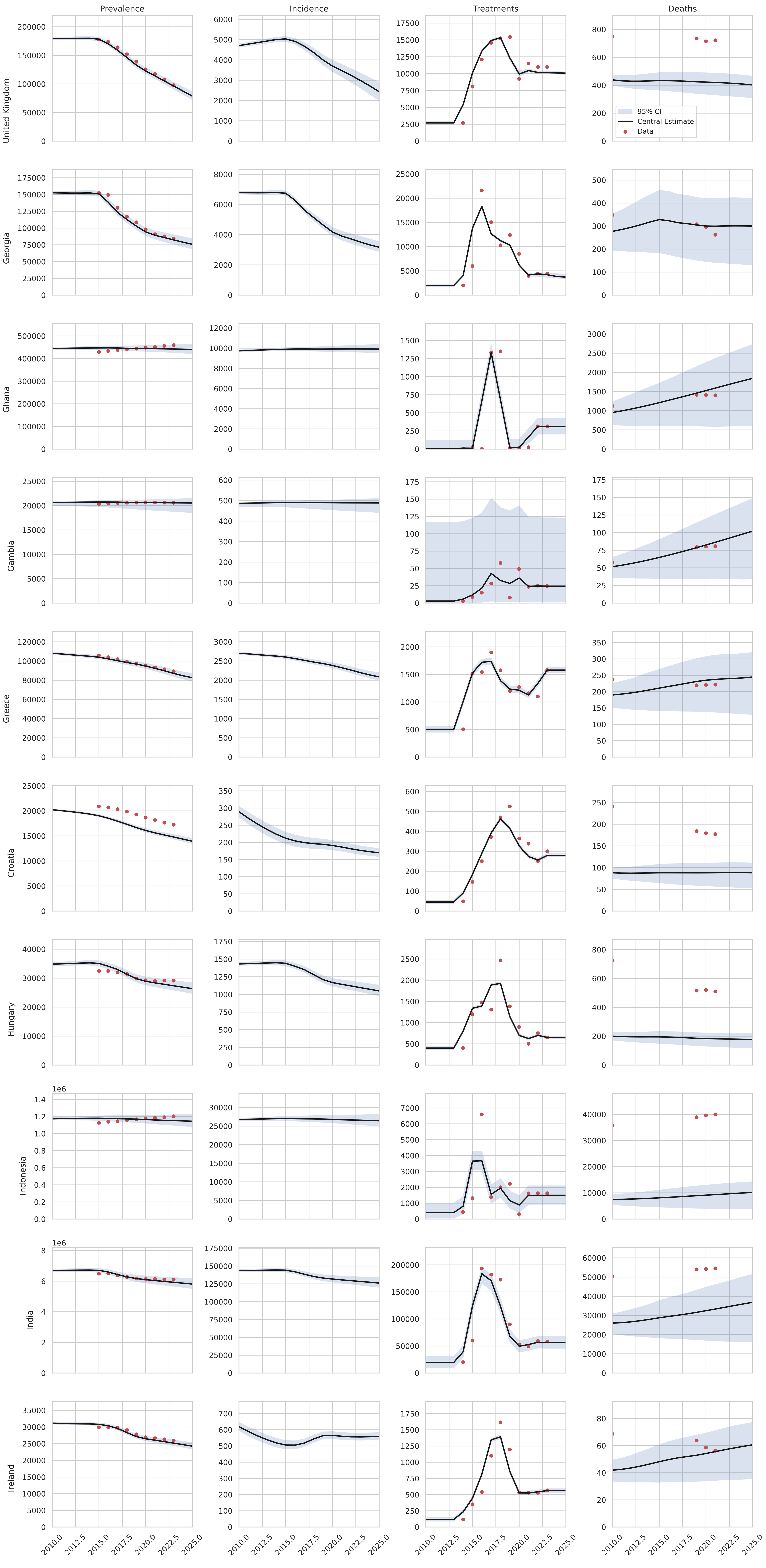

Prevalence
Incidence
Treatments
Deaths
United Kingdom
Georgia
Ghana
Gambia
Greece
Croatia
Hungary
Indonesia
India
Ireland
95% CI
Central Estimate
Data
1e6
2010.0
2012.5
2015.0
2017.5
2020.0
2022.5
2025.0

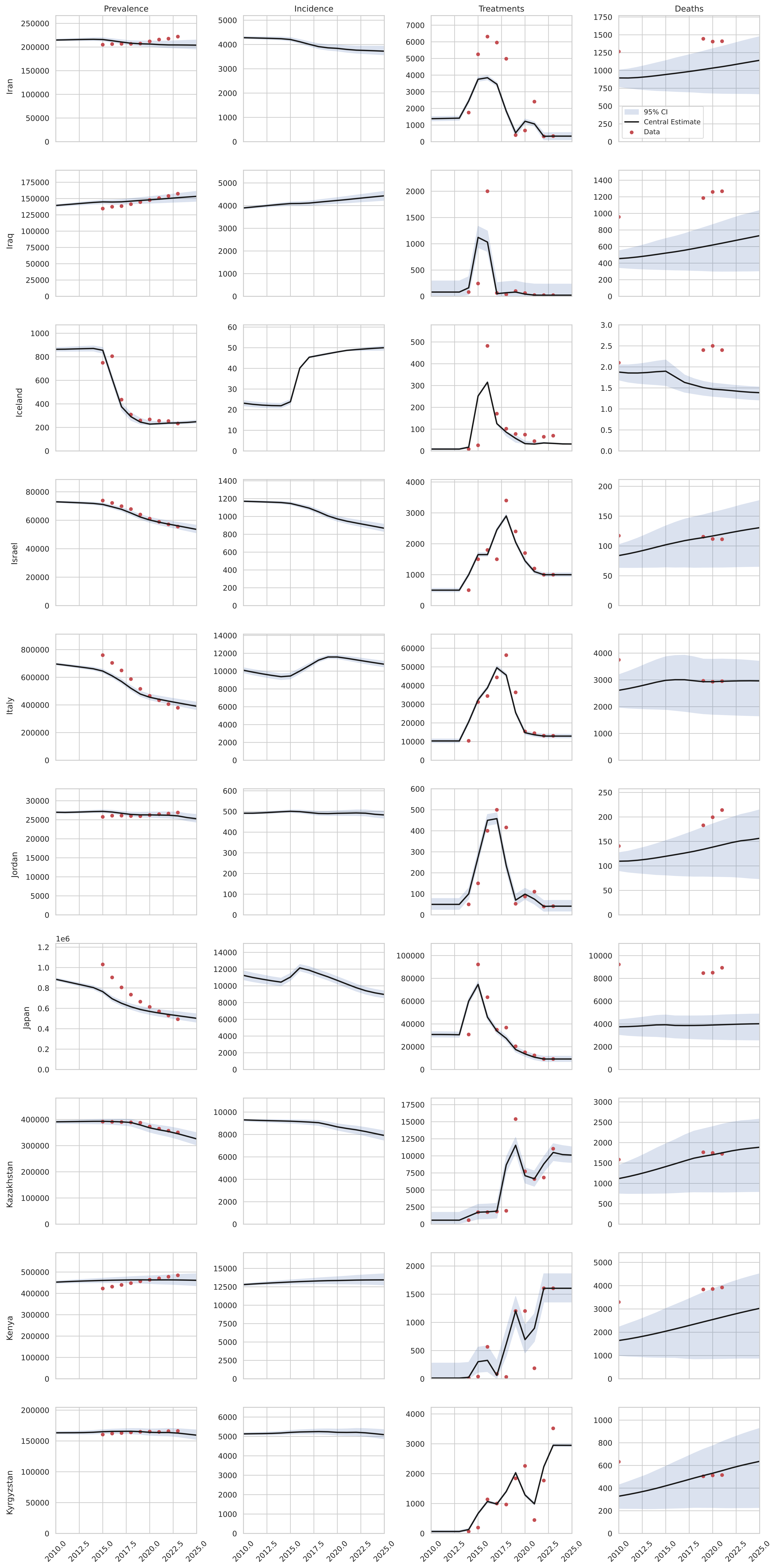

Prevalence
Incidence
Treatments
Deaths
Iran
Iraq
Iceland
Israel
Italy
Jordan
Japan
Kazakhstan
Kenya
Kyrgyzstan
95% CI
Central Estimate
Data
1e6

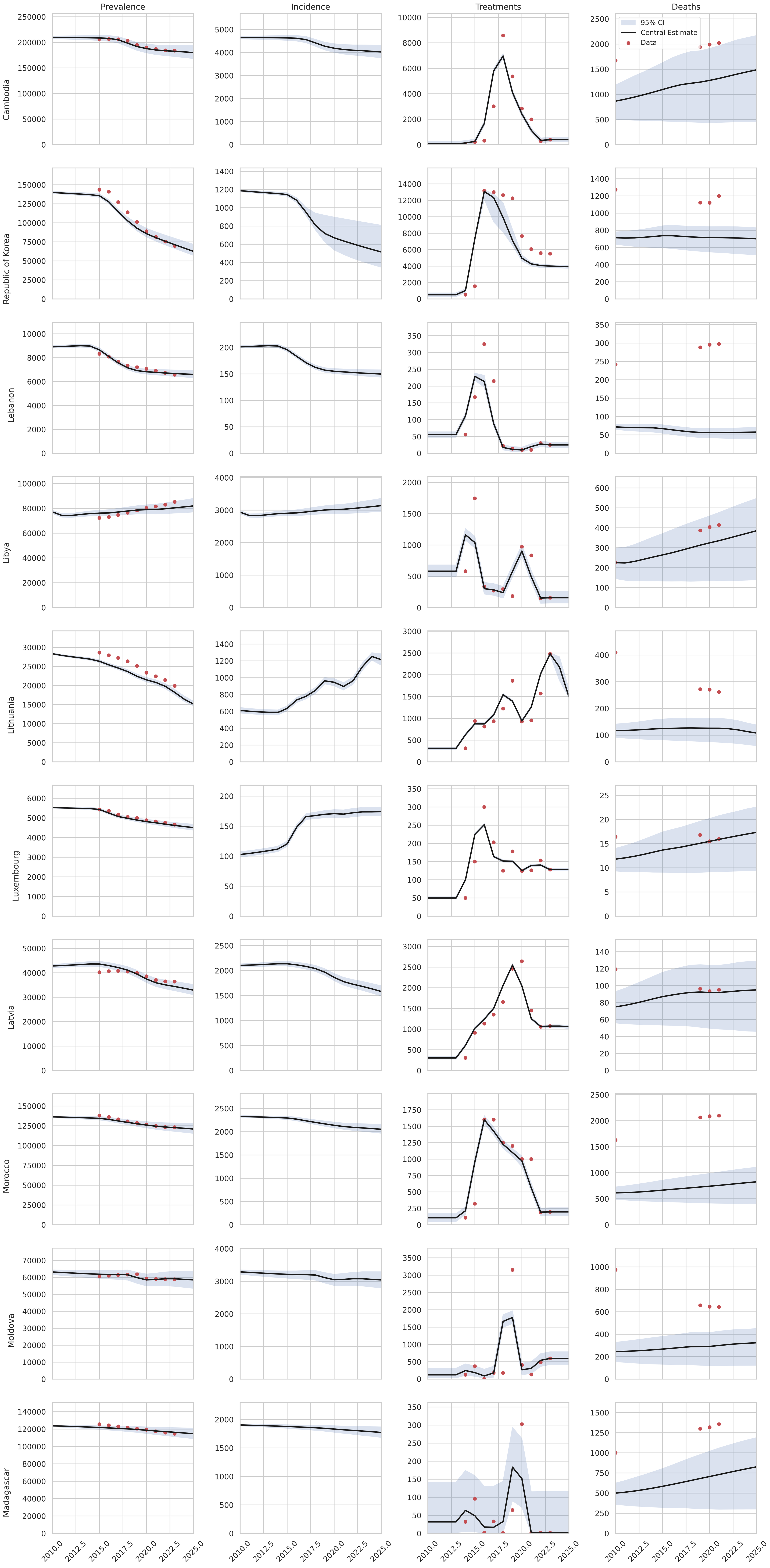

Prevalence
Incidence
Treatments
Deaths
95% CI
Central Estimate
Data
Cambodia
Republic of Korea
Lebanon
Libya
Lithuania
Luxembourg
Latvia
Morocco
Moldova
Madagascar
2010.0
2012.5
2015.0
2017.5
2020.0
2022.5
2025.0

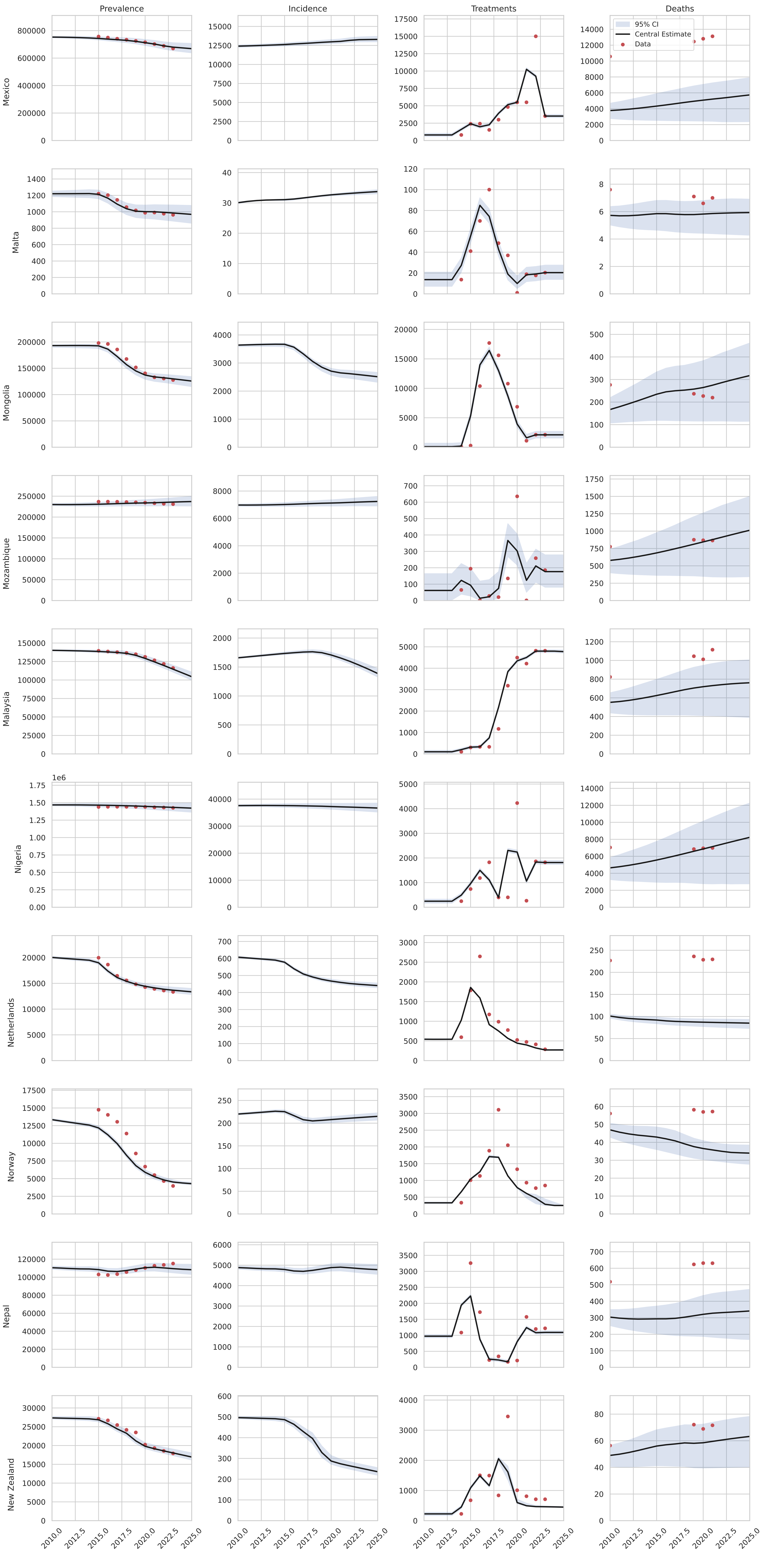

Prevalence
Incidence
Treatments
Deaths
95% CI
Central Estimate
Data
Mexico
Malta
Mongolia
Mozambique
Malaysia
Nigeria
Netherlands
Norway
Nepal
New Zealand
1e6
2010.0
2012.5
2015.0
2017.5
2020.0
2022.5
2025.0

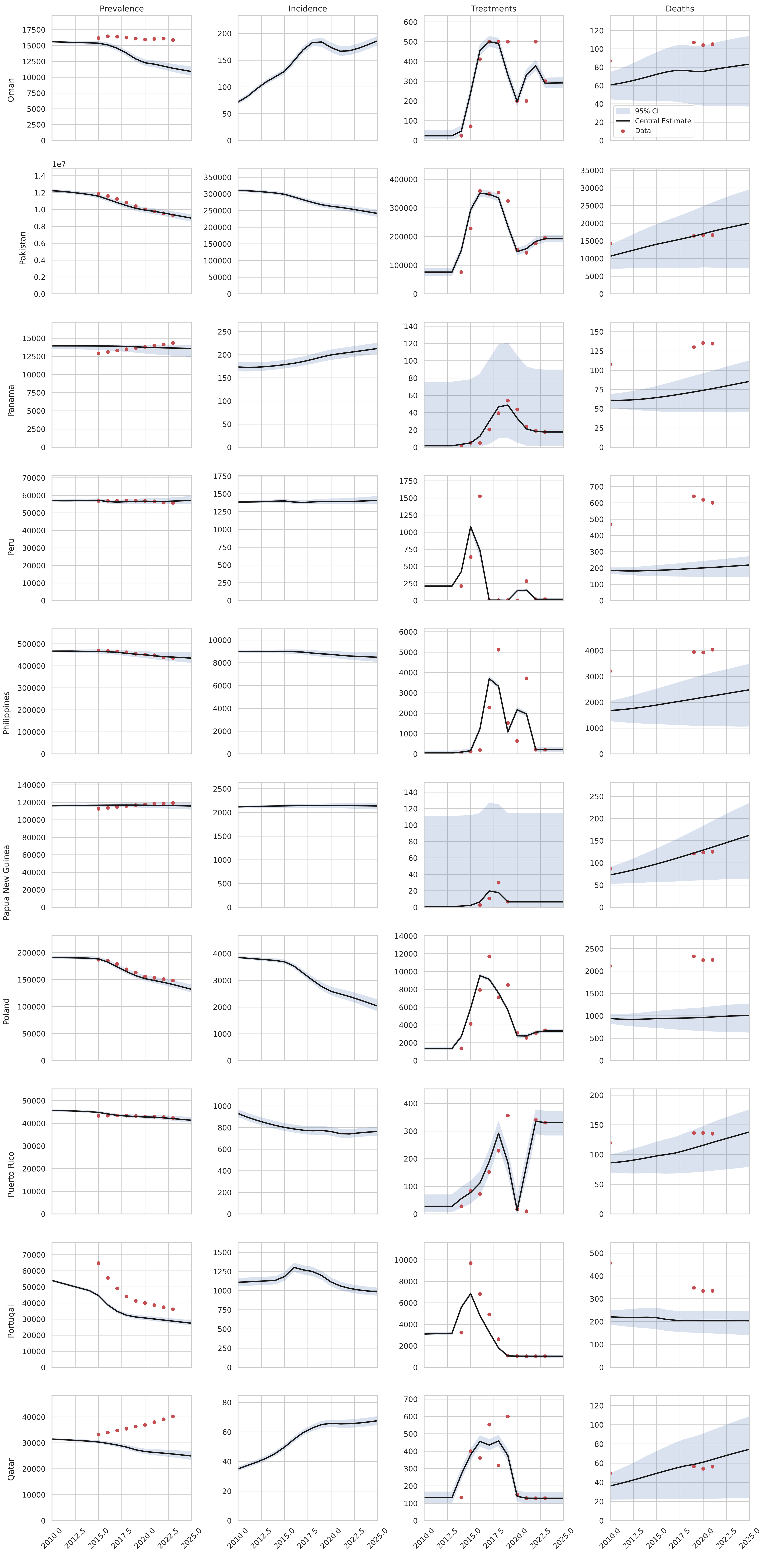

Prevalence
Incidence
Treatments
Deaths
Oman
Pakistan
Panama
Peru
Philippines
Papua New Guinea
Poland
Puerto Rico
Portugal
Qatar
95% CI
Central Estimate
Data
1e7

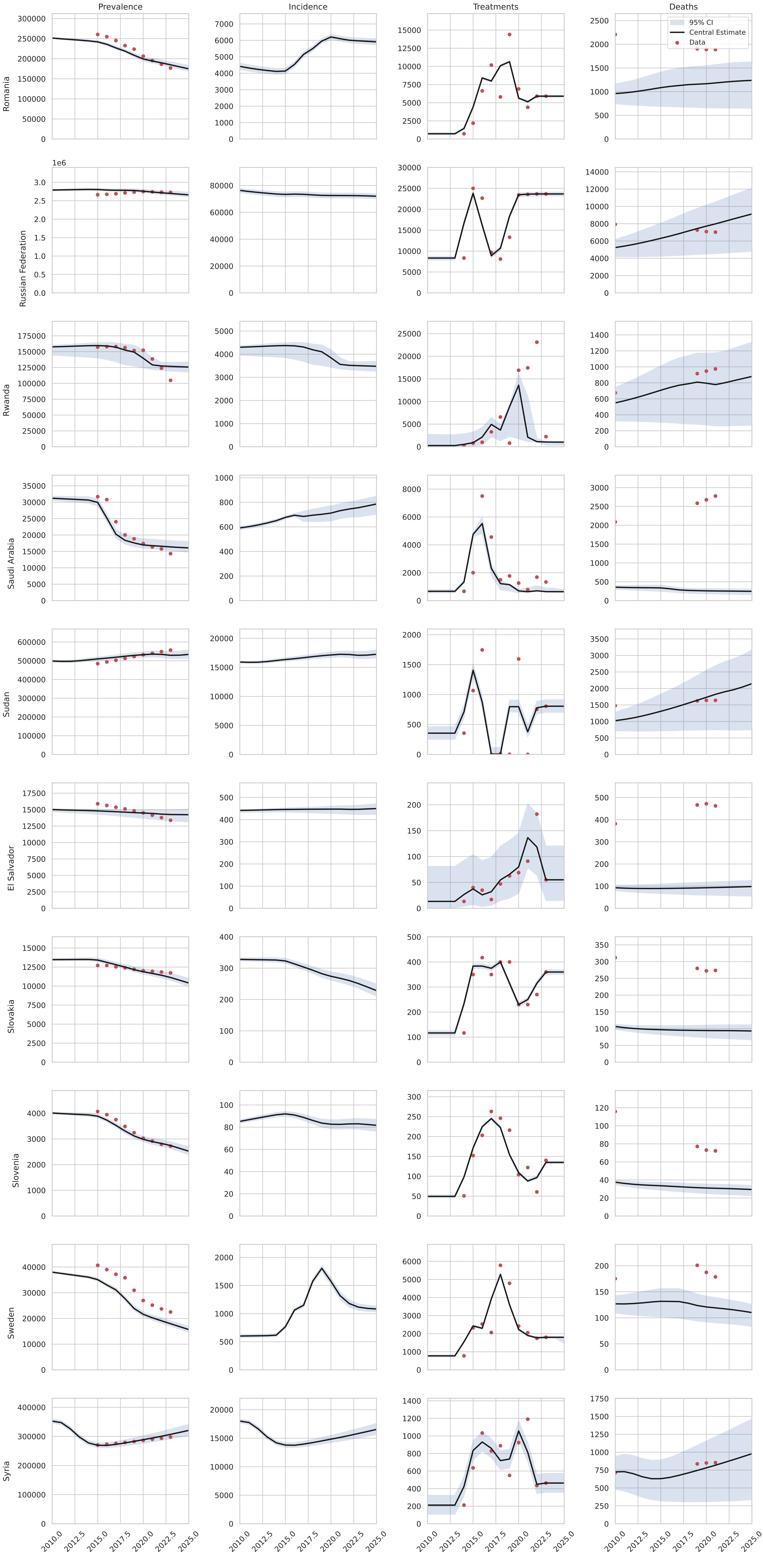

Prevalence
Incidence
Treatments
Deaths
95% CI
Central Estimate
Data
Romania
Russian Federation
Rwanda
Saudi Arabia
Sudan
El Salvador
Slovakia
Slovenia
Sweden
Syria
1e6

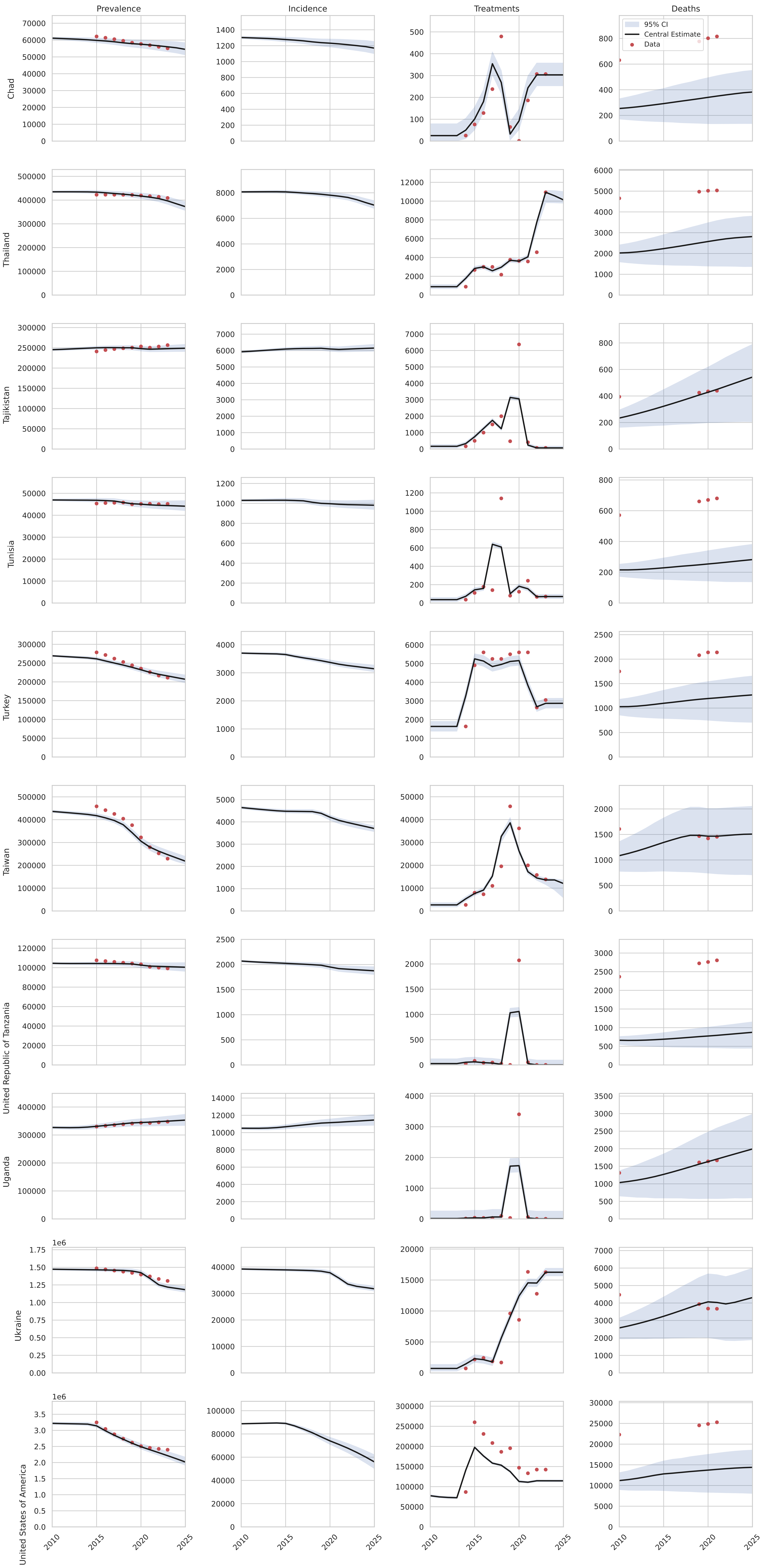

Prevalence
Incidence
Treatments
Deaths
95% CI
Central Estimate
Data
Chad
Thailand
Tajikistan
Tunisia
Turkey
Taiwan
United Republic of Tanzania
Uganda
Ukraine
United States of America
1e6
2010
2015
2020
2025

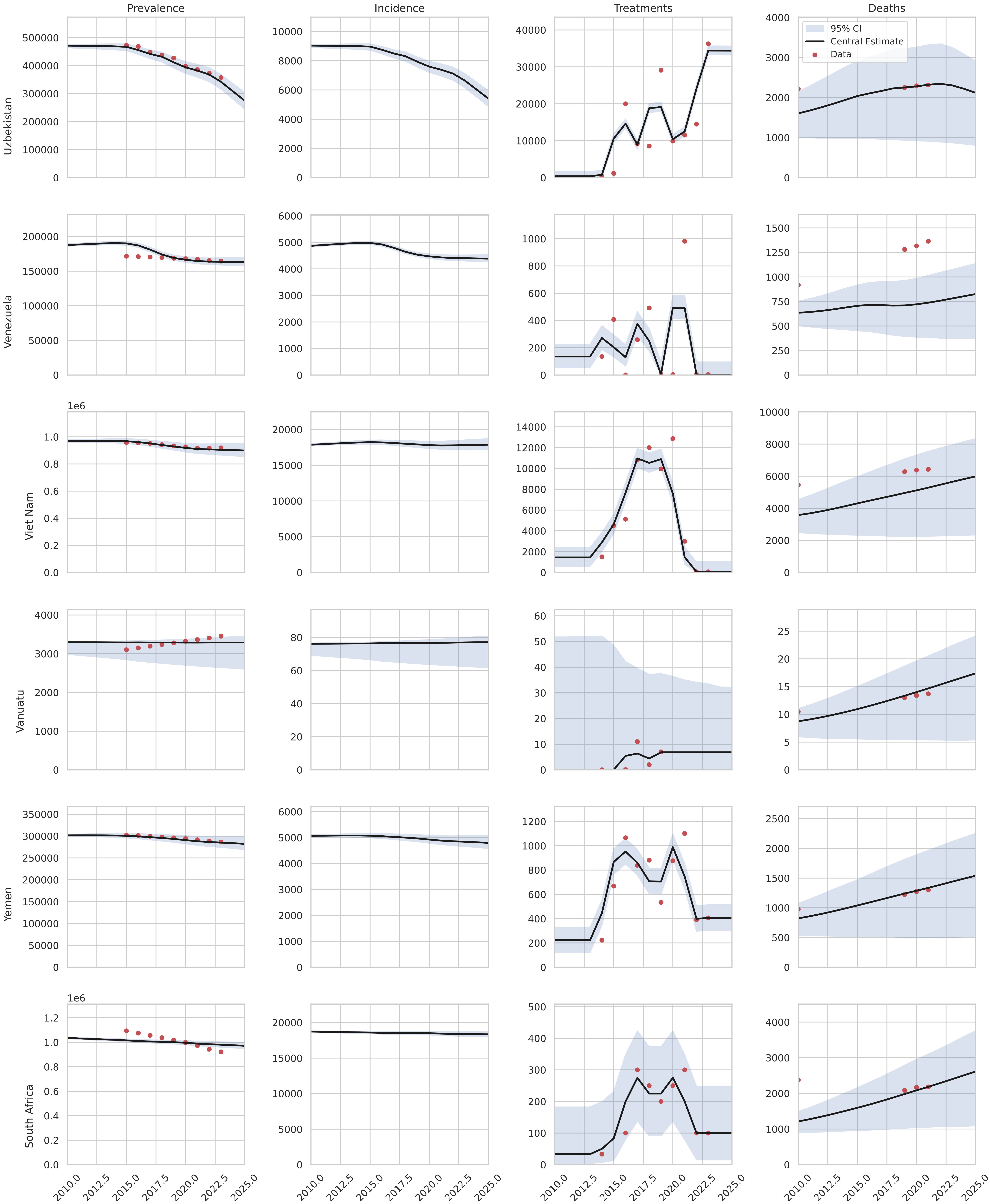

Prevalence
Incidence
Treatments
Deaths
95% CI
Central Estimate
Data
Uzbekistan
Venezuela
Viet Nam
Vanuatu
Yemen
South Africa
1e6
2010.0
2012.5
2015.0
2017.5
2020.0
2022.5
2025.0
