# Supplementary material for: Cost-benefit analysis of a potential hepatitis C vaccine: a modelling study
Source: BMC Infect Dis. 2026 Jul 2;26:1715. doi: 10.1186/s12879-026-13858-7 (PMC13602624; doi:10.1186/s12879-026-13858-7)
Supplement: Supplementary file 2 [file 12879_2026_13858_MOESM2_ESM.docx]

Supplementary appendix

# Contents

[Section A:	Model description	2](#_Toc213780787)

[Section B:	Economic analysis	12](#_Toc213780788)

[Section C:	Model calibration	15](#_Toc213780789)

[Section D:	Outcomes on a regional level	18](#_Toc213780790)

[Section E:	Country-level benefit-cost ratios	22](#_Toc213780791)

[References	29](#_Toc213780792)

# Tables

[**Table 1. Country-specific data and processing.**	4](#_Toc215494602)

[**Table 2. Global inputs.**	8](#_Toc215494603)

[**Table 3. Availability of country-specific data.**	9](#_Toc215494604)

[**Table 4. Economic model inputs.** Costs are in 2024 US$.	13](#_Toc215494605)

[**Table 5. Incidence and prevalence estimates: model outputs, World Health Organization and © CDA Foundation (2).**	15](#_Toc215494606)

[**Table 6. Vaccine-attributable outcomes by region.**	18](#_Toc215494607)

[**Table 7. Benefit-cost ratios for the five scenarios per country..**	22](#_Toc215494608)

[**Table 8. Correlation between benefit-cost ratios and predictors.**	28](#_Toc215494609)

# Section A: Model description

We developed a deterministic compartmental model of hepatitis C transmission, liver disease progression and care cascade pathway. The flow between compartments is governed by ordinary differential equations of a SIRS model (Susceptible - Infectious - Recovered - Susceptible). The model ran from 2000 to 2050 in time-steps of a quarter of a year.

Tables 1 and 2 provide a detailed explanation of the input data described in the following.

## Population groups

The following mutually exclusive groups were defined in the model:

- People who inject drugs, females
- People who inject drugs, males
- People in prison, females
- People in prison, males
- People who inject drugs in prison, females
- People who inject drugs in prison, males
- Remaining general population, females (ages 0-9, 10-17, 18-64 and 65+ years)
- Remaining general population, males (ages 0-9, 10-17, 18-64 and 65+ years)

## Disease stages

The model included compartments representing key stages of hepatitis C infection and liver disease progression, defined according to the METAVIR scoring system. Stages included acute infection; chronic infection with liver fibrosis stages F0–F4; decompensated cirrhosis (DC); hepatocellular carcinoma (HCC); and in treatment.

## Susceptible compartments

Individuals were classified as susceptible to hepatitis C infection if they had never been exposed to the virus (infection naïve) or if they had previously cleared the infection either spontaneously or following treatment.

## Hepatitis C transmission

Transmission occurs due to injecting risk behaviours amongst people who inject drugs in the community and in prison. It was built dynamically in the model based on the RNA prevalence and population size within each group. Transmission could occur between:

- People who inject drugs in prison, females ↔ People who inject drugs in prison, females
- People who inject drugs in prison, males ↔ People who inject drugs in prison, males
- People who inject drugs in prison, females ↔ People who inject drugs in prison, males
- People who inject drugs, females ↔ People who inject drugs, females
- People who inject drugs, males ↔ People who inject drugs, males
- People who inject drugs, females ↔ People who inject drugs, males

The force of infection was calibrated separately for each group to fit observed prevalence and incidence estimates.

Additionally, for settings with high prevalence in the general population (defined as an RNA prevalence greater than 2%), a dynamic force of infection was defined for the adult general population, which was calibrated to fit overall prevalence estimates.

## Acute infection

Individuals with a new hepatitis C infection remained in the acute phase for up to one year. Within this time frame, 42% of females and 20% of males were assumed to clear the virus spontaneously while the remaining developed chronic infection. For primary infections, individuals who achieved spontaneous clearance did not develop liver fibrosis. Those who were re-infected following a history of chronic infection (cured with treatment) were assumed to re-enter an acute phase at the highest fibrosis stage reached pre-treatment. Spontaneous clearance of acute infection could occur at any stage.

## Chronic infection

Chronic infection progressed sequentially from F0 to F4, F4 to DC or HCC, and from DC to HCC. Post-treatment virological relapse occurred at the DC or HCC stages. Hepatitis C-related mortality was assigned to the F4, DC and HCC, and a 50% reduction in mortality post DC or HCC cure was implemented. Mortality rates were calibrated to reproduce annual numbers of hepatitis C-related deaths.

## Care cascade

At any stage of infection, individuals could be undiagnosed, diagnosed antibody positive, diagnosed RNA positive, or in treatment.

## Antibody testing

Testing rates were obtained from the literature for key population groups and calibrated to reproduce national hepatitis C notifications trends for the general population.

## RNA testing

It was assumed that 30% of the general population and 80% of people who inject drugs diagnosed antibody positive received an RNA diagnosis. These values were varied in the calibration.

## Treatment

For interferon-based therapies, treatment duration was 46 weeks and the probability of achieving sustained virologic response (SVR) 50%. Following the introduction of direct-acting antiviral agents (DAAs) in 2016, treatment duration decreased to 12 weeks with a 95% chance of SVR. There was no limit on the number of DAA treatment courses an individual could receive. Treatment allocation in the model was both population- and disease stage-specific, based on the burden of disease and the proportion in each stage.

## Sources and sinks

Each year, individuals entered the 0–9 year old age group according to the annual number of live births by sex. Individuals exited the model according to all-cause mortality and hepatitis C-related mortality rates. Individuals could also enter or leave the model according to net overseas migration.

## Number of model runs

A convergence check was performed to determine the number of model runs required for convergence. In an illustrative setting (Vietnam), we ran n=100, 250, 500, 750 and 1000 simulations and calculated the median hepatitis C prevalence in 2026 and the width of the 95% confidence interval (CI). Convergence was defined as a <5% change in the median and CI width between two consecutive runs. While the median was converged at 100 runs, the width of the CI only stabilised at 750 runs. Convergence outcomes are shown in Figure 1. The model was consequently run 1000 times in each setting.

**Table 1. Country-specific data and processing.**

| **Model parameter** | **Data Sources** | **Data Processing** | **Dataset usage in model** |
| --- | --- | --- | --- |
| **Total population size and transfers** | | | |
| Male/female population size aged: 0-9 years, 10-17 years, 18-64 years, 65+ years | UN (1) | Subtracted population sizes of people who inject drugs and people in prison from the 18-64 year-old groups (see *Number of males/females in prison* and *Number of males/females who inject drugs*) | Direct input |
| Male births/Female births | UN (1) | Entry into the 0-9 years population groups | Direct input |
| Male/Female ageing transfers for 0-9 years to 10-17 years; 10-17 years to 18-64 years; 18-64 years to 65+ years | UN (1) | Used population size from age bracket 1 year younger than age group transferring to the year prior (*e.g.* ageing into 10-17 years in 2022 are the 9 year olds in 2021) | Direct input |
| Male/Female all-cause mortality rate for: 0-9 years, 10-17 years, 18-64 years, 65+ years | UN (1) | Same data used for general population, and for starting point to calibrate for people in prison and people who inject drugs.  Subtracted deaths due to hepatitis C from total deaths in 18-64 and 65+ age groups, apportioned according to relative sizes of 18-64 and 65+ populations.  Mortality rate = total general deaths in age bracket divided by general population size for age bracket | Direct input and calibration |
| Net migration rate | UN (1) | Migration among people in prison and people who inject drugs set to zero | Direct input |
| **Total population: epidemiological estimates** | | | |
| Total number of people with hepatitis C | © CDA Foundation (2) | Calibrated using the force of infection among people who inject drugs and, in the community, where applicable. | Calibration |
| Annual number of hepatitis C-related deaths | GBD (3) | Calibrated to by varying the hepatitis C-related mortality rate. | Calibration |
| Disease stage distribution | Assumption, to achieve correct disease stage distribution in later years when paired with disease progression rates from the literature | Initialised in the year 2000 with 94.5% in F0-F2, 3% in F3, 2% in F4, 0.45% in DC and 0.05% in HCC. | Direct input |
| **Total population: care cascade** | | | |
| % Diagnosed | © CDA Foundation (2) |  | Input (initialisation) |
| Number newly diagnosed | © CDA Foundation (2) | Calibrated by changing population-specific testing rates. | Calibration |
| Total number of treatments | © CDA Foundation (2) |  | Input |
| Treatments allocated to each population group |  | Initial estimation using distribution of hepatitis C burden among the different groups; then calibrated to fit population-specific and total number of people living with hepatitis C. | Calibration |
| **Estimates of people who inject drugs** | | | |
| Number of males/females who inject drugs | Degenhardt L et al. (4) | *Calculating total number of people who inject drugs:*  Prevalence of injecting drug use in each country from (4). Sex-disaggregated regional averages used where country-specific data unavailable.  Multiplied prevalence by total 18-64 population size (or 18-64 population size by sex if using regional averages).  Subtracted number of people who inject drugs in prison (see *Calculating % of prisoners who have a history of injecting drug use*).  Data for calculating prevalence of injecting drug use in the United Kingdom was missing number of people who inject drugs for both Wales and Northern Ireland. Data was aggregated by assuming 0.59% prevalence (same as England), and with population data for 18-64 year-olds from the Office for National Statistics (5). | Calibration |
|  | Degenhardt L et al. (4) | *Calculating number of males/females who inject drugs:*  Proportion of people who inject drugs who are female taken from (4) (uses region average where country specific values unavailable).  Proportion female multiplied by total number of people who inject drugs. |  |
| RNA prevalence of hepatitis C virus among people who inject drugs | Degenhardt L et al. (4)  Grebely J et al. (6) | Priority given to estimates from (4). Estimates from (7)  used for additional pre-DAA values (date set to 2015).  Regional values used from (4) where country-specific data unavailable. | Calibration |
| People who inject drugs tested in past 12 months | Hajarizadeh B et al. (7) | Where range of dates given for the year of estimate, used the earliest year in range.  Regional values used where country-specific data not available. | Input |
| **Prison estimates** | | | |
| Number of males/females in prison | Fair H et al. (8) | *Calculating total number of people in prison:*  Incarceration rates per 100,000 from (8) multiplied by total 18-64 population/100,000.  United Kingdom countries aggregated as direct sum, where dates matched. | Calibration |
|  | Fair H et al. (9) | *Calculating number of male/female:*  Number of females in prison from (9).  Number of males in prison = total number in prison – females in prison.  For Bosnia and Herzegovina, total males in prison was calculated from number and proportion of females in prison in Federation and Republika Srpska as given in (9), where year was the same for both datapoints. |  |
| RNA prevalence of hepatitis C virus in prison | Degenhardt L et al. (4) | *Calculating % of people in prison who have a history of injecting drug use:*  Assume number of people who inject drugs recently incarcerated is the same as the number currently incarcerated.  Used regional estimates for % incarcerated in last 12 months from (4).  Proportion of people in prison who have a history of injecting drug use = (proportion of people who inject drugs that were incarcerated in the last 12 months * total number of people who inject drugs) / total number of people in prison  Proportion of people in prison who have a history of injecting drug use capped at 0.75. | Calibration |
|  |  | *Calculating estimated RNA prevalence in prison:*  Weighted average across people in prison with a history of injecting drug use and without. |  |
| Male/Female prison admission rate (general population) | (10-17) | *Country-specific data for total number of admissions:*  Sources other than (10) prioritised.  Processing for data from (10): number of persons entering reduced according to proportion of adults held in prison (raw data includes juvenile admissions)  Male admissions = total admissions * proportion of males in prison (interpolated using step function)  Female admissions = total admissions – male total admissions  Subtracted males/females who inject drugs admissions (see *Male/Female prison admission rate (people who inject drugs)* for calculation)  Sex-disaggregated admission rate = male or female general population admissions / 18-64 general male or female population |  |
|  | UNODC (10) | *Calculating regional averages for prison entry rate (per 100,000):*  Unweighted average across adjusted admission rate data for all available years and countries in each region.  Regional averages were assumed to apply across all years from 2000 onwards  No data was available for countries in the South Asia region (Afghanistan, Bangladesh, Bhutan, India, Iran, Sri Lanka, Maldives, Nepal, Pakistan) or the Pacific Islands States and Territories region (Fiji, Kiribati, Marshall Islands, Micronesia, Nauru, Palau, Papua New Guinea, Samoa, Solomon Islands, Tonga, Tuvalu, Vanuatu), so the regional averages for both were calculated from population-weighted estimates for the following countries: Kazakhstan, Kyrgyzstan, China, China - Hong Kong Special Administrative Region, China - Macao Special Administrative Region, Japan, Mongolia, Republic of Korea, Philippines, Singapore, Thailand, Armenia, Azerbaijan, Cyprus, Georgia, Israel, Saudi Arabia, Türkiye and United Arab Emirates. |  |
|  |  | *Calculating admission rate from regional average:*  Applies only to countries without country-specific data available.  Total admissions = regional average * (total 18-64 population)/100,000  Male admissions = total admissions * proportion of males in prison (interpolated using step function)  Female admissions = total admissions – male total admissions  Subtracted male/female PWID admissions (see *Male/Female prison admission rate (people who inject drugs)* for calculation)  Sex-disaggregated admission rate = male or female general population admissions / 18-64 general male or female population |  |
| Male/Female prison admission rate (people who inject drugs) |  | PWID male admissions = total male admissions (general) * % prisoners who are PWID  (See *Male/Female prison admission rate (general population)* and *Calculating % of prisoners who are PWID)* |  |

*For the purposes of performing calculations that involved combining data from two sources, values between years with known data were interpolated using a step function.*

**Table 2. Global inputs.**

| **Metric** | **Source(s)** | **Value(s)** |
| --- | --- | --- |
| Duration of injecting career | Fazito E et al. (18) | 17 years |
| Disease progression rates | Zeremski M et al. (19) | **Estimated Transition Intensity [95% Confidence Interval (CI)]**  F0 to F1: 0.25  F1 to F2: 0.08  F2 to F3: 0.03  F3 to F4: 0.1 |
|  | Fleming KM et al. (20) | F4 to DC: 5.5% [95% CI: 4.8%-6.2%] |
|  | Bartolomeo N et al. (44) | F4 to HCC: 0.015 |
|  | Planas R et al. (45), Hep C Calculator (30) | **5-year cumulative probability converted to annual rate**  DC to HCC: 0.068 |
| Male/female spontaneous clearance rates | (21-23) | Female: 0.42 [Range: 0.35-0.48]  Male: 0.20 [Range: 0.12-0.23] |
| Average duration for spontaneous viral clearance | Aisyah DN et al. (24) | 1 year |
| Susceptible (cured) relapse rates | Gastroenterological Society of Australia (25) | To DC: 2% |
|  | Gao YX et al. (26) | **Calculated with exponential decay formula**  To HCC: 0.18 (range:0.14-0.24) |
| Reduction in mortality post cure | Assumptions^b^ | Post DC cure: 50%  Post HCC cure: 50% |
| Average treatment duration | Thompson AJ et al. (27) | Interferon-based regimens: 24 weeks  Interferon-free treatments (DAAs): 12 weeks |
| Sustained virologic response | Thompson AJ et al. (27) | Interferon-based regimens: 50%  Interferon-free treatments (DAAs): 95% |
| ^a^ Disease progression rates (F0 to F4) were sourced from Zeremski et al. (19), with point estimates set at the lower end of the reported ranges. This was informed by manual calibration and is broadly consistent with the lower rates used in the Hep C Calculator (30).  ^b^ Dang et al. (<https://doi.org/10.1002/hep.30988>) found that, for HCC, SVR was associated with a 66% lower risk of 5-year liver related mortality compared to untreated patients (hazard ratio 0.34; 95% CI, 0.13-0.88; P = 0.026). Our assumed reduction of 50% is conservative reflecting uncertainty in the generalisability of these findings across the diverse low- and middle-income settings modelled in this analysis. | | |

**Table 3. Country-specific data, including year of estimate (if applicable) and method of estimation based on data availability (country-specific estimate vs. derived from regional estimate).** Regional testing rates were halved to reflect lower observed testing rates in countries without country-specific data.

| Country | PWID population (n) | PWID female proportion | PWID HCV prevalence | PWID testing rate (12months) | % of prisoners who inject drugs | Total prison admissions (n) |
| --- | --- | --- | --- | --- | --- | --- |
| Afghanistan | 57,808 (2019) Country | 0.1% (2019) Country | 37.5% (2014) Country | 6.9% (2015) Regional | 18.5% Regional | 48,957 (2021) Regional |
| Albania | 4,102 (2019) Country | 0.0% (2019) Country | 34.0% (2011) Country | 21.5% (2015) Regional | 74.0% Regional | 4,937 (2021) Country |
| United Arab Emirates | 2,437 (2015) Regional | 1.9% (2015) Regional | 30.5% Regional | 10.3% (2015) Regional | 4.9% Regional | 110,582 (2014) Country |
| Argentina | 6,171 (2017) Country | 22.0% (2017) Regional | 54.6% (2001) Country | 16.1% (2015) Regional | 11.3% Regional | 31,071 (2020) Country |
| Armenia | 6,281 (2018) Country | 1.1% (2018) Country | 49.2% (2018) Country | 18.2% (2015) Regional | 19.5% Regional | 1,571 (2022) Country |
| Australia | 73,040 (2016) Country | 33.4% (2016) Country | 53.0% (2018) Country | 52.0% (2019) Country | 24.0% Regional | 61,618 (2021) Country |
| Austria | 8,790 (2015) Country | 28.8% (2015) Country | 61.8% (2016) Country | 43.5% (2008) Country | 74.0% Regional | 8,680 (2021) Country |
| Azerbaijan | 30,010 (2011) Country | 1.8% (2011) Country | 59.3% (2010) Country | 16.7% (2008) Country | 19.5% Regional | 7,569 (2020) Country |
| Burundi | 3,994 (2015) Regional | 12.2% (2015) Country | 5.5% (2017) Country | 2.4% (2017) Country | 32.1% Regional | 22,628 (2020) Regional |
| Belgium | 4,102 (2019) Country | 20.3% (2019) Country | 48.8% (2017) Country | 76.0% (2020) Country | 74.0% Regional | 16,911 (2020) Country |
| Benin | 4,810 (2015) Regional | 12.2% (2015) Country | 15.3% Regional | 2.7% (2015) Regional | 32.1% Regional | 7,661 (2017) Country |
| Burkina Faso | 7,646 (2015) Regional | 12.5% (2015) Regional | 15.3% Regional | 2.7% (2015) Regional | 32.1% Regional | 38,521 (2020) Regional |
| Bangladesh | 52,749 (2016) Country | 0.1% (2016) Country | 31.0% (2012) Country | 6.9% (2015) Regional | 18.5% Regional | 205,199 (2021) Regional |
| Bulgaria | 11,864 (2005) Country | 19.2% (2005) Country | 67.8% (2012) Country | 43.9% (2012) Country | 19.5% Regional | 15,970 (2020) Country |
| Bahrain | 352 (2015) Regional | 3.1% (2015) Regional | 30.5% Regional | 10.3% (2015) Regional | 4.9% Regional | 4,372 (2017) Regional |
| Brazil | 127,173 (2015) Country | 22.0% (2015) Regional | 48.6% (2016) Country | 16.1% (2015) Regional | 11.3% Regional | 383,292 (2020) Country |
| Bhutan | 371 (2015) Regional | 1.3% (2015) Regional | 34.5% Regional | 6.9% (2015) Regional | 10.0% Regional | 897 (2014) Regional |
| Central African Republic | 2,668 (2015) Regional | 11.5% (2015) Regional | 0.0% Country | 2.7% (2015) Regional | 32.1% Regional | 8,302 (2015) Regional |
| Canada | 84,014 (2016) Country | 32.2% (2016) Country | 38.6% (2017) Country | 33.1% (2017) Country | 49.5% Regional | 226,159 (2018) Country |
| Switzerland | 8,204 (2006) Country | 21.7% (2006) Country | 74.6% (2016) Country | 26.7% (2013) Country | 74.0% Regional | 34,352 (2021) Country |
| Chile | 26,830 (2006) Country | 22.0% (2006) Regional | 43.7% Regional | 16.1% (2015) Regional | 11.3% Regional | 60,403 (2021) Country |
| China | 1,399,515 (2005) Country | 17.2% (2005) Country | 49.0% (2018) Country | 6.6% (2015) Regional | 75.0% Regional | 1,604,394 (2015) Country |
| Cameroon | 874 (2018) Country | 0.0% (2018) Country | 15.3% Regional | 2.7% (2015) Regional | 32.1% Regional | 15,018 (2016) Country |
| DR Congo | 1,457 (2016) Country | 5.8% (2016) Regional | 15.3% Regional | 2.7% (2015) Regional | 32.1% Regional | 145,334 (2015) Regional |
| Colombia | 20,880 (2015) Regional | 21.1% (2015) Country | 30.5% (2014) Country | 71.9% (2019) Country | 11.3% Regional | 15,402 (2021) Country |
| Cuba | 18,736 (2015) Regional | 16.6% (2015) Regional | 0.0% Country | 16.1% (2015) Regional | 35.5% Regional | 43,078 (2020) Regional |
| Czech Republic | 28,265 (2019) Country | 25.7% (2019) Country | 17.4% (2013) Country | 18.2% (2015) Regional | 19.5% Regional | 9,533 (2021) Country |
| Germany | 75,884 (2000) Country | 24.4% (2000) Country | 62.9% (2015) Country | 51.4% (2014) Country | 74.0% Regional | 150,277 (2020) Country |
| Denmark | 9,669 (2009) Country | 21.1% (2009) Country | 65.6% (2009) Country | 21.5% (2015) Regional | 74.0% Regional | 8,909 (2021) Country |
| Dominican Republic | 12,038 (2015) Regional | 16.7% (2015) Regional | 43.6% Regional | 16.1% (2015) Regional | 35.5% Regional | 5,437 (2020) Country |
| Algeria | 9,906 (2019) Country | 0.0% (2019) Country | 30.5% Regional | 10.3% (2015) Regional | 4.9% Regional | 45,262 (2015) Country |
| Ecuador | 6,401 (2015) Regional | 20.8% (2015) Regional | 43.7% Regional | 16.1% (2015) Regional | 11.3% Regional | 75,130 (2015) Country |
| Egypt | 26,083 (2015) Regional | 5.1% (2015) Regional | 49.5% (2000) Country | 10.3% (2015) Regional | 4.9% Regional | 322,480 (2020) Regional |
| Spain | 5,274 (2019) Country | 18.4% (2019) Country | 66.1% (2018) Country | 46.7% (2019) Country | 74.0% Regional | 30,108 (2020) Country |
| Estonia | 4,885 (2015) Country | 22.1% (2015) Country | 79.2% (2016) Country | 59.0% (2019) Country | 19.5% Regional | 1,235 (2021) Country |
| Ethiopia | 81,284 (2019) Country | 6.1% (2019) Country | 3.4% (2015) Country | 8.3% (2019) Country | 32.1% Regional | 213,274 (2020) Regional |
| Finland | 9,083 (2012) Country | 21.7% (2012) Regional | 73.7% (2014) Country | 21.5% (2015) Regional | 74.0% Regional | 4,169 (2021) Country |
| Fiji | 996 (2015) Regional | 18.2% (2015) Regional | 38.8% Regional | 26.0% (2015) Regional | 35.5% Regional | 1,054 (2019) Regional |
| France | 73,540 (2019) Country | 20.5% (2019) Country | 54.8% (2013) Country | 30.0% (2015) Country | 74.0% Regional | 76,248 (2021) Country |
| Gabon | 918 (2015) Regional | 11.8% (2015) Regional | 15.3% Regional | 2.7% (2015) Regional | 32.1% Regional | 3,968 (2018) Regional |
| United Kingdom | 137,395 (2010) Country | 26.1% (2010) Regional | 52.7% (2018) Country | 43.6% (2020) Country | 74.0% Regional | 138,807 (2013) Country |
| Georgia | 75,374 (2004) Country | 2.1% (2004) Country | 61.9% (2017) Country | 44.3% (2017) Country | 19.5% Regional | 6,918 (2020) Country |
| Ghana | 11,654 (2018) Country | 9.1% (2018) Country | 2.3% (2018) Country | 0.0% (2018) Country | 32.1% Regional | 58,321 (2021) Regional |
| Gambia | 944 (2015) Regional | 12.2% (2015) Regional | 15.3% Regional | 2.7% (2015) Regional | 32.1% Regional | 4,620 (2021) Regional |
| Greece | 1,758 (2018) Country | 17.6% (2018) Country | 66.8% (2017) Country | 67.5% (2015) Country | 74.0% Regional | 5,545 (2021) Country |
| Croatia | 3,516 (2015) Country | 18.8% (2015) Country | 36.7% (2015) Country | 35.6% (2015) Country | 74.0% Regional | 8,408 (2021) Country |
| Hungary | 4,536 (2014) Country | 26.1% (2014) Country | 35.9% (2013) Country | 33.1% (2014) Country | 19.5% Regional | 16,492 (2020) Country |
| Indonesia | 111,480 (2012) Country | 5.4% (2012) Country | 89.2% (2015) Country | 2.1% (2019) Country | 75.0% Regional | 316,615 (2020) Regional |
| India | 634,438 (2018) Country | 1.9% (2018) Country | 49.5% (2018) Country | 2.4% (2019) Country | 18.5% Regional | 1,700,045 (2019) Regional |
| Ireland | 4,981 (2000) Country | 28.8% (2000) Country | 77.2% (2015) Country | 33.1% (2018) Country | 74.0% Regional | 6,133 (2021) Country |
| Iran | 127,899 (2018) Country | 3.1% (2018) Country | 39.4% (2019) Country | 5.9% (2020) Country | 18.5% Regional | 107,365 (2020) Regional |
| Iraq | 7,478 (2015) Regional | 5.5% (2015) Country | 30.5% Regional | 10.3% (2015) Regional | 4.9% Regional | 127,060 (2021) Regional |
| Iceland | 428 (2015) Regional | 21.0% (2015) Country | 63.0% (2000) Country | 21.5% (2015) Regional | 74.0% Regional | 233 (2020) Country |
| Israel | 2,303 (2015) Regional | 5.4% (2015) Regional | 34.0% Country | 10.3% (2015) Regional | 4.9% Regional | 53,494 (2014) Country |
| Italy | 187,805 (2000) Country | 18.6% (2000) Country | 53.3% (2016) Country | 21.5% (2015) Regional | 74.0% Regional | 36,554 (2021) Country |
| Jordan | 1,972 (2015) Regional | 4.6% (2015) Regional | 30.5% Regional | 10.3% (2015) Regional | 4.9% Regional | 32,052 (2020) Regional |
| Japan | 191,812 (2004) Country | 14.3% (2004) Regional | 64.8% (2000) Country | 6.6% (2015) Regional | 75.0% Regional | 31,714 (2020) Country |
| Kazakhstan | 65,843 (2006) Country | 14.9% (2006) Country | 58.8% (2005) Country | 2.9% (2015) Regional | 51.6% Regional | 19,454 (2017) Country |
| Kenya | 20,977 (2020) Country | 11.7% (2020) Country | 20.0% (2015) Country | 2.7% (2015) Regional | 32.1% Regional | 126,768 (2017) Country |
| Kyrgyzstan | 16,315 (2017) Country | 19.2% (2017) Country | 43.9% (2013) Country | 2.9% (2015) Regional | 51.6% Regional | 4,981 (2015) Country |
| Cambodia | 2,459 (2017) Country | 19.3% (2017) Country | 29.2% (2017) Country | 18.4% (2017) Country | 75.0% Regional | 19,269 (2020) Regional |
| Republic of Korea | 50,203 (2015) Regional | 14.1% (2015) Country | 50.6% (2000) Country | 6.6% (2015) Regional | 75.0% Regional | 113,742 (2017) Country |
| Lebanon | 5,535 (2015) Country | 1.8% (2015) Country | 22.0% (2015) Country | 10.3% (2015) Regional | 4.9% Regional | 16,822 (2020) Regional |
| Libya | 1,165 (2001) Country | 1.5% (2001) Regional | 86.1% (2008) Country | 10.3% (2015) Regional | 4.9% Regional | 19,879 (2017) Regional |
| Lithuania | 5,583 (2016) Country | 20.2% (2016) Country | 65.6% (2012) Country | 44.3% (2008) Country | 19.5% Regional | 5,865 (2021) Country |
| Luxembourg | 1,465 (2009) Country | 21.7% (2009) Regional | 81.3% (2005) Country | 21.5% (2015) Regional | 74.0% Regional | 751 (2020) Country |
| Latvia | 4,885 (2016) Country | 28.9% (2016) Country | 69.2% (2012) Country | 24.1% (2007) Country | 19.5% Regional | 3,156 (2021) Country |
| Morocco | 18,355 (2011) Country | 2.7% (2011) Country | 38.1% (2015) Country | 20.7% (2017) Country | 4.9% Regional | 111,123 (2019) Country |
| Moldova | 8,026 (2008) Country | 19.9% (2008) Country | 50.0% (2013) Country | 30.4% (2020) Country | 19.5% Regional | 2,527 (2020) Country |
| Madagascar | 10,780 (2014) Country | 17.5% (2014) Country | 5.6% (2012) Country | 2.7% (2015) Regional | 32.1% Regional | 14,056 (2015) Country |
| Mexico | 59,830 (2016) Country | 26.9% (2016) Country | 95.3% (2005) Country | 16.1% (2015) Regional | 11.3% Regional | 119,188 (2021) Country |
| Malta | 635 (2015) Regional | 21.2% (2015) Regional | 26.9% (2012) Country | 21.5% (2015) Regional | 74.0% Regional | 860 (2021) Country |
| Mongolia | 2,054 (2015) Regional | 15.4% (2015) Regional | 40.1% Regional | 6.6% (2015) Regional | 35.5% Regional | 7,220 (2017) Country |
| Mozambique | 19,229 (2014) Country | 5.0% (2014) Country | 43.6% (2017) Country | 2.7% (2015) Regional | 32.1% Regional | 30,520 (2020) Country |
| Malaysia | 78,692 (2017) Country | 3.0% (2017) Country | 49.5% (2019) Country | 21.6% (2016) Country | 75.0% Regional | 39,497 (2021) Regional |
| Nigeria | 103,426 (2018) Country | 11.9% (2018) Country | 5.8% (2010) Country | 2.7% (2015) Regional | 32.1% Regional | 391,923 (2021) Regional |
| Netherlands | 1,758 (2001) Country | 25.0% (2001) Country | 61.0% (2012) Country | 21.5% (2015) Regional | 74.0% Regional | 21,735 (2020) Country |
| Norway | 4,981 (2018) Country | 30.7% (2018) Country | 64.7% (2011) Country | 21.5% (2015) Regional | 74.0% Regional | 8,943 (2021) Country |
| Nepal | 27,459 (2011) Country | 5.3% (2011) Country | 21.8% (2018) Country | 46.2% (2014) Country | 18.5% Regional | 36,075 (2021) Regional |
| New Zealand | 16,684 (2006) Country | 40.9% (2006) Country | 71.0% (2016) Country | 39.7% (2018) Country | 24.0% Regional | 12,674 (2021) Country |
| Oman | 1,025 (2015) Regional | 3.1% (2015) Regional | 75.5% (2018) Country | 10.3% (2015) Regional | 4.9% Regional | 12,345 (2015) Regional |
| Pakistan | 359,129 (2011) Country | 0.5% (2011) Country | 54.5% (2015) Country | 0.9% (2014) Country | 18.5% Regional | 287,621 (2020) Regional |
| Panama | 1,616 (2015) Regional | 20.6% (2015) Regional | 43.7% Regional | 16.1% (2015) Regional | 11.3% Regional | 16,844 (2015) Country |
| Peru | 13,680 (2015) Regional | 20.8% (2015) Regional | 43.7% Regional | 16.1% (2015) Regional | 11.3% Regional | 17,000 (2021) Country |
| Philippines | 13,115 (2015) Country | 3.9% (2015) Country | 36.0% (2010) Country | 6.6% (2015) Regional | 75.0% Regional | 140,827 (2019) Country |
| Papua New Guinea | 6,130 (2015) Regional | 17.4% (2015) Regional | 38.8% Regional | 26.0% (2015) Regional | 35.5% Regional | 11,071 (2019) Regional |
| Poland | 221,507 (2015) Regional | 25.2% (2015) Country | 58.7% (2005) Country | 35.8% (2012) Country | 19.5% Regional | 73,503 (2021) Country |
| Puerto Rico | 12,496 (2002) Country | 14.9% (2002) Country | 78.4% (2015) Country | 32.2% (2010) Country | 35.5% Regional | 5,933 (2021) Country |
| Portugal | 7,325 (2015) Country | 24.0% (2015) Country | 86.1% (2014) Country | 21.5% (2015) Regional | 74.0% Regional | 4,448 (2021) Country |
| Qatar | 442 (2015) Regional | 1.9% (2015) Regional | 50.6% (2008) Country | 10.3% (2015) Regional | 4.9% Regional | 5,996 (2013) Regional |
| Romania | 54,437 (2014) Country | 18.8% (2014) Country | 62.9% Country | 42.1% (2009) Country | 19.5% Regional | 11,114 (2023) Country |
| Russian Federation | 1,025,655 (2016) Country | 28.3% (2016) Country | 72.5% (2010) Country | 37.1% (2003) Country | 19.5% Regional | 197,973 (2020) Country |
| Rwanda | 1,165 (2011) Country | 18.2% (2011) Country | 15.3% Regional | 2.7% (2015) Regional | 32.1% Regional | 23,952 (2021) Regional |
| Saudi Arabia | 6,530 (2015) Regional | 4.0% (2015) Regional | 54.5% (2018) Country | 10.3% (2015) Regional | 4.9% Regional | 848 (2015) Country |
| Sudan | 8,240 (2015) Regional | 5.4% (2015) Regional | 30.5% Regional | 10.3% (2015) Regional | 4.9% Regional | 126,007 (2017) Regional |
| El Salvador | 4,024 (2014) Country | 22.0% (2014) Regional | 53.1% (2018) Country | 16.1% (2015) Regional | 11.3% Regional | 196 (2021) Country |
| Slovakia | 12,562 (2006) Country | 30.2% (2006) Country | 38.5% (2012) Country | 18.2% (2015) Regional | 19.5% Regional | 7,691 (2021) Country |
| Slovenia | 3,223 (2012) Country | 24.0% (2012) Country | 28.6% (2012) Country | 21.5% (2015) Regional | 74.0% Regional | 2,099 (2021) Country |
| Sweden | 4,688 (2011) Country | 26.4% (2011) Country | 65.2% (2017) Country | 21.5% (2015) Regional | 74.0% Regional | 19,725 (2020) Country |
| Syria | 5,343 (2015) Regional | 5.3% (2015) Regional | 3.3% (2014) Country | 10.3% (2015) Regional | 4.9% Regional | 54,094 (2004) Regional |
| Chad | 5,371 (2015) Regional | 12.1% (2015) Regional | 15.3% Regional | 2.7% (2015) Regional | 32.1% Regional | 28,976 (2018) Regional |
| Thailand | 29,509 (2013) Country | 14.2% (2013) Country | 53.6% (2008) Country | 3.0% (2017) Country | 75.0% Regional | 163,004 (2021) Country |
| Tajikistan | 15,150 (2014) Country | 13.8% (2014) Country | 61.3% (2004) Country | 2.0% (2004) Country | 51.6% Regional | 9,116 (2020) Regional |
| Tunisia | 3,794 (2015) Regional | 5.2% (2015) Country | 17.2% (2009) Country | 10.3% (2015) Regional | 4.9% Regional | 35,544 (2021) Regional |
| Turkey | 25,698 (2015) Regional | 5.2% (2015) Country | 44.5% (2016) Country | 10.3% (2015) Regional | 4.9% Regional | 279,231 (2020) Country |
| Taiwan | 23,285 (2015) Regional | 14.3% (2015) Country | 91.9% (2013) Country | 6.6% (2015) Regional | 35.5% Regional | 27,333 (2000) Regional |
| Tanzania | 232,200 (2012) Country | 10.8% (2012) Regional | 23.1% (2017) Country | 0.9% (2011) Country | 32.1% Regional | 116,058 (2022) Regional |
| Uganda | 5,535 (2019) Country | 18.4% (2019) Country | 15.3% Regional | 3.6% (2017) Country | 32.1% Regional | 110,517 (2014) Country |
| Ukraine | 206,580 (2020) Country | 23.1% (2020) Country | 60.6% (2018) Country | 32.1% (2015) Country | 19.5% Regional | 20,591 (2020) Country |
| United States of America | 1,512,250 (2018) Country | 30.3% (2018) Country | 53.5% (2018) Country | 54.8% (2019) Country | 49.5% Regional | 421,006 (2021) Country |
| Uzbekistan | 31,756 (2011) Country | 15.4% (2011) Regional | 51.7% (2001) Country | 2.9% (2015) Regional | 51.6% Regional | 32,671 (2022) Regional |
| Venezuela | 12,736 (2015) Regional | 20.9% (2015) Regional | 43.7% Regional | 16.1% (2015) Regional | 11.3% Regional | 69,033 (2020) Regional |
| Viet Nam | 116,945 (2018) Country | 4.8% (2018) Country | 69.2% (2018) Country | 13.0% (2017) Country | 75.0% Regional | 111,954 (2019) Regional |
| Vanuatu | 193 (2015) Regional | 17.0% (2015) Regional | 38.8% Regional | 26.0% (2015) Regional | 35.5% Regional | 344 (2020) Regional |
| Yemen | 5,392 (2015) Regional | 5.3% (2015) Regional | 30.5% Regional | 10.3% (2015) Regional | 4.9% Regional | 97,618 (2017) Regional |
| South Africa | 47,780 (2014) Country | 15.6% (2014) Country | 54.7% (2017) Country | 2.7% (2015) Regional | 32.1% Regional | 108,616 (2020) Regional |

Prevalence refers to hepatitis C virus RNA prevalence.

Countries are marked with a tick where country-specific data was available, and a cross where regional estimates were used.

**Figure 1. Convergence of hepatitis C prevalence estimates by number of model runs, Vietnam.** Median hepatitis C prevalence in 2026 and 95% Confidence Interval (CI) estimated from 100–1000 runs. Left panel: median point estimate with 95% CI error bars by sample size. Right panel: 95% CI width (upper bound minus lower bound) by sample size. Convergence was defined as a <5% change in median and CI width between two consecutive sample sizes.

# Section B: Economic analysis

Economic analyses adopted a series of generalised approaches to estimate all modelled hepatitis C associated costs (28-30). All presented values represent the economic cost of commodities and services, valued in 2024 consumer price index (CPI) adjusted United States Dollars (US$). CPI adjustment was made using annual inflators from the United States Bureau of Labor Statistics *(31)*.

## Intervention Costs

Unit costs for currently available hepatitis C interventions (diagnostic tests and treatment) were estimated using an ingredients-based approach. Each intervention included a commodity and service delivery (human resource) cost component.

For all modelled countries, commodity costs of diagnostic tests (HCV antibody and HCV RNA viral load quantification) and treatments (direct acting antivirals; DAAs) were assumed equal. Unit prices were sourced from the Clinton Health Access Initiative (CHAI) 2023 Hepatitis C Market Intelligence Report (32). We assumed hepatitis C antibody testing to primarily (90%) use rapid diagnostic tests over laboratory-based assays and conservatively assumed negotiated ceiling price for DAAs (US$60). In line with programmatic costing methods used by the Lives Saved Tool (LiST) (33), we added an additional 21% to all commodity costs (Table 4), reflecting wastage (5%) and associated logistics (e.g., storage, disposal; 16%).

Given hepatitis C vaccines are a theoretical intervention, commodity costs were unknown for this analysis. Through consultation, a purchase price of US$5 per dose in a low-and middle-income country (LMIC; via World Bank designation for 2024 (34)) was indicated as a realistic indicative price were a product to come to market. Informed by WHO Market Information for Access for to Vaccines (MI4A) historic cost data for vaccines targeted at comparable age-demographics (HPV and adult formulation hepatitis B (HBV); Table 4), per dose vaccine costs were doubled in upper middle-income countries and increased 15-fold in high-income countries (35). Vaccine commodity costs were inflated following the same LiST approach as described for diagnostic testing and hepatitis C treatment commodities.

Service delivery (human resource) costs were estimated to be proportional to per capita Gross Domestic Product (GDP) in each modelled country (36). For each country, per capita GDP was inflated to reflect per worker GDP (37) (productivity constrained to those in employment; human capital approach), and pro-rated to reflect health worker time. We assumed a full-time employee to work 45 weeks a year and 35 hours per week. Costing allowed for two hours worker time per diagnostic test or treatment initiation (38), and five minutes per administered vaccine. Following LiST methodology, an additional 26% was added to service delivery costs to account for infrastructure and investment associated with employee payments.

The total number of diagnostic tests completed in a given year was estimated as the sum of positive tests (both antibody and RNA) plus a country specific negative test allowance (34). Negative antibody tests occurred at a rate proportional to hepatitis C prevalence within a population, while negative RNA tests occurred at a rate proportional to the spontaneous clearance rate.

The sum of each commodity and service delivery cost (intervention cost) was inflated by 15% to account for the costs of program delivery (e.g., training, administration, awareness campaigns) following LiST methodology.

## Disease Management Costs

Disease management costs associated with each health state, estimating the aggregate costs of disease monitoring and management of hepatitis C associated sequalae (e.g., cirrhosis, liver cancer), were estimated in each modelled country following methods described by Chhatwal and colleagues (30).

We assumed during asymptomatic stages of disease (F0–F4) only those who were diagnosed would incur costs, while symptomatic illness (decompensated cirrhosis and HCC) would present symptoms which require care irrespective of diagnosis. To approximate variable access to, and accessibility of, care in each modelled country; we assumed only 25% of people who require care each year receive it in low and lower-middle income countries (38). In upper-middle and high-income countries, where access to care may be improved, we increased this to 75% of people each year.

For countries with missing economic data, values were imputed using a population-weighted WHO region and 2024/25 financial year World Bank income classification average.

## Productivity Costs

The net cost of lost productivity attributable to hepatitis C were estimated as the sum of presenteeism (reduced productivity while engaged in employment), absenteeism (days of missed employment due to illness), and productive years lost through mortality (Table 4). This approach followed a method previously described by Scott and colleagues (28).

Estimation of productivity losses took a human capital approach in each country, with salaries estimated at GDP per worker. Productivity increases following clearance of hepatitis C virus, irrespective of underlying mechanism (DAA/SVR or spontaneous clearance), were assumed equal. Employment was assumed equal among the general (18-65-year-old) population and people who inject drugs, zero in prison populations, and a forced retirement age of 65-years was used.

## Years Lost Life and Disability Adjusted Life Years (DALYs)

DALYs were estimated as the sum of Years Lost Life (YLL) and Years Lost to Disability (YLD). YLL to hepatitis C mortality were estimated against a counterfactual (hepatitis C free) projection in each country (28), while YLD were only accrued in F4, decompensated cirrhosis and HCC health states (Table 4). We assumed YLD were accrued equally irrespective hepatitis C treatment status (i.e., equal disability weights applied).

**Table 4. Economic model inputs.** Costs are in 2024 US$.

| **Parameter** | **Value** | **Source / notes** |
| --- | --- | --- |
| **Intervention costs** | | |
| Hepatitis C antibody test | $1.18 (range: $0.98 to $3.00) | CHAI (32) |
| Hepatitis C RNA test | $13.89 (range: $9.88 to $16.98) |  |
| DAA treatment | $60 (range: $40 to $120) | *Assumption* |
| Inflation due to wastage (commodity costs) | 5% | LiST (33) |
| Inflation due to logistics (commodity costs) | 16% |  |
| Inflation due to overheads (staff wages) | 26% |  |
| Inflation due to program delivery (total costs) | 15% |  |
| **Vaccine per-dose cost** | | |
| HPV, Low Income | $4.50 | MI4A (35), **used as a reference for HCV vaccine cost.**  Analysis was limited to deidentified countries with complete procurement (doses purchased) and price per dose data. |
| HPV, Lower-Middle Income | $5.66 |  |
| HPV, Upper-Middle Income | $10.07 |  |
| HPV, High Income | $17.06 |  |
| Adult HBV, Low Income | $0.30 |  |
| Adult HBV, Lower-Middle Income | $0.35 |  |
| Adult HBV, Upper-Middle Income | $0.62 |  |
| Adult HBV, High Income | $4.54 |  |
| HCV, Low and Lower-Middle Income | $5.00 | *Assumptions* |
| HCV, Upper-Middle Income | $10.00 |  |
| HCV, High Income | $75.00 |  |
| **Access to care / disease management** | | |
| F0–F3 diagnosed, Low Income | 25% | *Assumptions* |
| F0–F3 diagnosed, Lower-Middle Income | 25% |  |
| F0–F3 diagnosed, Upper-Middle Income | 25% |  |
| F0–F3 diagnosed, High Income | 25% |  |
| F4 diagnosed, Low Income | 25% |  |
| F4 diagnosed, Lower-Middle Income | 25% |  |
| F4 diagnosed, Upper-Middle Income | 75% |  |
| F4 diagnosed, High Income | 75% |  |
| DC and HCC, Low Income | 25% |  |
| DC and HCC, Lower-Middle Income | 25% |  |
| DC and HCC, Upper-Middle Income | 75% |  |
| DC and HCC, High Income | 75% |  |
| **Productivity losses** | | |
| Absenteeism, no cirrhosis | 1.03% | DiBonaventura MD et al. (39) Younossi Z et al. (40)  Scott N et al. (28) |
| Absenteeism, no cirrhosis, cured | 1.03% |  |
| Presenteeism, no cirrhosis | 3.19% |  |
| Presenteeism, no cirrhosis, cured | 2.55% |  |
| Absenteeism, cirrhosis | 2.87% |  |
| Absenteeism, cirrhosis, cured | 1.61% |  |
| Presenteeism, cirrhosis | 4.91% |  |
| Presenteeism, cirrhosis, cured | 4.37% |  |
| **Disability weights** | | |
| Compensated cirrhosis (F4) | 0.123 (range: 0.07 to 0.176) | Martin NK et al. (41) |
| Decompensated cirrhosis (DC) | 0.220 (range: 0.156 to 0.298) | GBD 2021; Cirrhosis and other liver diseases due to hepatitis C, decompensated, with moderate anaemia (42) |
| Hepatocellular Carcinoma (HCC) | 0.540 (range: 0.377 to 0.687) | GBD 2021; Terminal phase of liver cancer due to hepatitis C (42) |
| Abbreviations: RNA: ribonucleic acid; DAA: direct-acting antiviral; HPV: human papillomavirus; HBV: hepatitis B virus; F0–F4: METAVIR scores for liver disease; DC: decompensated cirrhosis; HCC: hepatocellular carcinoma. | | |

# Section C: Model calibration

Modelled incidence and prevalence were compared against estimates by the World Health Organization’s Global Hepatitis Report 2024 (43) and the © CDA Foundation (2). All countries with available data in the Global Hepatitis Report 2024 (43) were included. The Polaris Observatory provides the incidence of paediatric and adult chronic infections; for comparability, only adult incidence was factored in, and country-specific average spontaneous clearance rates were used to calculate equivalent incidence of acute infections.

**Table 5. Incidence and prevalence estimates: model outputs, World Health Organization and © CDA Foundation (2).**

|  | **Incidence (year 2022)** | | | **Prevalent cases (year 2022)** | | |
| --- | --- | --- | --- | --- | --- | --- |
| **Country** | **Model**  **output** | **World Health Organization** | **© CDA Foundation** | **Model**  **output** | **World Health Organization** | **© CDA Foundation** |
| ***Egypt*** | 32,567 (8,491 to 64,706) | 9,708 | 6,348 | 561,366 (137,982 to 1,134,369) | 484,523 | 497,046 |
| ***Morocco*** | 2,095 (2,029 to 2,176) | 3,416 | 2,711 | 123,460 (119,122 to 128,759) | 125,566 | 123,330 |
| ***Pakistan*** | 255,488 (247,425 to 263,753) | 110,000 | 91,160 | 9,593,233 (9,264,249 to 9,930,702) | 8,790,812 | 9,548,390 |
| ***Sudan*** | 17,217 (16,688 to 17,796) | 11,876 | 3,872 | 533,984 (517,020 to 552,616) | 511,036 | 548,666 |
| ***Yemen*** | 4,860 (4,690 to 5,067) | No estimate | 3,499 | 286,414 (276,138 to 299,041) | 257,189 | 288,536 |
| ***Cameroon*** | 3,256 (3,122 to 3,412) | 3,725 | 3,253 | 155,522 (149,033 to 163,119) | 157,542 | 153,157 |
| ***DR Congo*** | 11,061 (10,689 to 11,501) | 13,104 | 8,857 | 423,912 (406,578 to 444,425) | 432,499 | 423,683 |
| ***Ethiopia*** | 15,231 (14,720 to 15,837) | 13,490 | 13,682 | 695,434 (672,352 to 723,257) | 691,928 | 697,035 |
| ***Ghana*** | 9,930 (9,637 to 10,266) | 10,714 | 9,145 | 443,144 (429,019 to 459,663) | 442,797 | 455,797 |
| ***Nigeria*** | 37,048 (35,843 to 38,446) | 27,596 | 25,652 | 1,437,890 (1,390,374 to 1,493,065) | 1,349,056 | 1,430,475 |
| ***Rwanda*** | 3,518 (3,326 to 3,684) | 3,352 | 3,696 | 127,467 (120,403 to 133,578) | 62,170 | 124,146 |
| ***United Republic of Tanzania*** | 1,907 (1,847 to 1,969) | 1,312 | 784 | 101,312 (98,187 to 104,888) | 99,576 | 100,030 |
| ***Uganda*** | 11,265 (10,793 to 11,776) | No estimate | 2,879 | 347,748 (332,933 to 363,802) | 356,043 | 345,239 |
| ***South Africa*** | 18,403 (18,100 to 18,741) | 3,159 | 13,876 | 984,333 (965,471 to 1,005,616) | 243,978 | 943,162 |
| ***Georgia*** | 3,717 (3,440 to 4,056) | 57 | 3,157 | 85,821 (79,717 to 93,028) | 48,600 | 87,554 |
| ***Kyrgyzstan*** | 5,213 (5,041 to 5,417) | 4,103 | 3,053 | 163,747 (158,109 to 170,444) | 170,824 | 165,921 |
| ***Moldova*** | 3,079 (2,872 to 3,275) | 1,626 | 2,618 | 59,183 (55,034 to 63,177) | 73,675 | 58,915 |
| ***Russian Federation*** | 72,499 (70,619 to 74,387) | 33,877 | 79,840 | 2,715,917 (2,671,367 to 2,775,476) | 2,663,597 | 2,731,435 |
| ***Ukraine*** | 33,559 (32,864 to 34,391) | 18,031 | 17,359 | 1,252,257 (1,223,506 to 1,287,167) | 1,250,706 | 1,334,369 |
| ***Uzbekistan*** | 7,126 (6,667 to 7,571) | 35,598 | 2,310 | 368,242 (342,945 to 392,659) | 1,065,843 | 372,759 |
| ***Brazil*** | 11,735 (11,306 to 12,225) | 4,028 | 3,335 | 600,890 (578,299 to 627,275) | 535,868 | 604,522 |
| ***Colombia*** | 3,946 (3,883 to 4,022) | 3,646 | 3,410 | 313,473 (308,022 to 319,868) | 309,159 | 309,515 |
| ***Mexico*** | 13,173 (12,905 to 13,505) | 5,736 | 4,960 | 689,401 (665,060 to 718,709) | 678,258 | 688,835 |
| ***Peru*** | 1,393 (1,357 to 1,436) | 814 | 789 | 56,531 (55,090 to 58,311) | 57,756 | 55,881 |
| ***China*** | 76,724 (75,190 to 78,276) | 53,383 | 52,206 | 4,184,190 (4,033,606 to 4,350,905) | 4,012,657 | 4,234,854 |
| ***Cambodia*** | 4,100 (3,893 to 4,331) | 4,625 | 4,713 | 183,674 (174,142 to 194,322) | 183,160 | 184,346 |
| ***Mongolia*** | 2,620 (2,454 to 2,741) | 1,939 | 1,731 | 131,954 (122,970 to 138,570) | 128,501 | 130,545 |
| ***Philippines*** | 8,595 (8,300 to 8,939) | 8,939 | 10,103 | 442,191 (426,588 to 460,953) | 427,470 | 438,389 |
| ***Viet Nam*** | 17,783 (17,205 to 18,442) | 17,944 | 14,864 | 907,043 (870,779 to 948,270) | 890,997 | 916,386 |
| ***Vanuatu*** | 77 (63 to 79) | 85 | 84 | 3,289 (2,661 to 3,404) | 3,048 | 3,406 |
| ***Bangladesh*** | 35,679 (34,448 to 37,146) | 17,008 | 19,639 | 1,049,779 (1,012,217 to 1,094,416) | 1,020,178 | 1,060,441 |
| ***Indonesia*** | 26,638 (25,529 to 27,952) | 39,134 | 29,860 | 1,155,818 (1,105,479 to 1,216,894) | 1,392,645 | 1,191,959 |
| ***India*** | 129,506 (124,671 to 135,296) | 144,187 | 166,181 | 5,977,799 (5,735,829 to 6,261,176) | 5,507,793 | 6,100,120 |
| ***Thailand*** | 7,639 (7,434 to 7,884) | 6,630 | 7,438 | 406,493 (393,702 to 421,770) | 374,514 | 413,804 |

# Section D: Outcomes on a regional level

**Table 6. Vaccine-attributable outcomes by region.**

| **Outcomes 2026–2050** | **Scenario 1** | **Scenario 2** | **Scenario 3** | **Scenario 4** | **Scenario 5** |
| --- | --- | --- | --- | --- | --- |
| ***High Burden Countries (n=10 countries)*** | | | | | |
| ***Vaccines administered*** | 12.8M   (9.81M to 15.2M) | 15.6M   (12.8M to 17.8M) | 30.9M   (29.4M to 32.3M) | 187M   (185M to 188M) | 4.8B   (4.79B to 4.8B) |
| ***Vaccine-averted HCV infections*** | 610k   (405k to 835k) | 886k   (750k to 1.05M) | 1.2M   (1.14M to 1.28M) | 1.42M   (1.36M to 1.51M) | 4.8M   (4.54M to 5.18M) |
| ***Vaccine-averted HCC cases*** | 810   (325 to 1.23k) | 2.23k   (923 to 3.07k) | 2.56k   (1.07k to 3.46k) | 2.73k   (1.16k to 3.67k) | 10.3k   (3.25k to 15.2k) |
| ***Vaccine-averted HCV deaths*** | 1.23k   (506 to 1.86k) | 2.65k   (1.12k to 3.75k) | 3.15k   (1.3k to 4.33k) | 3.34k   (1.42k to 4.55k) | 11.4k   (3.8k to 17.7k) |
| ***Vaccine-averted DALYs*** | 7.56k   (3.07k to 11.8k) | 19.1k   (7.97k to 27.6k) | 23.2k   (9.44k to 33.3k) | 24.2k   (10.1k to 34.4k) | 71.5k   (24.7k to 109k) |
| ***Vaccine-averted direct costs ($)*** | 170M   (104M to 350M) | 260M   (181M to 472M) | 311M   (223M to 542M) | 312M   (224M to 544M) | 358M   (252M to 631M) |
| ***Vaccine-attributable productivity gains ($)*** | 1.65B   (1.14B to 2.22B) | 1.93B   (1.51B to 2.4B) | 1.95B   (1.62B to 2.26B) | 2B   (1.67B to 2.31B) | 3.32B   (2.94B to 3.72B) |
| ***Vaccine costs ($)*** | 781M   (677M to 854M) | 878M   (790M to 941M) | 1.42B   (1.37B to 1.47B) | 4.34B   (4.28B to 4.38B) | 90.9B   (90.8B to 90.9B) |
| ***Cost per DALY averted ($)*** | 80.8k   (43.3k to 190k) | 32.3k   (16.1k to 80.1k) | 47.9k   (28.2k to 123k) | 166k   (113k to 406k) | 1.27M   (833k to 3.66M) |
| ***Benefit-cost ratio*** | 2.33   (1.69 to 3.26) | 2.5   (1.99 to 3.29) | 1.59   (1.33 to 1.94) | 0.53   (0.45 to 0.64) | 0.04   (0.04 to 0.05) |
| ***African Region (n=20 countries)*** | | | | | |
| ***Vaccines administered*** | 304k   (111k to 579k) | 704k   (525k to 954k) | 2.64M   (2.53M to 2.78M) | 119M   (119M to 119M) | 1.04B   (1.04B to 1.04B) |
| ***Vaccine-averted HCV infections*** | 18k   (4.92k to 29.6k) | 47.9k   (37.7k to 56.5k) | 81.9k   (74.6k to 88.8k) | 299k   (276k to 333k) | 1.43M   (1.33M to 1.57M) |
| ***Vaccine-averted HCC cases*** | 11   (2 to 23) | 91   (27 to 150) | 99   (30 to 160) | 195   (62 to 325) | 1.87k   (376 to 3.45k) |
| ***Vaccine-averted HCV deaths*** | 13   (3 to 28) | 172   (46 to 298) | 187   (54 to 312) | 384   (112 to 648) | 3.67k   (722 to 6.77k) |
| ***Vaccine-averted DALYs*** | 66   (12 to 155) | 790   (220 to 1.39k) | 909   (283 to 1.55k) | 1.63k   (525 to 2.75k) | 14.7k   (2.92k to 27.5k) |
| ***Vaccine-averted direct costs ($)*** | 429k   (90.5k to 1.18M) | 1.7M   (979k to 3.54M) | 2.96M   (2.06M to 5.7M) | 2.97M   (1.85M to 6.26M) | 8.18M   (4.2M to 19.2M) |
| ***Vaccine-attributable productivity gains ($)*** | 8.15M   (1.77M to 13.4M) | 21.8M   (18M to 25.6M) | 26.4M   (22.9M to 30.2M) | 56.8M   (51.7M to 63.3M) | 379M   (339M to 426M) |
| ***Vaccine costs ($)*** | 1.91M   (700k to 3.42M) | 4.96M   (3.86M to 6.29M) | 14.3M   (13.5M to 15.1M) | 615M   (614M to 616M) | 7.16B   (7.15B to 7.16B) |
| ***Cost per DALY averted ($)*** | 22.3k   (10.3k to 90.7k) | 4.12k   (1.47k to 16.4k) | 12.5k   (6.43k to 39.2k) | 376k   (222k to 1.17M) | 485k   (259k to 2.45M) |
| ***Benefit-cost ratio*** | 4.49   (2.84 to 5.18) | 4.75   (4.06 to 5.59) | 2.06   (1.83 to 2.41) | 0.1   (0.09 to 0.11) | 0.05   (0.05 to 0.06) |
| ***Region of the Americas (n=15 countries)*** | | | | | |
| ***Vaccines administered*** | 10.4M   (9.16M to 11.3M) | 11.1M   (9.95M to 11.8M) | 16.9M   (16.2M to 17.5M) | 64.6M   (63.9M to 65.2M) | 1.06B   (1.06B to 1.06B) |
| ***Vaccine-averted HCV infections*** | 404k   (310k to 516k) | 408k   (333k to 498k) | 418k   (378k to 461k) | 434k   (394k to 476k) | 634k   (593k to 685k) |
| ***Vaccine-averted HCC cases*** | 476   (200 to 703) | 639   (270 to 913) | 611   (254 to 845) | 619   (258 to 857) | 911   (375 to 1.32k) |
| ***Vaccine-averted HCV deaths*** | 1.11k   (464 to 1.66k) | 1.54k   (657 to 2.2k) | 1.55k   (649 to 2.16k) | 1.57k   (659 to 2.17k) | 2.15k   (930 to 3.03k) |
| ***Vaccine-averted DALYs*** | 5.76k   (2.45k to 8.66k) | 8.14k   (3.49k to 11.7k) | 8.43k   (3.5k to 12k) | 8.49k   (3.54k to 12.1k) | 10.6k   (4.59k to 15.1k) |
| ***Vaccine-averted direct costs ($)*** | 171M   (107M to 355M) | 174M   (116M to 346M) | 152M   (104M to 286M) | 152M   (104M to 287M) | 155M   (103M to 300M) |
| ***Vaccine-attributable productivity gains ($)*** | 1.59B   (1.11B to 2.11B) | 1.49B   (1.11B to 1.93B) | 1.38B   (1.1B to 1.66B) | 1.4B   (1.12B to 1.68B) | 1.79B   (1.5B to 2.09B) |
| ***Vaccine costs ($)*** | 752M   (664M to 812M) | 798M   (724M to 848M) | 1.22B   (1.17B to 1.25B) | 3.56B   (3.51B to 3.59B) | 52.3B   (52.2B to 52.3B) |
| ***Cost per DALY averted ($)*** | 101k   (53k to 230k) | 76.6k   (41.9k to 175k) | 126k   (81.1k to 298k) | 401k   (273k to 955k) | 4.9M   (3.44M to 11.3M) |
| ***Benefit-cost ratio*** | 2.34   (1.68 to 3.3) | 2.09   (1.59 to 2.86) | 1.26   (1.02 to 1.59) | 0.44   (0.35 to 0.55) | 0.04   (0.03 to 0.04) |
| ***Eastern Mediterranean Region (n=18 countries)*** | | | | | |
| ***Vaccines administered*** | 599k   (299k to 822k) | 1.01M   (742k to 1.21M) | 2.33M   (2.11M to 2.5M) | 40.3M   (40.1M to 40.5M) | 854M   (852M to 855M) |
| ***Vaccine-averted HCV infections*** | 61.3k   (46k to 72.8k) | 75.5k   (64.6k to 85.1k) | 94k   (83.7k to 103k) | 269k   (225k to 322k) | 2.67M   (2.15M to 3.26M) |
| ***Vaccine-averted HCC cases*** | 39   (9 to 73) | 120   (34 to 195) | 118   (32 to 188) | 256   (77 to 388) | 6.1k   (1.22k to 9.53k) |
| ***Vaccine-averted HCV deaths*** | 179   (41 to 316) | 244   (65 to 411) | 238   (67 to 390) | 362   (112 to 593) | 6.39k   (1.32k to 12k) |
| ***Vaccine-averted DALYs*** | 583   (142 to 1.07k) | 1.15k   (323 to 1.97k) | 1.21k   (349 to 1.99k) | 1.98k   (610 to 3.01k) | 39.4k   (7.75k to 66.3k) |
| ***Vaccine-averted direct costs ($)*** | 4.7M   (2.53M to 10.1M) | 8.06M   (5.16M to 15.4M) | 10.4M   (6.79M to 19.6M) | 12.2M   (7.66M to 23.9M) | 73.8M   (38.3M to 180M) |
| ***Vaccine-attributable productivity gains ($)*** | 38.4M   (23.4M to 49.7M) | 58.7M   (47M to 67.9M) | 68.3M   (57.1M to 77.3M) | 95.8M   (80.3M to 110M) | 746M   (535M to 996M) |
| ***Vaccine costs ($)*** | 5.83M   (2.46M to 8.28M) | 10.1M   (7.15M to 12.3M) | 17.8M   (15.2M to 19.8M) | 542M   (539M to 544M) | 13.1B   (13B to 13.1B) |
| ***Cost per DALY averted ($)*** | 1.93k   (-11.5k to 11.2k) | 1.81k   (-5.55k to 10.4k) | 6.19k   (-1.27k to 24.6k) | 268k   (174k to 871k) | 330k   (195k to 1.68M) |
| ***Benefit-cost ratio*** | 7.4   (6.21 to 11.4) | 6.59   (5.56 to 8.24) | 4.41   (3.82 to 5.2) | 0.2   (0.17 to 0.24) | 0.06   (0.04 to 0.09) |
| ***European Region (n=43 countries)*** | | | | | |
| ***Vaccines administered*** | 3.02M   (2.73M to 3.24M) | 4.03M   (3.77M to 4.26M) | 7.7M   (7.5M to 7.88M) | 37.5M   (37.3M to 37.7M) | 928M   (928M to 928M) |
| ***Vaccine-averted HCV infections*** | 341k   (300k to 377k) | 582k   (545k to 614k) | 1.14M   (1.11M to 1.17M) | 1.18M   (1.15M to 1.21M) | 1.77M   (1.73M to 1.82M) |
| ***Vaccine-averted HCC cases*** | 562   (226 to 788) | 2k   (803 to 2.75k) | 2.79k   (1.11k to 3.81k) | 2.82k   (1.12k to 3.85k) | 3.79k   (1.62k to 5.12k) |
| ***Vaccine-averted HCV deaths*** | 884   (352 to 1.24k) | 2.87k   (1.15k to 3.92k) | 4.04k   (1.58k to 5.49k) | 4.08k   (1.61k to 5.54k) | 5.41k   (2.32k to 7.25k) |
| ***Vaccine-averted DALYs*** | 5k   (1.91k to 7.32k) | 18.9k   (7.34k to 27k) | 26.5k   (9.98k to 37.9k) | 26.7k   (10.1k to 38.1k) | 33.4k   (13.5k to 46.6k) |
| ***Vaccine-averted direct costs ($)*** | 71M   (48.5M to 127M) | 234M   (160M to 405M) | 376M   (268M to 643M) | 377M   (269M to 645M) | 421M   (300M to 722M) |
| ***Vaccine-attributable productivity gains ($)*** | 491M   (408M to 549M) | 1.04B   (930M to 1.12B) | 1.41B   (1.26B to 1.51B) | 1.43B   (1.29B to 1.54B) | 2.2B   (2.01B to 2.34B) |
| ***Vaccine costs ($)*** | 163M   (148M to 175M) | 238M   (224M to 250M) | 443M   (432M to 453M) | 1.91B   (1.9B to 1.92B) | 68.2B   (68.2B to 68.3B) |
| ***Cost per DALY averted ($)*** | 18.4k   (5.93k to 50.8k) | 242   (-8.44k to 8.24k) | 2.55k   (-7.41k to 14.2k) | 57.4k   (35.5k to 158k) | 2.03M   (1.45M to 5.01M) |
| ***Benefit-cost ratio*** | 3.44   (2.97 to 3.98) | 5.36   (4.7 to 6.34) | 4.03   (3.54 to 4.8) | 0.95   (0.84 to 1.12) | 0.04   (0.03 to 0.04) |
| ***South East Asia Region (n=6 countries)*** | | | | | |
| ***Vaccines administered*** | 450k   (34.8k to 1.12M) | 1.09M   (690k to 1.71M) | 2.62M   (2.32M to 3.05M) | 82.5M   (82.2M to 83M) | 2.27B   (2.27B to 2.27B) |
| ***Vaccine-averted HCV infections*** | 35.5k   (4.71k to 85.8k) | 82.3k   (68.8k to 113k) | 164k   (155k to 195k) | 246k   (235k to 282k) | 1.5M   (1.4M to 1.67M) |
| ***Vaccine-averted HCC cases*** | 17   (2 to 52) | 100   (27 to 183) | 117   (41 to 209) | 158   (57 to 272) | 2.34k   (496 to 4.09k) |
| ***Vaccine-averted HCV deaths*** | 30   (3 to 90) | 188   (56 to 342) | 306   (118 to 487) | 408   (166 to 640) | 4.75k   (1.07k to 8.3k) |
| ***Vaccine-averted DALYs*** | 141   (13 to 443) | 916   (283 to 1.78k) | 1.53k   (587 to 2.47k) | 1.87k   (758 to 3.02k) | 19.3k   (4.45k to 34.5k) |
| ***Vaccine-averted direct costs ($)*** | 109k   (-154k to 859k) | 1.05M   (664k to 3.01M) | 3.57M   (2.5M to 8.78M) | 3.59M   (2.51M to 9.07M) | 9.49M   (5.17M to 24.6M) |
| ***Vaccine-attributable productivity gains ($)*** | 7.59M   (630k to 18.7M) | 29.7M   (25.6M to 37.9M) | 44.2M   (39.9M to 52.8M) | 66.2M   (61.4M to 76.3M) | 591M   (528M to 676M) |
| ***Vaccine costs ($)*** | 2.24M   (178k to 5.54M) | 6.74M   (4.79M to 9.62M) | 15.8M   (14.3M to 17.8M) | 561M   (559M to 563M) | 17B   (17B to 17B) |
| ***Cost per DALY averted ($)*** | 15.1k   (7.29k to 45.1k) | 6.21k   (2.26k to 19.6k) | 7.95k   (3.57k to 17.7k) | 298k   (183k to 732k) | 882k   (493k to 3.82M) |
| ***Benefit-cost ratio*** | 3.44   (2.63 to 5.34) | 4.56   (3.53 to 7.01) | 3.03   (2.72 to 3.89) | 0.12   (0.12 to 0.15) | 0.04   (0.03 to 0.04) |
| ***Western Pacific Region (n=14 countries)*** | | | | | |
| ***Vaccines administered*** | 2.69M   (1.2M to 3.8M) | 3.99M   (2.6M to 5.03M) | 12.4M   (11.8M to 12.9M) | 59.8M   (59.3M to 60.3M) | 2.1B   (2.1B to 2.1B) |
| ***Vaccine-averted HCV infections*** | 265k   (116k to 379k) | 388k   (291k to 463k) | 463k   (420k to 496k) | 488k   (445k to 522k) | 762k   (714k to 811k) |
| ***Vaccine-averted HCC cases*** | 309   (90 to 500) | 714   (287 to 1.02k) | 765   (328 to 999) | 778   (332 to 1.02k) | 858   (278 to 1.21k) |
| ***Vaccine-averted HCV deaths*** | 273   (90 to 416) | 649   (265 to 906) | 1.07k   (422 to 1.42k) | 1.1k   (451 to 1.45k) | 1.72k   (718 to 2.4k) |
| ***Vaccine-averted DALYs*** | 2.1k   (625 to 3.42k) | 5.43k   (2.19k to 7.76k) | 7.61k   (3.07k to 10.3k) | 7.71k   (3.17k to 10.5k) | 8.32k   (2.68k to 12k) |
| ***Vaccine-averted direct costs ($)*** | 35.7M   (21.3M to 67M) | 52.9M   (36.1M to 94M) | 97.4M   (71.6M to 163M) | 97.6M   (71.7M to 163M) | 98.7M   (69.8M to 172M) |
| ***Vaccine-attributable productivity gains ($)*** | 401M   (266M to 521M) | 590M   (480M to 693M) | 612M   (518M to 697M) | 623M   (529M to 707M) | 896M   (788M to 1B) |
| ***Vaccine costs ($)*** | 60.7M   (40.9M to 75.7M) | 87.3M   (69.7M to 101M) | 197M   (189M to 203M) | 928M   (921M to 935M) | 44.9B   (44.9B to 44.9B) |
| ***Cost per DALY averted ($)*** | 11.9k   (-4.15k to 38k) | 6.33k   (-1.67k to 19.4k) | 13k   (3.73k to 38.4k) | 108k   (74.8k to 264k) | 5.39M   (3.73M to 16.8M) |
| ***Benefit-cost ratio*** | 7.2   (5.76 to 9.17) | 7.36   (6.07 to 9.28) | 3.61   (3.07 to 4.31) | 0.78   (0.66 to 0.92) | 0.02   (0.02 to 0.03) |

# Section E: Country-level benefit-cost ratios

**Table 7. Benefit-cost ratios for the five scenarios per country.**

| **Benefit-cost ratios**  **2026–2050** | **Scenario 1** | **Scenario 2** | **Scenario 3** | **Scenario 4** | **Scenario 5** |
| --- | --- | --- | --- | --- | --- |
| **Afghanistan** | 0.34   (0.15 to 0.41) | 0.85   (0.73 to 1.01) | 0.26   (0.19 to 0.37) | 0.03   (0.02 to 0.04) | 0.00   (0.00 to 0.01) |
| **Albania** | 4.62   (3.97 to 5.56) | 3.96   (3.39 to 4.86) | 0.82   (0.71 to 0.98) | 0.53   (0.48 to 0.60) | 0.20   (0.17 to 0.23) |
| **Algeria** | 4.55   (3.92 to 5.59) | 3.60   (3.04 to 4.50) | 2.58   (2.19 to 3.23) | 0.14   (0.12 to 0.16) | 0.04   (0.03 to 0.04) |
| **Argentina** | 18.23   (15.95 to 21.50) | 16.20   (13.89 to 19.62) | 12.20   (10.43 to 14.72) | 0.25   (0.23 to 0.29) | 0.10   (0.09 to 0.11) |
| **Armenia** | 12.37   (10.92 to 14.15) | 11.72   (10.21 to 13.69) | 6.57   (5.71 to 7.71) | 2.06   (1.85 to 2.37) | 0.30   (0.27 to 0.34) |
| **Australia** | 9.03   (6.49 to 11.98) | 7.30   (5.17 to 9.91) | 4.59   (3.29 to 6.33) | 0.87   (0.63 to 1.19) | 0.06   (0.04 to 0.08) |
| **Austria** | 9.62   (8.00 to 11.76) | 8.83   (7.33 to 11.03) | 4.55   (3.78 to 5.59) | 0.58   (0.49 to 0.71) | 0.03   (0.02 to 0.03) |
| **Azerbaijan** | 11.56   (10.36 to 13.00) | 17.71   (15.68 to 20.34) | 8.69   (7.71 to 10.15) | 2.63   (2.38 to 3.03) | 0.20   (0.19 to 0.23) |
| **Bahrain** | 17.44   (16.41 to 18.35) | 24.22   (22.36 to 27.09) | 17.46   (16.06 to 19.75) | 0.06   (0.05 to 0.06) | 0.00   (0.00 to 0.01) |
| **Bangladesh** | 6.24   (5.19 to 7.42) | 8.46   (6.69 to 11.20) | 5.56   (4.38 to 6.69) | 0.27   (0.23 to 0.31) | 0.09   (0.08 to 0.10) |
| **Belgium** | 25.04   (22.85 to 28.76) | 26.14   (23.16 to 30.96) | 22.31   (19.62 to 26.66) | 0.49   (0.44 to 0.58) | 0.03   (0.02 to 0.03) |
| **Benin** | 0.47   (0.22 to 0.54) | 1.01   (0.88 to 1.16) | 0.35   (0.32 to 0.41) | 0.06   (0.06 to 0.07) | 0.05   (0.05 to 0.06) |
| **Bhutan** | 9.06   (7.66 to 11.40) | 13.12   (10.96 to 19.62) | 9.81   (8.25 to 15.60) | 0.14   (0.11 to 0.20) | 0.03   (0.02 to 0.05) |
| **Brazil** | 5.96   (4.65 to 7.69) | 6.46   (4.81 to 8.43) | 3.56   (2.60 to 4.58) | 0.57   (0.42 to 0.72) | 0.02   (0.02 to 0.03) |
| **Bulgaria** | 8.24   (7.24 to 9.56) | 9.29   (7.57 to 11.26) | 7.67   (6.16 to 9.53) | 1.63   (1.32 to 1.99) | 0.03   (0.03 to 0.04) |
| **Burkina Faso** | 4.54   (3.00 to 5.31) | 6.98   (5.92 to 8.43) | 1.45   (1.18 to 1.77) | 0.06   (0.05 to 0.07) | 0.05   (0.04 to 0.06) |
| **Burundi** | 0.02   (-0.02 to 0.02) | 0.04   (0.04 to 0.04) | 0.02   (0.01 to 0.02) | 0.03   (0.03 to 0.04) | 0.03   (0.03 to 0.04) |
| **Cambodia** | 2.61   (2.21 to 3.17) | 2.58   (2.16 to 3.11) | 1.92   (1.62 to 2.34) | 0.12   (0.11 to 0.14) | 0.09   (0.08 to 0.10) |
| **Cameroon** | 0.85   (0.68 to 1.04) | 1.43   (1.17 to 1.76) | 1.24   (1.02 to 1.52) | 0.04   (0.03 to 0.04) | 0.04   (0.03 to 0.04) |
| **Canada** | 14.71   (13.07 to 17.20) | 15.47   (13.73 to 18.23) | 14.48   (12.93 to 17.06) | 0.86   (0.78 to 1.00) | 0.05   (0.05 to 0.06) |
| **Central African Republic** | 0.00   (0.00 to 0.00) | 0.00   (-0.00 to 0.00) | -0.04   (-0.08 to -0.04) | 0.02   (0.02 to 0.03) | 0.02   (0.02 to 0.03) |
| **Chad** | 0.16   (-0.05 to 0.21) | 0.22   (0.18 to 0.35) | 0.13   (0.09 to 0.18) | 0.01   (0.01 to 0.01) | 0.01   (0.01 to 0.01) |
| **Chile** | 0.14   (0.12 to 0.17) | 0.23   (0.20 to 0.27) | 0.07   (0.06 to 0.09) | 0.01   (0.01 to 0.01) | 0.00   (0.00 to 0.00) |
| **China** | 7.48   (6.15 to 9.25) | 9.15   (7.61 to 12.25) | 3.05   (2.57 to 3.55) | 0.92   (0.77 to 1.06) | 0.02   (0.02 to 0.02) |
| **Colombia** | 3.58   (3.14 to 4.46) | 2.16   (1.89 to 2.69) | 1.17   (1.02 to 1.47) | 0.20   (0.18 to 0.24) | 0.03   (0.03 to 0.04) |
| **Croatia** | 10.24   (8.96 to 11.76) | 11.86   (9.77 to 14.78) | 4.12   (3.44 to 5.59) | 0.52   (0.44 to 0.70) | 0.01   (0.01 to 0.01) |
| **Cuba** | 18.85   (16.12 to 22.17) | 13.77   (11.39 to 16.64) | 10.47   (8.53 to 12.87) | 3.56   (2.95 to 4.32) | 0.11   (0.10 to 0.13) |
| **Czech Republic** | 8.39   (7.50 to 9.67) | 8.16   (7.19 to 9.50) | 5.87   (5.16 to 6.84) | 2.21   (1.97 to 2.53) | 0.05   (0.05 to 0.06) |
| **Denmark** | 0.04   (0.02 to 0.07) | 0.04   (0.03 to 0.08) | 0.04   (0.02 to 0.06) | 0.01   (0.01 to 0.02) | -0.00   (-0.00 to -0.00) |
| **Dominican Republic** | 1.46   (1.29 to 1.68) | 2.14   (1.90 to 2.47) | 1.02   (0.92 to 1.17) | 0.19   (0.18 to 0.22) | 0.07   (0.07 to 0.09) |
| **DR Congo** | 4.05   (-295.58 to 4.48) | 7.80   (6.84 to 8.91) | 7.44   (6.54 to 8.47) | 0.05   (0.04 to 0.05) | 0.01   (0.01 to 0.02) |
| **Ecuador** | 12.44   (11.20 to 14.17) | 13.96   (12.39 to 16.31) | 11.20   (9.92 to 13.14) | 0.20   (0.19 to 0.23) | 0.05   (0.05 to 0.06) |
| **Egypt** | 13.17   (10.73 to 16.97) | 8.02   (6.28 to 10.63) | 7.28   (5.67 to 9.66) | 0.67   (0.31 to 1.11) | 0.26   (0.02 to 0.57) |
| **El Salvador** | 6.24   (5.18 to 7.48) | 8.39   (6.84 to 10.05) | 5.08   (4.11 to 6.12) | 0.17   (0.14 to 0.20) | 0.03   (0.03 to 0.03) |
| **Estonia** | 13.69   (11.92 to 15.61) | 16.41   (13.41 to 19.58) | 10.98   (9.15 to 13.05) | 1.71   (1.44 to 2.01) | 0.10   (0.09 to 0.12) |
| **Ethiopia** | 0.02   (0.02 to 0.03) | 0.04   (0.04 to 0.05) | 0.01   (0.01 to 0.02) | 0.03   (0.03 to 0.03) | 0.03   (0.02 to 0.03) |
| **Fiji** | 0.20   (0.00 to 0.23) | 0.10   (0.00 to 0.11) | 0.05   (0.00 to 0.06) | 0.02   (0.00 to 0.02) | 0.01   (0.00 to 0.01) |
| **Finland** | 12.66   (11.46 to 14.40) | 14.66   (13.27 to 16.83) | 9.46   (8.66 to 10.78) | 0.63   (0.58 to 0.71) | 0.04   (0.03 to 0.04) |
| **France** | 6.30   (5.62 to 7.18) | 8.50   (7.48 to 9.99) | 1.73   (1.54 to 1.99) | 0.45   (0.40 to 0.51) | 0.01   (0.01 to 0.01) |
| **Gabon** | 0.46   (0.26 to 0.51) | 0.79   (0.71 to 0.92) | 0.49   (0.45 to 0.55) | 0.69   (0.61 to 0.78) | 0.67   (0.60 to 0.76) |
| **Gambia** | 0.05   (0.04 to 0.06) | 0.09   (0.07 to 0.12) | 0.02   (-0.00 to 0.03) | 0.04   (0.03 to 0.04) | 0.03   (0.03 to 0.04) |
| **Georgia** | 0.12   (0.10 to 0.20) | 0.11   (0.09 to 0.18) | 0.10   (0.08 to 0.14) | 0.19   (0.16 to 0.25) | 0.35   (0.28 to 0.44) |
| **Germany** | 13.57   (12.33 to 15.48) | 16.53   (14.74 to 19.42) | 5.66   (5.06 to 6.53) | 1.18   (1.07 to 1.36) | 0.02   (0.02 to 0.03) |
| **Ghana** | N/A | 14.94   (13.72 to 15.94) | 4.48   (4.15 to 5.30) | 0.36   (0.34 to 0.41) | 0.11   (0.10 to 0.13) |
| **Greece** | 4.62   (3.99 to 5.80) | 3.08   (2.61 to 3.93) | 2.38   (2.01 to 3.04) | 0.23   (0.21 to 0.29) | 0.04   (0.04 to 0.05) |
| **Hungary** | 0.50   (0.18 to 1.12) | 0.34   (0.14 to 0.69) | 0.29   (0.13 to 0.58) | 0.04   (0.03 to 0.06) | 0.02   (0.02 to 0.02) |
| **Iceland** | 16.23   (14.53 to 18.83) | 16.92   (15.25 to 19.63) | 15.02   (13.99 to 16.94) | 0.69   (0.65 to 0.77) | 0.04   (0.04 to 0.05) |
| **India** | 2.65   (-5.33 to 3.77) | 3.65   (2.85 to 5.98) | 2.30   (2.00 to 3.29) | 0.13   (0.12 to 0.17) | 0.03   (0.03 to 0.04) |
| **Indonesia** | 10.84   (-53.83 to 12.26) | 15.74   (13.13 to 18.66) | 3.78   (3.40 to 4.63) | 0.09   (0.08 to 0.10) | 0.03   (0.03 to 0.04) |
| **Iran** | 1.61   (1.20 to 2.15) | 1.39   (1.10 to 2.36) | 0.98   (0.78 to 1.53) | 0.20   (0.17 to 0.29) | 0.01   (0.01 to 0.02) |
| **Iraq** | 11.60   (10.43 to 12.86) | 21.38   (19.13 to 23.83) | 14.60   (13.11 to 16.42) | 0.18   (0.17 to 0.19) | 0.05   (0.04 to 0.06) |
| **Ireland** | 37.36   (34.69 to 40.60) | 46.35   (41.74 to 52.54) | 22.00   (19.89 to 25.74) | 1.19   (1.09 to 1.37) | 0.08   (0.07 to 0.09) |
| **Israel** | 3.79   (2.81 to 5.09) | 1.95   (1.46 to 2.69) | 1.40   (1.06 to 1.89) | 0.12   (0.10 to 0.15) | 0.04   (0.04 to 0.05) |
| **Italy** | 13.23   (11.93 to 15.40) | 14.23   (12.67 to 16.98) | 9.57   (8.46 to 11.46) | 3.21   (2.86 to 3.81) | 0.06   (0.05 to 0.07) |
| **Japan** | 17.42   (15.73 to 19.42) | 22.68   (19.55 to 27.16) | 12.66   (10.40 to 16.32) | 1.27   (1.06 to 1.61) | 0.02   (0.02 to 0.03) |
| **Jordan** | 11.74   (10.06 to 13.95) | 14.15   (11.79 to 17.78) | 11.09   (9.28 to 13.56) | 0.26   (0.23 to 0.30) | 0.03   (0.03 to 0.03) |
| **Kazakhstan** | 12.25   (6.81 to 13.75) | 19.47   (16.89 to 22.14) | 4.99   (4.44 to 5.59) | 0.94   (0.85 to 1.04) | 0.25   (0.22 to 0.29) |
| **Kenya** | 5.00   (2.26 to 5.81) | 9.34   (8.02 to 10.79) | 3.99   (3.47 to 4.75) | 0.16   (0.15 to 0.18) | 0.09   (0.08 to 0.10) |
| **Kyrgyzstan** | 0.89   (0.33 to 1.02) | 1.59   (1.41 to 1.80) | 0.53   (0.48 to 0.60) | 0.29   (0.26 to 0.34) | 0.23   (0.20 to 0.27) |
| **Latvia** | 0.12   (0.06 to 0.30) | 0.13   (0.06 to 0.28) | 0.12   (0.06 to 0.27) | 0.19   (0.15 to 0.27) | 0.17   (0.14 to 0.20) |
| **Lebanon** | 0.29   (0.26 to 0.35) | 0.56   (0.49 to 0.71) | 0.15   (0.11 to 0.20) | 0.04   (0.04 to 0.06) | 0.01   (0.01 to 0.02) |
| **Libya** | 23.10   (20.45 to 28.42) | 21.68   (18.49 to 28.56) | 19.06   (16.32 to 25.01) | 0.52   (0.46 to 0.63) | 0.27   (0.24 to 0.33) |
| **Lithuania** | 16.07   (13.08 to 19.09) | 17.96   (13.23 to 22.67) | 15.05   (11.20 to 18.94) | 1.77   (1.34 to 2.19) | 0.04   (0.04 to 0.05) |
| **Luxembourg** | 48.06   (43.50 to 54.55) | 54.07   (47.71 to 63.49) | 37.32   (32.01 to 45.93) | 8.66   (7.46 to 10.59) | 0.20   (0.18 to 0.24) |
| **Madagascar** | 0.04   (0.01 to 0.05) | 0.06   (0.05 to 0.08) | 0.04   (0.03 to 0.05) | 0.01   (0.01 to 0.01) | 0.00   (0.00 to 0.01) |
| **Malaysia** | 1.44   (1.17 to 1.88) | 1.65   (1.37 to 2.18) | 0.53   (0.45 to 0.65) | 0.08   (0.07 to 0.09) | 0.01   (0.01 to 0.01) |
| **Malta** | 11.44   (10.21 to 12.84) | 12.66   (11.16 to 14.35) | 5.94   (5.24 to 6.73) | 0.39   (0.34 to 0.43) | 0.02   (0.02 to 0.02) |
| **Mexico** | 44.67   (41.13 to 49.57) | 52.25   (45.70 to 60.83) | 39.66   (34.95 to 46.26) | 2.73   (2.39 to 3.16) | 0.11   (0.10 to 0.12) |
| **Moldova** | 6.36   (5.25 to 7.71) | 4.45   (3.65 to 5.40) | 3.11   (2.57 to 3.76) | 1.60   (1.41 to 1.87) | 0.51   (0.43 to 0.61) |
| **Mongolia** | 2.90   (2.30 to 3.34) | 4.46   (3.90 to 5.15) | 1.92   (1.71 to 2.20) | 0.43   (0.36 to 0.51) | 0.31   (0.26 to 0.36) |
| **Morocco** | 2.33   (1.97 to 2.82) | 3.52   (2.95 to 4.26) | 2.15   (1.82 to 2.65) | 0.18   (0.16 to 0.22) | 0.03   (0.02 to 0.03) |
| **Mozambique** | 1.14   (-0.33 to 1.34) | 2.09   (1.74 to 2.57) | 0.75   (0.63 to 1.03) | 0.03   (0.03 to 0.04) | 0.03   (0.02 to 0.03) |
| **Nepal** | 1.82   (1.62 to 2.14) | 1.96   (1.74 to 2.28) | 1.35   (1.20 to 1.62) | 0.13   (0.12 to 0.15) | 0.04   (0.03 to 0.05) |
| **Netherlands** | 25.56   (23.50 to 28.49) | 32.63   (29.44 to 37.74) | 20.34   (18.38 to 23.38) | 0.27   (0.25 to 0.30) | 0.01   (0.01 to 0.01) |
| **New Zealand** | 1.39   (1.15 to 1.70) | 1.31   (1.09 to 1.60) | 0.85   (0.70 to 1.03) | 0.15   (0.13 to 0.18) | 0.01   (0.01 to 0.01) |
| **Nigeria** | 1.41   (0.98 to 1.76) | 2.12   (1.65 to 2.86) | 0.76   (0.60 to 0.95) | 0.06   (0.05 to 0.06) | 0.05   (0.04 to 0.06) |
| **Norway** | 9.95   (8.74 to 11.44) | 12.55   (10.98 to 14.59) | 3.40   (2.98 to 3.93) | 0.22   (0.19 to 0.25) | 0.01   (0.01 to 0.01) |
| **Oman** | 14.76   (13.45 to 16.35) | 21.47   (17.61 to 26.11) | 15.26   (12.90 to 18.37) | 0.16   (0.14 to 0.19) | 0.01   (0.00 to 0.01) |
| **Pakistan** | 1.25   (-9.92 to 1.46) | 2.42   (2.12 to 2.78) | 0.90   (0.78 to 1.05) | 0.27   (0.24 to 0.31) | 0.23   (0.20 to 0.27) |
| **Panama** | 11.26   (10.57 to 12.17) | 12.49   (11.09 to 14.29) | 9.68   (8.69 to 11.06) | 0.09   (0.08 to 0.10) | 0.01   (0.01 to 0.01) |
| **Papua New Guinea** | 0.31   (0.26 to 0.36) | 0.51   (0.43 to 0.60) | 0.21   (0.17 to 0.24) | 0.11   (0.10 to 0.13) | 0.10   (0.09 to 0.11) |
| **Peru** | 0.47   (0.42 to 0.55) | 0.71   (0.64 to 0.84) | 0.40   (0.36 to 0.47) | 0.04   (0.04 to 0.05) | 0.03   (0.02 to 0.03) |
| **Philippines** | 7.69   (6.18 to 9.16) | 13.35   (11.13 to 15.90) | 9.55   (8.02 to 11.44) | 0.09   (0.09 to 0.11) | 0.04   (0.04 to 0.05) |
| **Poland** | 0.53   (0.41 to 0.72) | 0.39   (0.31 to 0.51) | 0.19   (0.15 to 0.24) | 0.11   (0.09 to 0.15) | 0.00   (0.00 to 0.00) |
| **Portugal** | 15.93   (14.10 to 18.47) | 18.95   (15.81 to 23.33) | 12.68   (10.51 to 16.03) | 0.78   (0.65 to 0.97) | 0.02   (0.01 to 0.02) |
| **Puerto Rico** | 12.77   (11.40 to 15.46) | 13.30   (11.25 to 17.11) | 6.67   (5.61 to 8.70) | 4.00   (3.40 to 5.19) | 0.10   (0.09 to 0.13) |
| **Qatar** | 44.24   (41.12 to 49.25) | 53.29   (46.39 to 65.17) | 36.35   (31.59 to 44.61) | 1.19   (1.02 to 1.47) | 0.02   (0.01 to 0.02) |
| **Republic of Korea** | 0.49   (0.23 to 1.21) | 0.48   (0.26 to 1.12) | 0.18   (0.10 to 0.36) | 0.06   (0.03 to 0.11) | 0.00   (0.00 to 0.00) |
| **Romania** | 7.92   (6.89 to 9.30) | 8.33   (6.97 to 9.94) | 7.27   (6.10 to 8.73) | 2.41   (2.05 to 2.87) | 0.06   (0.05 to 0.07) |
| **Russian Federation** | 6.36   (5.76 to 7.22) | 8.60   (7.36 to 10.45) | 6.00   (5.13 to 7.43) | 2.31   (1.99 to 2.82) | 0.08   (0.07 to 0.09) |
| **Rwanda** | 0.28   (0.18 to 0.46) | 0.31   (0.22 to 0.55) | 0.27   (0.20 to 0.47) | 0.05   (0.04 to 0.06) | 0.05   (0.04 to 0.05) |
| **Saudi Arabia** | 10.92   (8.81 to 13.09) | 12.82   (9.60 to 16.19) | 10.81   (8.13 to 13.22) | 0.11   (0.08 to 0.13) | 0.01   (0.01 to 0.01) |
| **Slovakia** | 1.04   (0.84 to 1.36) | 0.99   (0.80 to 1.28) | 0.59   (0.48 to 0.76) | 0.23   (0.19 to 0.29) | 0.01   (0.00 to 0.01) |
| **Slovenia** | 3.30   (2.67 to 4.08) | 3.17   (2.60 to 3.94) | 1.27   (1.04 to 1.56) | 0.31   (0.26 to 0.39) | 0.01   (0.00 to 0.01) |
| **South Africa** | 10.51   (6.70 to 12.06) | 12.64   (10.68 to 17.25) | 8.65   (7.45 to 10.68) | 0.39   (0.35 to 0.45) | 0.13   (0.12 to 0.14) |
| **Spain** | 7.91   (1.08 to 12.32) | 7.77   (1.03 to 12.22) | 7.44   (0.96 to 11.98) | 0.52   (0.07 to 0.74) | 0.01   (0.00 to 0.01) |
| **Sudan** | 5.78   (5.02 to 6.65) | 10.06   (8.67 to 11.90) | 7.61   (6.65 to 8.86) | 0.20   (0.18 to 0.23) | 0.15   (0.13 to 0.17) |
| **Sweden** | 26.21   (24.04 to 29.84) | 28.14   (25.26 to 32.90) | 25.81   (23.06 to 30.70) | 0.23   (0.21 to 0.27) | 0.02   (0.02 to 0.02) |
| **Switzerland** | 31.12   (28.88 to 35.29) | 33.91   (31.10 to 39.51) | 24.43   (22.34 to 28.82) | 4.13   (3.80 to 4.82) | 0.08   (0.07 to 0.09) |
| **Syria** | 1.24   (1.06 to 1.68) | 1.29   (1.08 to 1.81) | 0.80   (0.66 to 1.15) | 0.20   (0.18 to 0.26) | 0.17   (0.14 to 0.21) |
| **Taiwan** | 8.17   (6.55 to 10.84) | 9.24   (7.82 to 11.96) | 5.24   (4.55 to 6.30) | 1.27   (1.13 to 1.46) | 0.03   (0.03 to 0.03) |
| **Tajikistan** | 0.32   (0.02 to 0.37) | 0.74   (0.67 to 0.82) | 0.24   (0.23 to 0.29) | 0.15   (0.14 to 0.18) | 0.12   (0.11 to 0.14) |
| **Thailand** | 7.88   (4.51 to 10.49) | 8.34   (6.64 to 11.78) | 5.74   (4.68 to 7.63) | 0.45   (0.39 to 0.56) | 0.03   (0.03 to 0.04) |
| **Tunisia** | 7.37   (6.18 to 9.19) | 8.16   (6.71 to 10.62) | 6.63   (5.47 to 8.53) | 0.31   (0.27 to 0.39) | 0.05   (0.04 to 0.05) |
| **Turkey** | 16.90   (14.68 to 19.51) | 23.24   (20.01 to 27.35) | 12.11   (10.46 to 14.44) | 0.50   (0.44 to 0.59) | 0.03   (0.03 to 0.03) |
| **Uganda** | 0.36   (0.11 to 0.43) | 0.82   (0.70 to 0.97) | 0.49   (0.42 to 0.58) | 0.06   (0.05 to 0.07) | 0.05   (0.05 to 0.06) |
| **Ukraine** | 2.49   (2.21 to 3.00) | 1.68   (1.49 to 2.04) | 1.03   (0.91 to 1.23) | 0.78   (0.71 to 0.91) | 0.23   (0.21 to 0.26) |
| **United Arab Emirates** | 31.17   (29.06 to 35.26) | 34.33   (30.14 to 41.85) | 29.47   (25.84 to 36.14) | 0.30   (0.23 to 0.38) | 0.02   (0.01 to 0.02) |
| **United Kingdom** | 0.57   (0.31 to 0.99) | 0.61   (0.35 to 1.00) | 0.40   (0.26 to 0.57) | 0.07   (0.04 to 0.10) | 0.00   (0.00 to 0.01) |
| **United Republic of Tanzania** | 0.17   (0.10 to 0.21) | 0.40   (0.27 to 0.48) | 0.15   (0.10 to 0.18) | 0.01   (0.01 to 0.01) | 0.01   (0.00 to 0.01) |
| **United States of America** | 2.08   (1.44 to 3.03) | 1.72   (1.23 to 2.49) | 0.97   (0.75 to 1.28) | 0.38   (0.29 to 0.50) | 0.03   (0.02 to 0.04) |
| **Uzbekistan** | 1.49   (-1.63 to 1.77) | 2.42   (2.05 to 2.87) | 0.82   (0.72 to 0.98) | 0.05   (0.05 to 0.06) | 0.03   (0.03 to 0.04) |
| **Vanuatu** | 7.92   (6.52 to 9.68) | 8.23   (6.67 to 10.00) | 4.98   (4.45 to 5.88) | 0.22   (0.17 to 0.25) | 0.15   (0.11 to 0.17) |
| **Venezuela** | 31.52   (29.44 to 34.32) | 32.99   (30.17 to 36.89) | 14.73   (13.28 to 16.78) | 1.47   (1.35 to 1.64) | 0.16   (0.14 to 0.18) |
| **Viet Nam** | 6.56   (5.88 to 7.29) | 11.56   (10.38 to 12.87) | 2.02   (1.80 to 2.43) | 0.57   (0.51 to 0.67) | 0.09   (0.08 to 0.10) |
| **Yemen** | 0.45   (0.37 to 0.60) | 0.48   (0.39 to 0.69) | 0.43   (0.35 to 0.60) | 0.01   (0.01 to 0.02) | 0.01   (0.01 to 0.01) |

**Table 8. Correlation between benefit-cost ratios and predictors.** Correlation was computed for all countries and is reported as Pearson r (p-value). Predictors included Gross Domestic Product (GDP) per capita, RNA prevalence among people who inject drugs and proportion of people who inject drugs ever tested or treated.

| **r (p-value)** | **Scenario 1** | **Scenario 2** | **Scenario 3** | **Scenario 4** | **Scenario 5** |
| --- | --- | --- | --- | --- | --- |
| **GDP per capita** | 0.585  **(0.0)** | 0.553  **(0.0)** | 0.528  **(0.0)** | 0.452  **(0.0)** | -0.16  (0.0854) |
| **RNA prevalence** | 0.397  **(0.0)** | 0.374  **(0.0)** | 0.369  **(0.0)** | 0.315  **(0.0006)** | 0.029  (0.7566) |
| **Proportion ever tested** | 0.332  **(0.0003)** | 0.253  **(0.0062)** | 0.284  **(0.002)** | 0.286  **(0.0019)** | -0.072  (0.4405) |
| **Proportion ever treated** | 0.228  **(0.0139)** | 0.161  (0.0841) | 0.169  (0.0689) | 0.172  (0.0653) | -0.086  (0.3566) |

# References

1.	United Nations. World Population Prospects 2024, Online Edition: Department of Economic and Social Affairs, Population Division2024.

2.	The CDA Foundation. Hepatitis C – All Countries. Lafayette, CO: CDA Foundation; 2025 [July 2025]; Available from: https://cdafound.org/polaris/database-query/.

3.	Vollset SE, Ababneh HS, Abate YH, Abbafati C, Abbasgholizadeh R, Abbasian M, et al. Burden of disease scenarios for 204 countries and territories, 2022–2050: a forecasting analysis for the Global Burden of Disease Study 2021. The Lancet. 2024; 403:2204-56.

4.	Degenhardt L, Webb P, Colledge-Frisby S, Ireland J, Wheeler A, Ottaviano S, et al. Epidemiology of injecting drug use, prevalence of injecting-related harm, and exposure to behavioural and environmental risks among people who inject drugs: a systematic review. The Lancet Global Health. 2023; 11:e659-e72.

5.	Office for National Statistics. Estimates of the population for the UK, England, Wales, Scotland, and Northern Ireland. 2024.

6.	Grebely J, Larney S, Peacock A, Colledge S, Leung J, Hickman M, et al. Global, regional, and country-level estimates of hepatitis C infection among people who have recently injected drugs. Addiction. 2019; 114:150-66.

7.	Hajarizadeh B, Kairouz A, Ottaviano S, Ireland J, Willing A, Cunningham E, et al. Global, regional, and country-level coverage of testing and treatment for HIV and hepatitis C infection among people who inject drugs: a systematic review. 2023.

8.	Fair H, Walmsley R. World Prison Population List (14th edition). Technical Report. ICPR, London, UK 2024.

9.	Fair H, Walmsley R. World Female Imprisonment List (6th edition). Technical Report. ICPR, London, UK 2025.

10.	United Nations Office on Drugs and Crime. UNODC Research - Data Portal – Prisons and Prisoners. 2025.

11.	Council of Europe, Université de Lausanne. SPACE I Annual Reports. 2011, 2013..2015, 2018, 2021, 2023.

12.	Australian Bureau of Statistics. Corrective Services, Australia. 2025.

13.	Statistics Canada. Table 35-10-0014-01 Adult admissions to correctional services. 2024.

14.	Gendarmerie of Chile. Penitentiary Statistical Compendiums. 2000-2007.

15.	Ministry of Justice and Peace, General Directorate of Social Adaptation, National Institute of Criminology Research and Statistics Unit. Statistical Yearbooks: Compendium of Statistics of the Costa Rican Penitentiary System. Ministry of Justice and Peace; 2009-2023.

16.	The National Institute of Statistics and Geography. National Census of the Federal and State Penitentiary System 2022 Mexico: Mexican Government2023 9/6/2023.

17.	The Bureau of Justice Statistics in the US. Prisoners. Department of Justice; 2000-2022.

18.	Fazito E, Cuchi P, Mahy M, Brown T. Analysis of duration of risk behaviour for key populations: a literature review. Sexually Transmitted Infections. 2012; 88:i24.

19.	Zeremski M, Dimova RB, Pillardy J, de Jong YP, Jacobson IM, Talal AH. Fibrosis Progression in Patients With Chronic Hepatitis C Virus Infection. The Journal of Infectious Diseases. 2016; 214:1164-70.

20.	Fleming KM, Aithal GP, Card TR, West J. The rate of decompensation and clinical progression of disease in people with cirrhosis: a cohort study. Aliment Pharmacol Ther. 2010; 32:1343-50.

21.	Micallef JM, Kaldor JM, Dore GJ. Spontaneous viral clearance following acute hepatitis C infection: a systematic review of longitudinal studies. Journal of Viral Hepatitis. 2006; 13:34-41.

22.	Page K, Hahn JA, Evans J, Shiboski S, Lum P, Delwart E, et al. Acute Hepatitis C Virus Infection in Young Adult Injection Drug Users: A Prospective Study of Incident Infection, Resolution, and Reinfection. The Journal of Infectious Diseases. 2009; 200:1216-26.

23.	van den Berg CHBS, Grady BPX, Schinkel J, van de Laar T, Molenkamp R, van Houdt R, et al. Female Sex and IL28B, a Synergism for Spontaneous Viral Clearance in Hepatitis C Virus (HCV) Seroconverters from a Community-Based Cohort. PLOS ONE. 2011; 6:e27555.

24.	Aisyah DN, Shallcross L, Hully AJ, O'Brien A, Hayward A. Assessing hepatitis C spontaneous clearance and understanding associated factors—A systematic review and meta-analysis. Journal of Viral Hepatitis. 2018; 25:680-98.

25.	Hepatitis C Virus Infection Consensus Statement Working Group. Australian recommendations for the management of hepatitis C virus infection: a consensus statement [2022]. Melbourne: Gastroenterological Society of Australia; 2022 [cited 2025 Oct]; Available from: https://hepcguidelines.org.au/special-populations/treatment-of-decompensated-liver-disease/.

26.	Gao YX, Ning QQ, Yang PX, Guan YY, Liu PX, Liu ML, et al. Recent advances in recurrent hepatocellular carcinoma therapy. World J Hepatol. 2023; 15:460-76.

27.	Thompson AJ, Holmes JA. Treating hepatitis C - what's new? Aust Prescr. 2015; 38:191-7.

28.	Scott N, Kuschel C, Pedrana A, Schroeder S, Howell J, Thompson A, et al. A model of the economic benefits of global hepatitis C elimination: an investment case. Lancet Gastroenterol Hepatol. 2020; 5:940-7.

29.	Tordrup D, Hutin Y, Stenberg K, Lauer JA, Hutton DW, Toy M, et al. Cost-effectiveness of testing and treatment for hepatitis B virus and hepatitis C virus infections: an analysis by scenarios, regions, and income. Value in Health. 2020; 23:1552-60.

30.	Chhatwal J, Chen Q, Bethea ED, Ladd MA, Mueller PP, Hutin Y, et al. Hep C Calculator: an online tool for cost-effectiveness analysis of DAAs. The Lancet Gastroenterology & Hepatology. 2018; 3:819.

31.	United States Department of Labor. U.S. Bureau of Labor Statistics. United States government; [cited 2025 Oct]; Available from: https://www.bls.gov/.

32.	Clinton Health Access Initiative (CHAI). HCV Market Intelligence Report: Issue 3, 2023. [08 July, 2025)]; Available from: https://www.clintonhealthaccess.org/report/2023-hepatitis-c-market-intelligence-report/.

33.	Walker N, Tam Y, Friberg IK. Overview of the Lives Saved Tool (LiST). BMC Public Health. 2013; 13 Suppl 3:S1.

34.	World Bank Group. World Bank country classifications by income level for 2024-2025. 2024; Available from: https://blogs.worldbank.org/en/opendata/world-bank-country-classifications-by-income-level-for-2024-2025.

35.	World Health Organization. 2024 MI4A Public Vaccine Purchase Dataset. Geneva2024.

36.	World Bank Group. GDP per capita (current US$). 2025; Available from: https://data.worldbank.org/indicator/NY.GDP.PCAP.CD.

37.	World Bank Group. Employment to population ratio, 15+, total (%) (modeled ILO estimate). 2025; Available from: https://data.worldbank.org/indicator/SL.EMP.TOTL.SP.ZS.

38.	Scott N, Kuschel C, Pedrana A, Schroeder S, Howell J, Thompson A, et al. A model of the economic benefits of global hepatitis C elimination: an investment case. The Lancet Gastroenterology & Hepatology. 2020; 5:940-7.

39.	DiBonaventura M, Wagner JS, Yuan Y, L'Italien G, Langley P, Ray Kim W. The impact of hepatitis C on labor force participation, absenteeism, presenteeism and non-work activities. J Med Econ. 2011; 14:253-61.

40.	Younossi Z, Brown A, Buti M, Fagiuoli S, Mauss S, Rosenberg W, et al. Impact of eradicating hepatitis C virus on the work productivity of chronic hepatitis C (CH-C) patients: an economic model from five European countries. J Viral Hepat. 2016; 23:217-26.

41.	Martin NK, Devine A, Eaton JW, Miners A, Hallett TB, Foster GR, et al. Modeling the impact of early antiretroviral therapy for adults coinfected with HIV and hepatitis B or C in South Africa. Aids. 2014; 28 Suppl 1:S35-46.

42.	GBD 2021 Diseases and Injuries Collaborators. Global incidence, prevalence, years lived with disability (YLDs), disability-adjusted life-years (DALYs), and healthy life expectancy (HALE) for 371 diseases and injuries in 204 countries and territories and 811 subnational locations, 1990-2021: a systematic analysis for the Global Burden of Disease Study 2021. Lancet. 2024; 403:2133-61.

43.	World Health Organization. Global hepatitis report 2024: action for access in low- and middle-income countries. Geneva 2024. Licence: CC BY-NC-SA 3.0 IGO.

44.	Bartolomeo, N., Trerotoli, P. & Serio, G. Progression of liver cirrhosis to HCC: an application of hidden Markov model. BMC Med Res Methodol 11, 38 (2011). <https://doi.org/10.1186/1471-2288-11-38>

45.	Planas R, Ballesté B, Alvarez MA, Rivera M, Montoliu S, Galeras JA, Santos J, Coll S, Morillas RM, Solà R. Natural history of decompensated hepatitis C virus-related cirrhosis. A study of 200 patients. J Hepatol. 2004 May;40(5):823-30. doi: 10.1016/j.jhep.2004.01.005. PMID: 15094231.
